# Performance of serum apolipoprotein-A1 as a sentinel of Covid-19

**DOI:** 10.1371/journal.pone.0242306

**Published:** 2020-11-20

**Authors:** Thierry Poynard, Olivier Deckmyn, Marika Rudler, Valentina Peta, Yen Ngo, Mathieu Vautier, Sepideh Akhavan, Vincent Calvez, Clemence Franc, Jean Marie Castille, Fabienne Drane, Mehdi Sakka, Dominique Bonnefont-Rousselot, Jean Marc Lacorte, David Saadoun, Yves Allenbach, Olivier Benveniste, Frederique Gandjbakhch, Julien Mayaux, Olivier Lucidarme, Bruno Fautrel, Vlad Ratziu, Chantal Housset, Dominique Thabut, Patrice Cacoub

**Affiliations:** 1 Institute of Cardiometabolism and Nutrition (ICAN), Centre de Recherche Saint-Antoine (CRSA), INSERM, Sorbonne Université, Assistance Publique-Hôpitaux de Paris (AP-HP), Paris, France; 2 BioPredictive, Research, Paris, France; 3 Department of Hepatology, Sorbonne Université, AP-HP Pitié-Salpêtrière, Paris, France; 4 Department of Internal Medicine and Clinical Immunology, Sorbonne Université, AP-HP Pitié-Salpêtrière, Paris, France; 5 Department of Virology, Sorbonne Université, AP-HP Pitié-Salpêtrière, Paris, France; 6 Department of Metabolic Biochemistry, Sorbonne Université, AP-HP Pitié-Salpêtrière, Paris, France; 7 Department of Biochemistry, Endocrinology and Oncology, Sorbonne Université, AP-HP Pitié-Salpêtrière, Paris, France; 8 Department of Rhumatology, Sorbonne Université, AP-HP Pitié-Salpêtrière, Paris, France; 9 Department of Intensive Care, Sorbonne Université, AP-HP Pitié-Salpêtrière, Paris, France; 10 Department of Radiology, Sorbonne Université, AP-HP Pitié-Salpêtrière, Paris, France; 11 Institut of Cardiometabolism and Nutrition ICAN, Sorbonne Université, AP-HP Pitié-Salpêtrière, Paris, France; 12 INSERM, Sorbonne University, UMRS 1269 Nutriomique, service de Nutrition, APHP, Paris, France; Saint Louis University, UNITED STATES

## Abstract

**Background:**

Since 1920, a decrease in serum cholesterol has been identified as a marker of severe pneumonia. We have assessed the performance of serum apolipoprotein-A1, the main transporter of HDL-cholesterol, to identify the early spread of coronavirus disease 2019 (Covid-19) in the general population and its diagnostic performance for the Covid-19.

**Methods:**

We compared the daily mean serum apolipoprotein-A1 during the first 34 weeks of 2020 in a population that is routinely followed for a risk of liver fibrosis risk in the USA (212,297 serum) and in France (20,652 serum) in relation to a local increase in confirmed cases, and in comparison to the same period in 2019 (266,976 and 28,452 serum, respectively). We prospectively assessed the sensitivity of this marker in an observational study of 136 consecutive hospitalized cases and retrospectively evaluated its specificity in 7,481 controls representing the general population.

**Results:**

The mean serum apolipoprotein-A1 levels in the survey populations began decreasing in January 2020, compared to the same period in 2019. This decrease was highly correlated with the daily increase in confirmed Covid-19 cases in the following 34 weeks, both in France and USA, including the June and mid-July recovery periods in France. Apolipoprotein-A1 at the 1.25 g/L cutoff had a sensitivity of 90.6% (95%CI84.2–95.1) and a specificity of 96.1% (95.7–96.6%) for the diagnosis of Covid-19. The area under the characteristics curve was 0.978 (0.957–0.988), and outperformed haptoglobin and liver function tests. The adjusted risk ratio of apolipoprotein-A1 for survival without transfer to intensive care unit was 5.61 (95%CI 1.02–31.0; P = 0.04).

**Conclusion:**

Apolipoprotein-A1 could be a sentinel of the pandemic in existing routine surveillance of the general population. NCT01927133, CER-2020-14.

## Introduction

There is an urgent need to detect people in the general population who are at risk of being admitted to the hospital for Covid-19. Although viral nucleic acid testing and chest computed tomography are standard methods for diagnosing Covid-19 in patients with symptoms, these are time consuming. A review reported that there are only three models for predicting hospital admission in the healthy general population, and these are limited by a high risk of bias, and by using proxy outcomes [1].

In February 2020 we observed in Pitié-Salpêtrière hospital Paris, France (“APHP-PSL”), that patients hospitalized for Covid-19 had a specific profile of apolipoprotein-A1 decrease and haptoglobin increase. This profile was never observed before in our prospective data on apolipoprotein-A1 from and since the first cohort of alcoholic liver disease in 1982 (S1 File) nor in patients at risk of liver fibrosis followed with FibroTest, which include apolipoprotein-A1 in its components. Therefore, in the large cohorts of patients followed by FibroTest for a risk of liver fibrosis (named serum-cohorts) we aim to compare the daily value of serum apolipoprotein-A1, of the ongoing year 2020 to previous years “Covid-19 Free”.

Our hypothesis was supported by the literature reporting this profile of apolipoprotein-A1 in patients with viral pneumonia. In 1920, Harold A. Kipp found that a decrease in serum cholesterol was a marker of severe pneumonia [2]. One hundred years later, a meta-analysis confirmed that levels of high-density lipoprotein cholesterol (HDL) and a level below the median of its transporter, apolipoprotein-A1, were associated with a two-fold increase in mortality in patients with severe sepsis (S1 Fig, S1 Table) [3]. Its decrease seems to occur earlier than the increase in haptoglobin, another marker of sepsis, and independently of liver function tests [4, 5]. Recently, hypolipidemia was reported in Covid-19 patients with mild symptoms [6, 7]. Furthermore, unlike haptoglobin, which is synthesized mainly by the liver, apolipoprotein-A1 is synthesized by both the liver and the intestine [5]. This early decrease in apolipoprotein-A1 with normal liver function test and before the haptoglobin increase suggested an intestinal route of infection [8, 9] (S1 File).

Therefore, in the general population, apolipoprotein-A1 might be a sensitive marker of SARS-COV2 infection from apparently healthy carriers, to severe patients hospitalized with Covid-19. To support this hypothesis, we analyze apolipoprotein-A1 in large serum-cohorts and in Covid-19 patients.

In serum-cohorts, the first primary endpoint was to demonstrate a significant decrease of the mean daily value of apolipoprotein-A1 in the 34 weeks of the pandemic year 2020, *vs*. those of the year 2019. The second aim was to demonstrate the absence of significant confounding factors explaining such temporal association. Then, the third aim was to assess the dynamic temporal association of apolipoprotein-A1 values with the number of confirmed Covid-19 cases in SA and France.

In patients with Covid-19, the first aim was to evaluate the diagnostic performance of apolipoprotein-A1. For this purpose, the sensitivity was assessed in the hospitalized patients. The specificity was assessed in a previous prospective study performed in a representative sample of the French general population before the pandemic. Finally, the last aim was to evaluate the prognostic value of apolipoprotein-A1 in the hospitalized patients.

## Patients and methods

### Ethics

The prospective observational study in Covid-19 patients was approved by CER-Sorbonne University IRB, CER-2020-14, with a signed informed consent. All of the previously published patient analyses from retrospective databases were non-interventional studies, without supplementary blood samples, and were exempt from a review of the IRB (NCT01927133). The investigation was performed according to the principles of the Declaration of Helsinki. All authors had access to the study data and reviewed and approved the final manuscript.

### Significant decrease of apolipoprotein-A1 in 2020 serum-cohorts

We used three cohorts of serum from subjects at risk of liver fibrosis followed by FibroTest (FibroSure in USA) [10]. These serum-cohorts had three increasing levels of Covid-19 incidence in 2020. The large private-laboratories US cohort (“US-cohort”), has a lower risk of Covid-19 than the “(French-cohort”) following patients in academic hospital treating Covid-19 patients and private-laboratories, and a high-risk cohort which included patients at the Pitié-Salpêtrière hospital Paris, France (“APHP-PSL”), which was a Covid center. The core temporal analysis compared the first 34 weeks of US consecutive anonymous serum 2020 *vs*. the serum 2019. Details of serum-cohorts were given in S2 File.

### Confounding factors in serum-cohorts

Decrease in apolipoprotein-A1 may be due to direct liver toxicity from SARS-CoV-2 [11], but also to drug-induced liver disease (DILI) caused by medications (S2 Table). We analyzed the kinetics of alpha2-macroglobulin (A2M) a specific marker of liver fibrosis [10], and of haptoglobin, a sensitive biomarker of severe acute phase, as liver function tests, gammaglutamyl transpeptidase (GGT), alanine aminotransferase (ALT) and total bilirubin. In serum from subjects followed for non-alcoholic fatty liver disease (NAFLD) the kinetics of total cholesterol), triglycerides, fasting glucose, weight and height were also analyzed.

### Temporal association between daily apolipoprotein-A1 and spread of Covid-19

The number of confirmed Covid-19 cases in France and in the USA was assessed according to published data from the European Centre for Disease Prevention and Control (https://ourworldindata.org/coronavirus-data) (S2 Fig).

### Sensitivity and prognostic values in Covid-19 patients

Sensitivity and prognostic values were assessed in a prospective study of Covid-19 patients hospitalized in APHP-PSL. The primary endpoint was the survival without transfer in ICU at 28 days, adjusted on age, gender, haptoglobin, and liver tests.

### Specificity in general population: Specificity-cohorts

We previously collected five cohorts, names here “specificity cohorts” which allowed us to retrospectively validate the specificity of apolipoprotein-A1 in a large group of subjects without Covid-19 [12–16]. The measurements were all performed on fresh prospectively collected serum and analyzed in the biochemistry unit of the APHP-PSL hospital, with the same methods as the Covid-19 cases. The core control population for specificity assessment was a group of healthy volunteers that was representative of the French population (CPAM: Caisse Primaire Assurance Maladie) [16].

### Patients with diarrhea

In order to identify a profile of patients with a possible intestinal route of infection, we compared the subsets of patients with or without diarrhea.

### Biochemical and virological methods

Apolipoprotein-A1, haptoglobin, A2M, GGT, ALT and bilirubin were assessed following BioPredictive (Paris, France) analytical recommendations [17]. The virological methods for the diagnosis used to diagnose SARS-CoV2 in respiratory samples, were detailed in S2 File.

### Temporal association in Covid-19 patients

The kinetics of apolipoprotein A1 and haptoglobin were assessed prospectively in patients with repeated serum during the hospitalization.

### Statistical methods

The first primary endpoint was to demonstrate a significant decrease of the mean daily value of apolipoprotein-A1 in the 34 weeks of the year 2020, *vs*. those of the year 2019. We defined a significant decrease of apolipoprotein-A1 as below 1.25 g/L, the optimal cutoff defined by the highest Youden index (sensitivity + specificity -1) using the hospitalized Covid-19 patients for sensitivity, and the CPAM population representative of the general population for the specificity [16]. The daily proportion of serum below this cutoff defined a significant risk of Covid-19. The mean of apolipoprotein-A1 on all the 2019 serum of the US-cohort was taken as the reference in a large population without Covid-19. This allowed to assess the proportion of low apolipoprotein-A1 during the spread of the pandemic, including the peak and the recovery periods. Thanks to the power of the sample size, we simply compared graphically the temporal trends between years for the study. A significant difference of the mean daily levels of apolipoprotein-A1, and the daily proportion of low apolipoprotein-A1 was defined as an absence of overlap of the 95% confidence interval (95%CI) between the curves, during the same 34 weeks periods per year.

The second analysis was to exclude confounding factors. The same graphical method was used. In the large US cohort this method permitted also to describe the trends of apolipoprotein-A1 after a triple stratification on age, gender and the cause of liver disease.

The third primary endpoint was to assess the temporal association between the spread of Covid-19 and the decrease in apolipoprotein-A1. For this purpose, we compared graphically the daily mean number of confirmed Covid-19 cases and the daily proportion of low apolipoprotein-A1.

The diagnostic performance of biomarkers was assessed using non-parametric AUROCs. The prognostic values were assessed by survival curves, using Kaplan-Meier method, compared by Logrank test (cutoff being the median of this context of use) and adjusted by Cox model. The repeated serum assessments were compared by repeated ANOVA and Tukey-Kramer multiple-comparison test. R and NCSS-2020 were used as statistical software.

## Results

### Characteristics of patients included in serum-cohorts

The core temporal analysis compared the first 34 weeks (between January 1^st^ to August 20^th^, 2020) of consecutive anonymous serum 2020 *vs*. the serum 2019 from the three routinely followed cohorts. The number for USA was 212,297 serum in 2020, 266,976 serum in 2019, then for the French cohort 20,652 serum in 2020, 28,452 serum in 2019 and for the APHP-PSL cohort 3,122 serum in 2020, and 3,928 in 2019, described in Table 1. For the US-cohort in 2020, the median age was 54 years (IQR 41–63), 56% men, 80% followed for HCV and 20% for NAFLD, 71% with non-significant fibrosis (stage F0-F1 by FibroTest). During the same 34 weeks period in 2019 these characteristics were similar, 55 (IQR 42–63) years of age, 56% men, 80% HCV and 70% stage F0-F1.

**Table 1:** Characteristics of the patients included in US and French serum-cohorts.

Characteristics	2020	2019	P-Value
**US-cohort**	**N = 212,297**	**N = 266,976**	
Median age [IQR)	54.1 [40.5–63.4]	55.0 [41.6–63.3]	<0.001
Age category n (%)			<0.001
< 50 year	87,473 (41.2)	102,984 (38.6)	
50 to <70 year	102,797 (48.4)	138,026 (51.7)	
> = 70 year	22,027 (10.4)	25,966 (9.7)	
Male sex (%)	119,227 (56.2)	148,997 (55.8)	<0.001
Apolipoprotein-A1<1.25 g/L (%)	70,212 (33.1)	71,994 (27.0)	<0.001
**Median laboratory (IQR)**			
Apolipoprotein-A1 g/L	1.36 [1.18–1.57]	1.41 [1.23–1.62]	<0.001
Haptoglobin g/L	1.23 [0.82–1.68]	1.23 [0.82–1.68]	0.54
Alpha-2-macroglobulin g/L	2.15 [1.64–2.92]	2.26 [1.70–3.07]	<0.001
GGT IU/L	37 [21–78]	37 [21–79]	0.05
ALT IU/L^1^	38 [22–69]	38 [22–68]	<0.001
Total bilirubin micromol/L	6.84 [5.13–10.26]	6.84 [5.13–10.26]	0.002
Fibrosis stage by FibroTest (%)			<0.001
F0	108,925 (51.3)	132,698 (49.7)	
F1	42,667 (20.1)	53,605 (20.1)	
F2	15,467 (7.3)	20,289 (7.6)	
F3	20,847 (9.8)	27,823(10.4)	
F4	22,886 (10.8)	30,669 (11.5)	
Non interpretable	1,505 (0.7)	1,892 (0.7)	
NAFLD (surveillance by NASH-FibroTest)^2^	N = 44,049 (20.1)	N = 48,201 (18.1)	<0.001
Weight Kg	88.5 (74.8–104.8)	88.5 (74.8–104.3)	0.13
Aspartate amino transferase (AST) IU/L	34 (25–52)	34 (25–52)	0.92
Total cholesterol mmol/L	4.53 (3.78.5.33)	4.53 (3.80–5.28)	<0.001^3^
Triglycerides mmol/L	1.57 (1.13–2.23)	1.57 (1.13–2.23)	0.03
Fasting glucose mmol/L	5.72 (5.11–6.89)	5.61 (5.06–6.78)	<0.001
**French-cohort**	**N = 20,652**	**N = 28,452**	
Median age [IQR)	53.8 (41.0–64.1)	52.4 (39.5–63.1)	<0.001
Age category n (%)			<0.001
< 50 year	8,523 (41.3)	12,668 (44.5)	
50 to <70 year	9,223 (44.7)	12,291 (43.2)	
> = 70 year	2,906 (14.0)	3,493 (12.3)	
Male sex (%)	11,947 (57.8)	16,356 (57.5)	<0.001
Apolipoprotein-A1<1.25 g/L (%)	6,465 (31.3)	7,794 (27.4)	<0.001
**Median laboratory (IQR)**			
Apolipoprotein-A1 g/L	1.38 (1.20–1.58)	1.40 (1.23–1.61)	<0.001
Haptoglobin g/L	1.18 (0.79–1.62)	1.15 (0.77–1.57)	<0.001
Alpha-2-macroglobulin g/L	1.96 (1.52–2.60)	2.01 (1.56–2.69)	<0.001
GGT IU/L	37 (21–80)	34 (20–73)	<0.001
ALT IU/L	31 (21–51)	31 (21–50)	0.47
Total bilirubin micromol/L	8.7 (6–12)	8.7 (6–12)	0.22
Fibrosis stage by FibroTest (%)			
F0	10,650 (51.6)	14,887 (52.3)	<0.001
F1	4,592 (22.2)	6,239 (21.9)	
F2	1,492 (7.2)	2,045 (7.2)	
F3	1,859 (9.1)	2,477 (8.7)	
F4	1,886 (9.1)	2,579 (9.1)	
Non interpretable	190 (0.8)	201 (0.8)	
NAFLD (surveillance by NASH-FibroTest)^2^	2,904 (14.1)	3,559 (12.5)	<0.001
Weight Kg	83 (71–97)	83 (71–96)	0.18
Aspartate amino transferase (AST) IU/L	30 (23–42)	30 (23–41)	0.85
Total cholesterol mmol/L	4.78 (4.00–5.55)	4.76 (4.00–5.62)	0.24
Triglycerides mmol/L	1.38 (1.00–1.96)	1.40 (1.00–2.00)	0.02
Fasting glucose mmol/L	5.74 (5.14–6.96)	5.73 (5.17–6.89)	0.53
**APHP-PSL-cohort**	**N = 3,122**	**N = 3,928**	
Median age [IQR)	54.3 (41.7–65.0)	53.4 (40.8–62.9)	<0.001
Age category n (%)			<0.001
< 50 year	1282 (41.1)	1659 (42.2)	
50 to <70 year	1334 (42.7	1864 (47.5)	
> = 70 year	506 (16.2)	405 (10.3)	
Male sex (%)	1684 (53.9)	1964 (50.0)	<0.001
Apolipoprotein-A1<1.25 g/L (%)	1464 (47)	1176 (30)	
**Median laboratory (IQR)**			
Apolipoprotein-A1 g/L	1.27 (1.07–1.47)	1.40 (1.21–1.60)	<0.001
Haptoglobin g/L	1.34 (0.86–1.99)	1.24 (0.84–1.69)	<0.001
Alpha-2-macroglobulin g/L	1.67 (1.34–2.20)	1.78 (1.38–2.32)	<0.001
GGT IU/L	34 (21–67)	31 (20–57)	<0.001
ALT IU/L	28 (20–43)	27 (19–40)	0.55
Total bilirubin micromol/L	8 (6–11)	8 (5–11)	0.03
Fibrosis stage by FibroTest (%)			<0.001
F0	1873 (60.0)	2523 (64.2)	
F1	615 (19.7)	702 (17.9)	
F2	185 (5.9)	203 (5.2)	
F3	207 (6.6)	263 (6.7)	
F4	202 (6.5)	213 (5.4)	
Non interpretable	40 (1.3)	24 (0.6)	

^1^ Not normal distribution, the means of ALT were in 2020 61.5 IU/L vs 60.4 IU/L in 2019.

^2^ NASH-FibroTest included more components than FibroTest: weight, aspartate aminotransferase, total cholesterol, triglycerides, and fasting glucose.

^3^ Not normal distribution, the means of total cholesterol were in 2020 4.62 mmol/L vs in 2019 4.59 mmol/L. The P-value is 0.0003.

All the serum from 2018 were also analyzed for assessing the temporal variability of the biomarkers vs. 2019, two years without Covid-19, including for USA 407,138 serum in 2019, 383,865 serum in 2018, then for the French cohort 43,963 serum in 2019, 45,067 in 2018, and for the APHP-PSL cohort 6,119 in 2019, and 6,568 in 2018.

Due to the lockdown of populations during the pandemic, both in France and USA the number of the daily number of sera analyzed from January-August 20th, 2020 varied significantly compared to from January-August 20th, 2019 in the three cohorts of patients followed for a risk of liver fibrosis (S2C Fig).

### Decrease of apolipoprotein-A1 in serum-cohorts

**Fig 1:**
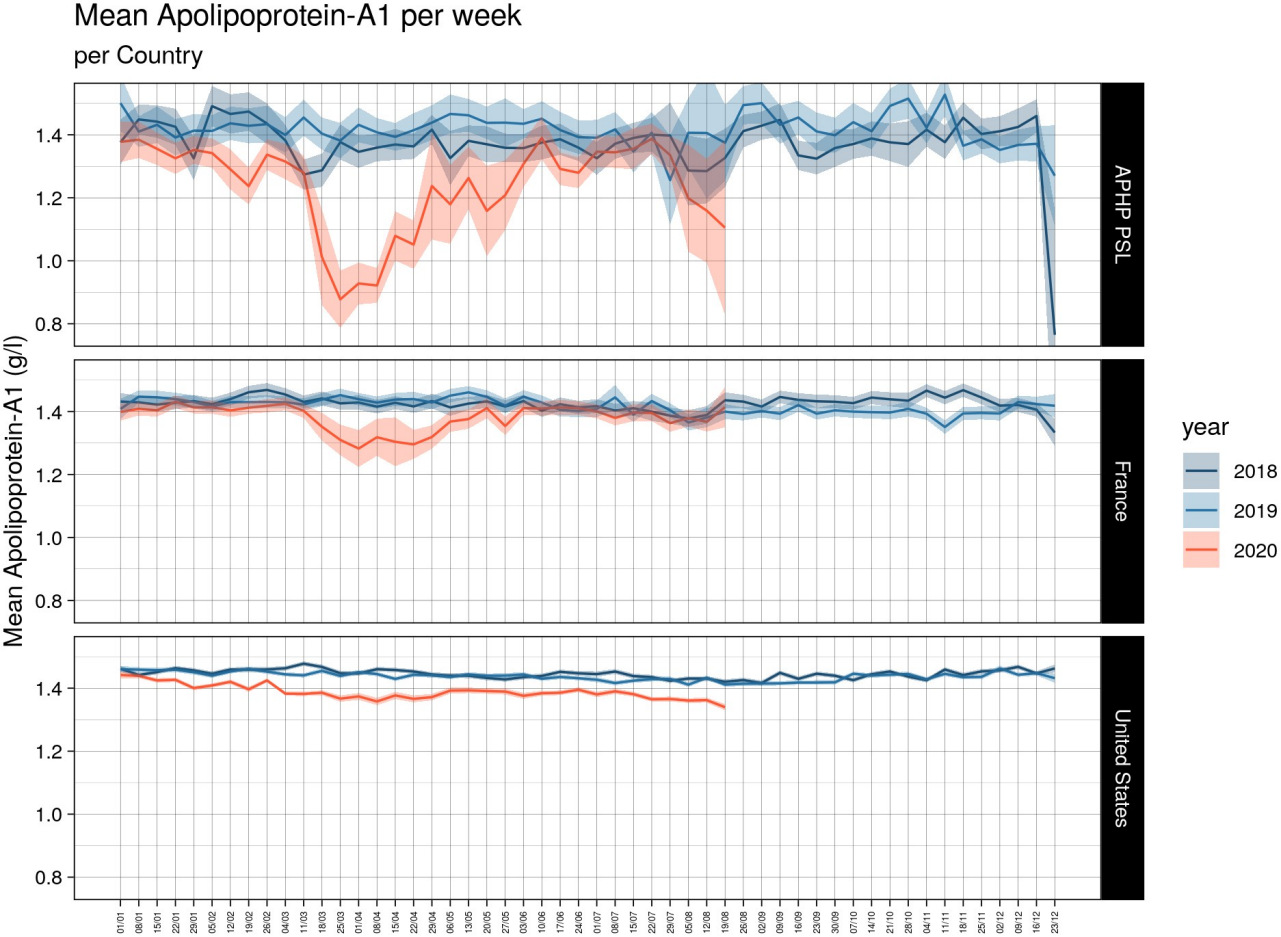
Quantitative decrease of apolipoprotein-A1 by cohort. Apolipoprotein-A1 decreased (P<0.001) in the three cohorts, starting early in January 2020 (red line with 95% confidence interval) in the US cohort (lower panel).

The reference daily proportion of low (<1.25 g/L) apolipoprotein-A1 assessed in the 2019 US-serum-cohort, was 27.3% (95% CI 27.2–27.5; 111,262 out of 407,138 serum). The mean daily levels of apolipoprotein-A1 decreased in the three serum-cohorts (Fig 1; all P<0.001), during the first 34 weeks of 2020. This was already highly significant in January 2020 in the US-cohort (lower panel), compared to first 34 weeks of 2019 and 2018. The mean daily proportion of serum with low apolipoprotein-A1 (<1.25 g/L) was 33.1% (95%CI 32.9–33.3 n = 70,212) on 34 weeks, that is 5.8% higher *vs*. the reference (P<0.001) in the same 34 weeks of 2019.

There was no significant difference between the years 2019 and 2018 for the daily mean by cohort (Fig 1) and only 0.8% difference between the proportion of low apolipoprotein-A1 assessed on the full 52 weeks 27.3% (95%CI 27.2–27.5; 111,262 out of 407,138) in 2019 vs. 26.5% (95%CI 26.3–26.6; 101,591 out of 383,865) in 2018.

**Fig 2:**
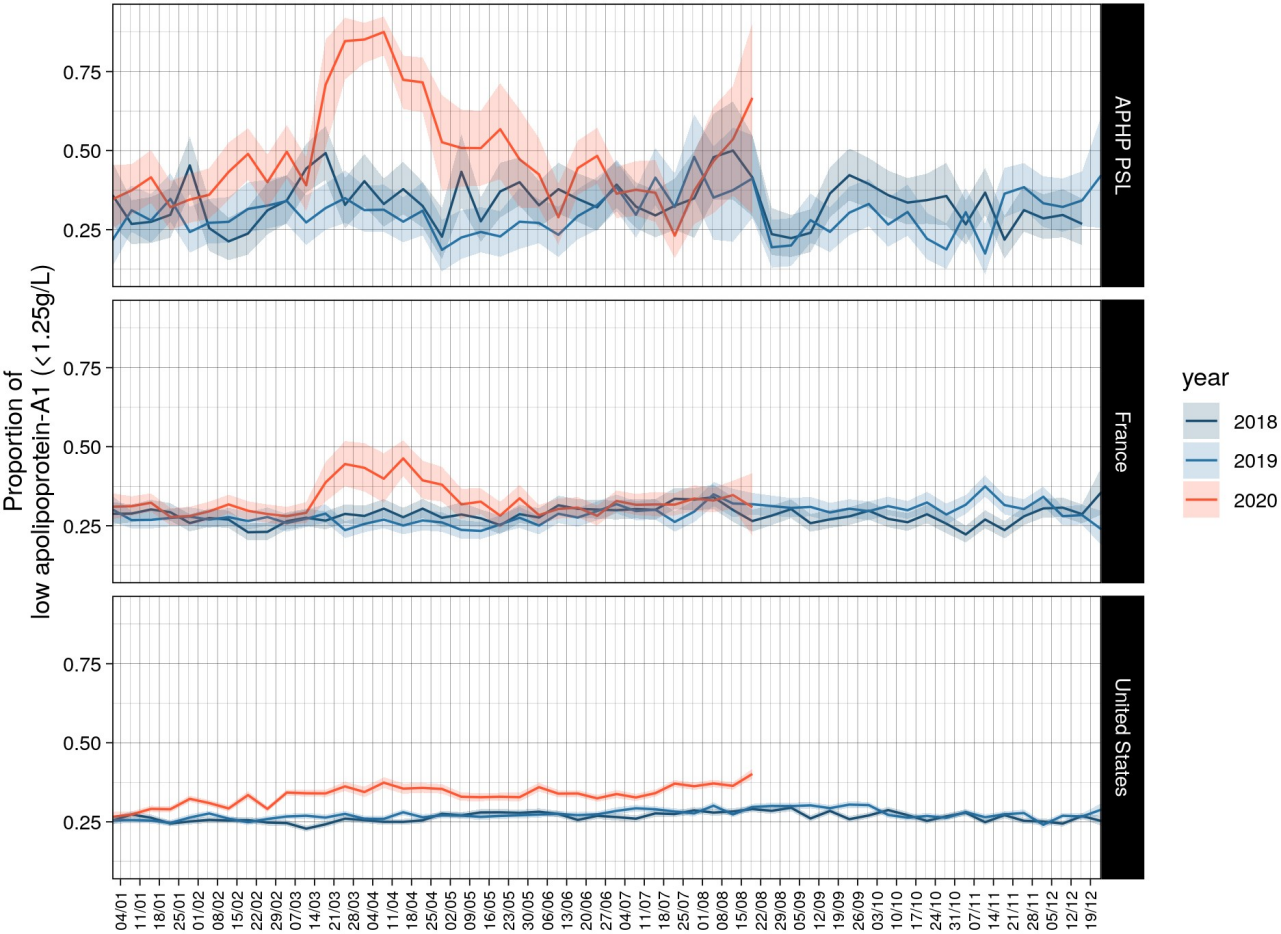
Proportion of serum with low apolipoprotein-A1 by cohorts. Low apolipoprotein-A1 was defined as below 1.25 g/L. Details in Table 2.

In the US cohort 2020, the proportion of low apolipoprotein-A1 was 37.0% on the first peak (April 7^th^), that is 9.7% greater than the reference proportion, 27.3%, in 2019. The proportion of low apolipoprotein-A1 was 40% on the second peak (August 18^th^), that is 10.7% greater than the reference proportion, 27.3%, in 2019. In France 2020, the increase reached 12.7% on April 14^th^ and dropped to 0% on June 20^th^ (Fig 2 and Table 2).

**Table 2:** Proportion of low apolipoprotein A1 among the three serum-cohorts.

Year	Cohort period	ApoA1 low	Total	Proportion % of low apolipoprotein-A1					
		<1.25g/L		34 weeks mean (95%CI)		First Covid-19 peak week			
		n	n	crude	standardized		crude	standardized	date of first peak
2018	APHP 52w	2,168	6,568	33.0 (31.9–34.2)	6.2		n.a.	n.a.	n.a.
2019	APHP 52w	1,812	6,119	29.6 (28.5–30.1)	2.3		n.a.	n.a.	n.a.
2019	APHP 34w	1,176	3,928	29.9 (28.5–31.4)	2.6		n.a.	n.a.	n.a.
2020	APHP 34w	1,464	3,122	46.9 (45.1–48.6)	19.6		80.0	52.7^2^	April 7^th^
2018	France 52w	12,691	51,635	28.2 (27.7–28.6)	1.4		n.a.	n.a.	n.a.
2019	France 52w	12,563	50,082	27.1 (26.5–27.6)	0.3		n.a.	n.a.	n.a.
2019	France 34w	7,794	28,452	27.4 (26.9–27.9)	0.1		n.a.	n.a.	n.a.
2020	France 34w	6,465	20,652	31.3 (30.4–31.8)	4.0		40.0	12.7^3^	April 14^th^
2018	USA 52w	101,591	383,865	26.5 (26.3–26.6)	-0.8		n.a.	n.a.	n.a.
**2019**	**USA 52w**^1^	**111,262**	**407,138**	**27.3 (27.2–27.5)**	**0**		**n.a.**	**n.a.**	**n.a.**
2019	USA 34w	71,994	266,976	27.0 (26.8–27.1)	-0.3		n.a.	n.a.	n.a.
2020	USA 34w	70,212	212,297	33.1 (32.9–33.3)	5.8		37.0	9.7^4^	April 7^th^

n.a.: not applicable. W: weeks. Standardized = (value- 27.3), the reference 2019 USA 52w value.

^1^ Used as the reference population for the baseline mean apolipoprotein value.

^2^ In the APHP-PSL cohort, the proportion of low apolipoprotein-A1 in 2020 was 80.0% on the first peak, that is 52.7% greater than the reference control proportion, 27.3%, in 2019.

^**3**^ In the French cohort, the proportion of low apolipoprotein-A1 in 2020 was 40.0% on the first peak, that is 12.7% greater than the reference control proportion, 27.3%, in 2019.

^**4**^ In the US cohort, the proportion of low apolipoprotein-A1 in 2020 was 37.0% on the first peak, that is 9.7% greater than the reference proportion, 27.3%, in 2019.

### Confounding factors in serum-cohorts

**Fig 3:**
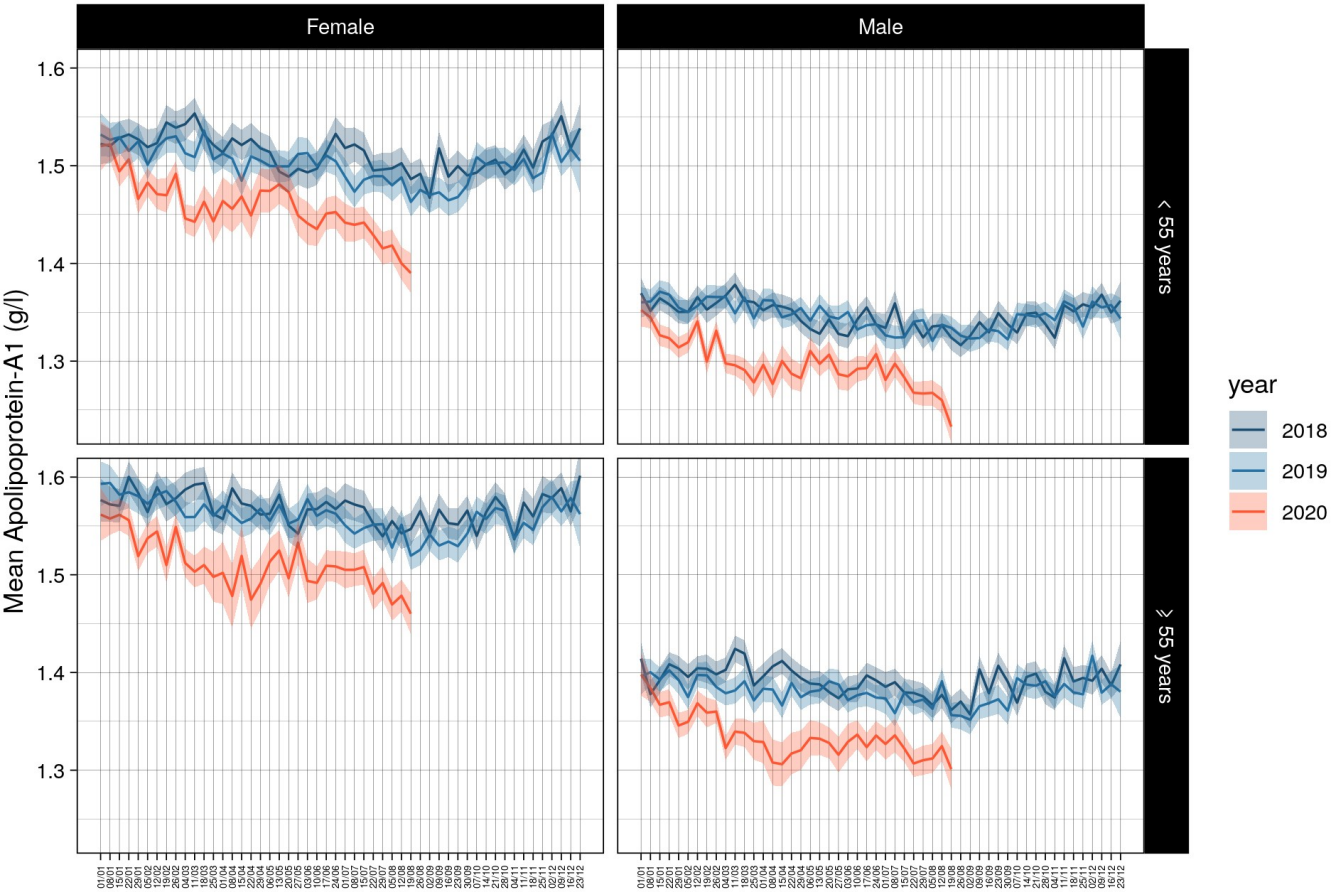
Decrease of apolipoprotein-A1 by gender and age in US cohort. The same significant kinetics were observed, P<0.001 between 2020 and previous years.

The same significant kinetics in apolipoprotein-A1 levels were observed after stratification of the temporal curves for gender and age in the three cohorts. The US-cohort was the only sample that had the necessary power to compare these two factors together between 2020 and 2019 and 2018, and in the subsets of patients with HCV or NAFLD (S3 Fig). The higher drops were observed in August 2020 for subjects younger than 55 years both for male and female (Fig 3).

**Fig 4:**
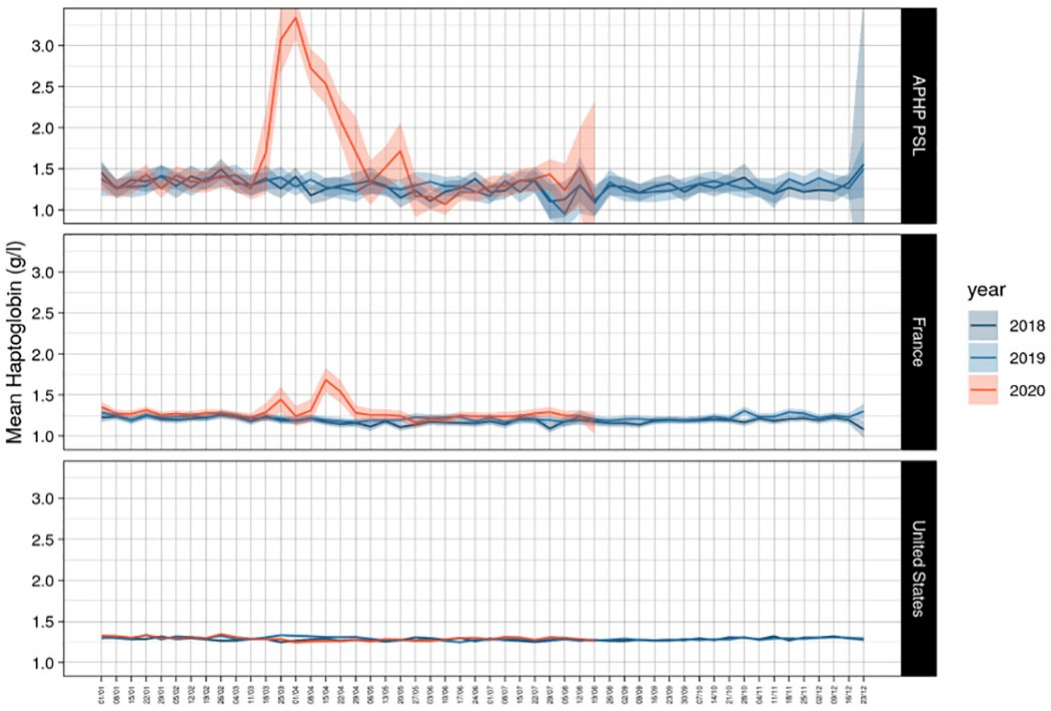
Absence of haptoglobin increase in the first 34 weeks of 2020 in the US cohort. Only the French cohorts at high risk of Covid-19 patients had a significant increase in haptoglobin.

Apolipoprotein-A1 decrease was similar during Covid-19 spread versus 2019 and 2018 in the US-cohort (S2 File, S3 Fig). The kinetics of apolipoprotein-A1 were not associated with those of haptoglobin in the US-cohort. As expected, there was a significant haptoglobin increase at the peak of the pandemic in the French cohorts and particularly in the APHP-PSL cohort (Fig 4) (S4 Fig).

For A2M, in the US-cohort, there was a significant lower mean serum value, when compared to 2019 and 2018, and detailed in S5 Fig. This significant decrease of A2M was regular since January 2018, persisted after stratification by age and gender, but was no longer significant in the serum of NAFLD, between 2020 and 2019 years. In HCV the significant decrease of A2M persisted only between the years 2019 vs 2018 after stratification by age and gender.

The other significant differences were, in the French-cohorts, GGT increased during the pandemic peak and returned to previous years’ value thereafter (S6 Fig), in the US-cohort and a transient increase in ALT in April 2020, (S7 Fig). No changes were observed for total bilirubin, total cholesterol, triglycerides, fasting glucose, weight or height between cohorts (S8 Fig).

### Temporal associations between apolipoprotein-A1 and spread of Covid-19 in serum-cohorts

**Fig 5:**
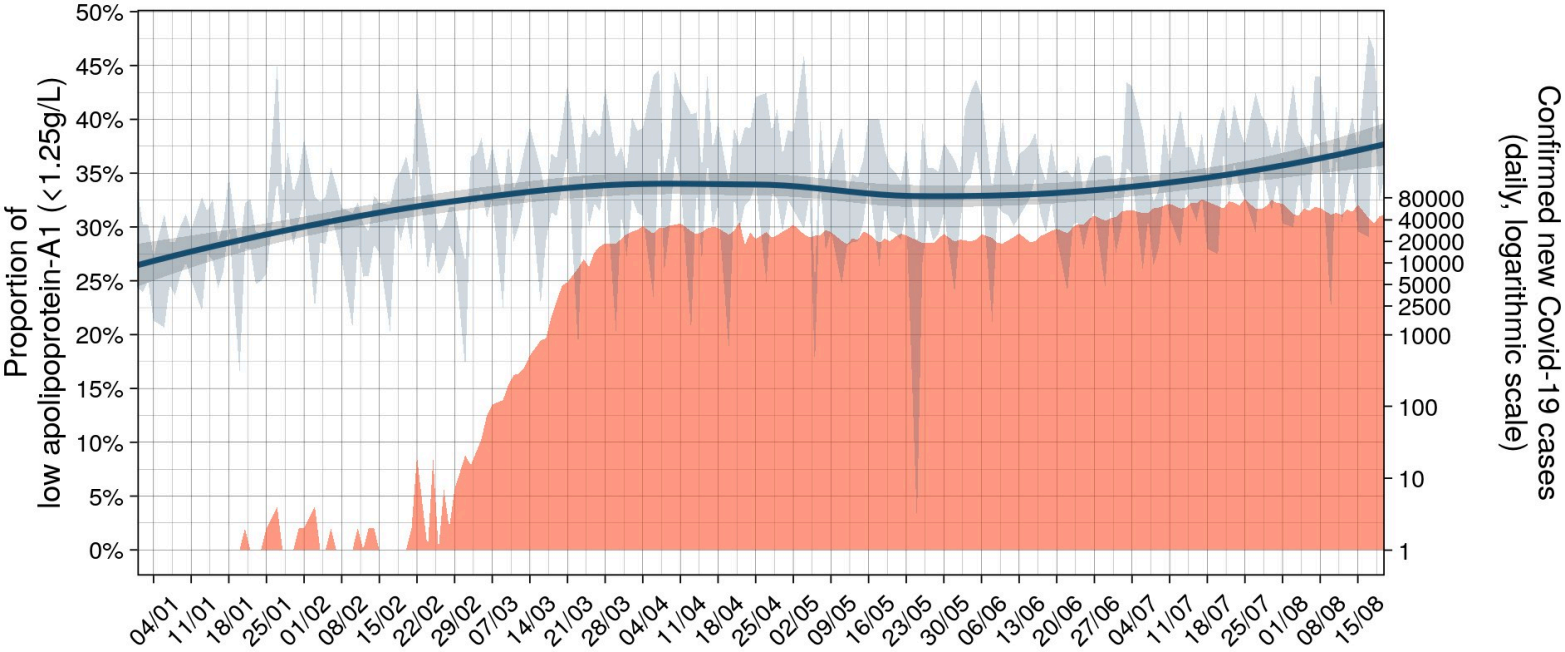
Number of confirmed Covid-19 cases per day and proportion of low (<1.25g/L) apolipoprotein-A1 in the US cohort during the first 34 weeks of 2020. The red graph is the number of confirmed cases per day in logarithmic scale. The black line is the daily mean proportion of low apolipoprotein-A1 (<1.25g/L; blue line;95%CI in grey).

The daily mean number of confirmed Covid-19 cases paralleled the daily proportion of low apolipoprotein-A1, about 10 days after. In USA, the first ten Covid-cases were declared mid-January 2020, when the proportion of low apolipoprotein-A1 had already increased by several percent. A plateau around 34% of low apolipoprotein-A1 was reached on March 21st that is two weeks before the plateau of confirmed Covid-19 daily cases around 40,000 reached on April 7th (Fig 5, Table 2).

In France, the first ten Covid-cases were declared first week of March 2020, when the proportion of low apolipoprotein-A1 started to increase by several percent (Fig 2). The first peak of cases (n = 7,000) was reached mid-April, as well as the first peak of low apolipoprotein-A1 (40.0%) on April 14^th^ (Fig 2). Details are given in S2A Fig.

### Characteristics of Covid-19 patients included in the sensitivity and prognostic assessment of apolipoprotein-A1 performances

A total of 136 consecutive patients with severe Covid-19, but who did not require ICU were included. Their characteristics were similar to those published in such severity profiles (Table 3, S3 File).

**Table 3:** Characteristics of Covid-19 patients of the prospective study.

Characteristics	COVID-19 diagnostic			
	PCR positive	PCR negative Adjudicated	P value	Total
Number n (%)	117 (100%)	19 (100%)		136
Median age (IQR) year	72 (57–82)	65.1 (49–74)	0.15	72 (59–83)
Age category			0.16	
<50 year	14 (12.0)	5 (26.4)		19 (14.0)
50 to <70 year	37 (31.6)	7 (36.8)		44 (32.3)
> = 70 year	66 (56.4)	7 (36.8)		73 (53.7)
Male sex	66 (56.4)	12 (63.2)	0.58	78 (57.4)
Geographic origin			0.53	
Caucasian	67 (57.3)	14 (73.7)		81 (59.6)
Subsaharan	17 (14.5)	1 (5.3)		18 (13.2)
North African, Middle East	20 (17.1)	2 (10.5)		22 (16.9)
Asian	13 (11.1)	2 (10.5)		15 (11.0)
Oxygen-support category			0.75	
Invasive oxygen support	0	0		0 (0–0)
Noninvasive oxygen support	82 (70.1)	14 (73.7)		96 (70.6)
None	35 (29.9)	5 (26.3)		40 (29.4)
Coexisting conditions				
Hypertension	65 (55.6)	9 (47.4)	0.51	74 (54.4)
Diabetes	32 (27.4)	3 (15.8)	0.29	35 (25.7)
Hyperlipidemia	32 (27.4)	7 (36.8)	0.40	39 (28.7)
Liver disease	11 (9.4)	4 (21.1)	0.13	15 (11.03)
Pulmonary disease	13 (11.6)	1 (5.6)	0.44	14 (10.8)
Severe disease associated	64 (55.2)	11(57.9)	0.82	75 (55.6)
Tobacco (ongoing or stopped)	35 (29.9)	8 (42.1.3)	0.29	43 (31.6)
Alcohol consumption	15 (12.9)	5 (26.3)	0.13	20 (14.8)
Initial presentation				
Anosmia (13 missing)	12 (11.1)	3 (20.0)	0.32	15 (12.2)
Ageusia (13 missing)	16 (14.8)	3 (20.0)	0.60	19 (15.5)
Headache (13 missing)	10 (9.3)	4 (26.7)	0.05	14 (11.4)
Dyspnea (12 missing)	59 (54.1)	8 (53.3)	0.95	67 (54.0)
Wheezing (12 missing)	3 (2.75)	0 (0.00)	0.52	3 (2.42)
Cough without spitting (12 missing)	47 (43.1)	6 (40.0)	0.82	53 (42.7)
Cough with spitting (12 missing)	18 (16.5)	2 (13.3)	0.75	20 (16.1)
Fatigue	61 (56.5)	8 (53.3)	0.82	69 (59.1)
Time clinic-inclusion (days)	8 (5–13.8)	11 (9–16.5)	0.13	9 (5–14)
Followup (days)	38 (27–53)	28 (26–36)	0.05	32 (27–49)
BMI (Kg/m^2^)	25 (23–29)	24 (22–28)	0.56	25 (23–28)
Apolipoprotein < = 1.25g/L	104 (88.9)	19 (100)	0.13	123 (90.4)
Median laboratory (IQR)				
Apolipoprotein-A1 g/liter	0.85 (0.73–1.06)	0.74 (0.61–0.87)	0.06	0.84 (0.70–1.03)
Haptoglobin g/liter	3.16 (2.20–4.19)	3.18 (1.59–3.86)	0.59	3.16 (2.22–4.08)
Alpha-2 macroglobulin g/liter	1.49 (1.24–2.06)	1.46 (1.12–2.01)	0.41	1.49 (1.22–2.05)
GGT IU per liter	47 (28–114)	58 (32–102)	0.27	49 (30–114)
ALT IU per liter	29 (20–47)	40 (15–57)	0.80	31 (20–51)
Total bilirubin micromol/L	7 (6–8)	9 (8–13)	0.01	8 (5–12)
Platelets 10^6^ per ml	211 (162–278)	279 (194–330)	0.06	221 (164–287)
Creatinine μmol per liter	78 (62–103)	78 (70–120)	0.97	78 (63–103)
Fasting glucose	6 (5.4–8.0)	6.6 (5.2–7.9)	0.38	6.1 (5.4–7.9)
Albumin g/L (18 missing)	31(27–33)	29 (27–39)	0.89	30 (27–33)
Procalcitonin (17 missing)	0.13 (0.08–0.27)	0.14 (0.09–0.23)	0.98	0.13 (0.09–0.27)
C-reactive protein (13 missing)	56 (20.3–95.6)	101 (15.4–252.9)	0.21	59 (20.3–102.3)
InterLeukin-6 (38 missing)	31 (13.5–55.2)	29 (8.05–119)	0.96	31 (12.3–55.5)
CPK (18 missing)	101 (51.5–243.3)	47.5 (37.5–127)	0.05	94 (45.8–233)
AST (11 missing)	44 (30–60)	34 (25–42)	0.04	41 (30–59)
LDH (16 missing)	347 (278–419)	353 (289–440)	0.68	347 (278–420)
D-dimer (24 missing)	1050 (550–2030)	1570 (1140–3980)	0.09	1125 (570–2158)
Troponin (14 missing)	16.4 (9.85–34.63)	17.2 (9.56–36.5)	0.99	16.4 (9.85–34.63)
Prothrombin time (16 missing)	92 (83–100)	82 (39–89)	0.005	90 (81–100)
White cells				
Neutrophil missing (14 missing)	4185 (2773–6145)	5945 (3963–7550)	0.05	4420 (2928–6200)
Eosinophil (12 missing)	0 (0–30)	10 (0–150)	0.14	0.05 (0–30)
Lymphocyte (14 missing)	965 (670–1235)	1150 (798–1700)	0.25	975 (685–1295)
Hemoglobin g/L (12 missing)	12 (11.2–13.6)	12 (10.5–14.7)	0.64	12 (11.1–13.8)
Treatment at risk of DILI				
Paracetamol oral (2–4 g/day)	43 (36.8)	5 (26.3)	0.38	48 (35.3)
Antibiotics	91 (77.8)	15 (78.9)	0.91	106 (77.9)
None	26 (22.2)	4 (21.1)		30 (22.1)
Without clavulinate	49 (41.9)	5 (26.3)		54 (39.7)
Whit clavulinate	68 (58.1)	14 (73.7)		82 (60.3)
Steroids	12 (10.3)	1 (5.3)	0.49	13 (9.56)
Hydroxy chloroquine	47 (40.1)	6 (31.6)	0.48	53 (39.0)
Total deaths at 4 weeks	14 (12.0)	2 (10.5)	0.86	16 (11.8)
No transfer to intensive care unit	98 (83.8)	18 (94.7)	0.21	116 (85.3)
No death	88 (75.3)	17 (89.5)		105 (77.2)
Death	10 (8.5)	1 (5.2)		11 (8.1)
Transfer to intensive care	19 (16.2)	1 (5.2)		20 (14.7)
No death	15 (12.8)	0 (0)		15 (11.0)
Death	4 (3.4)	1 (5.2)		5 (3.7)

### Specificity in general population: Specificity-cohorts

**Fig 6:**
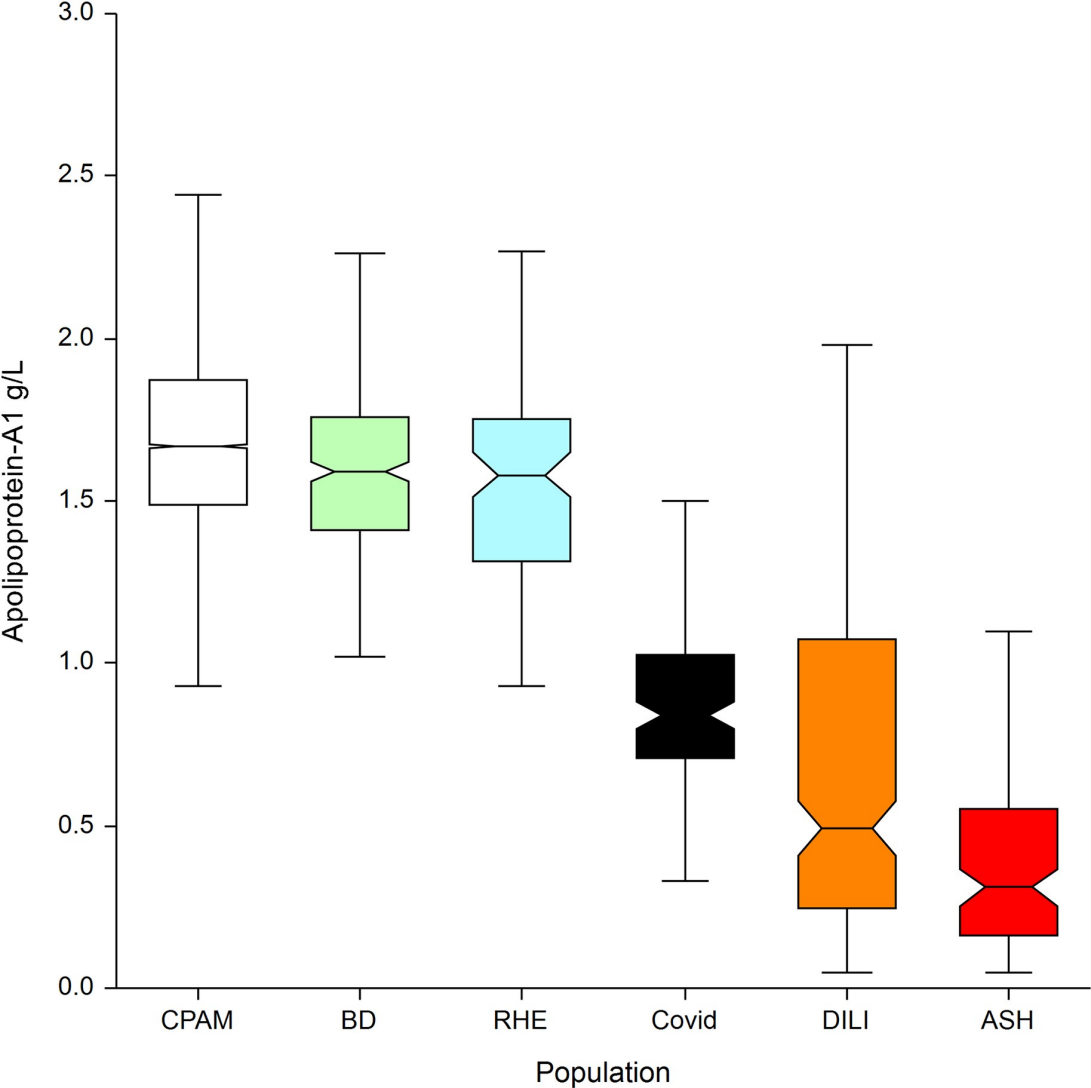
Apolipoprotein A1 median with IQR between the 6 populations. CPAM: general population assessing specificity of apolipoprotein-A1 in the sentinel context of use. Three populations were used for assessing higher risk of false positive: severe acute alcoholic hepatitis (ASH), drug induced liver disease (DILI), and rheumatologic disease (RHE). The blood donors population (BD) was at lower risk of false positive.

**Fig 7:**
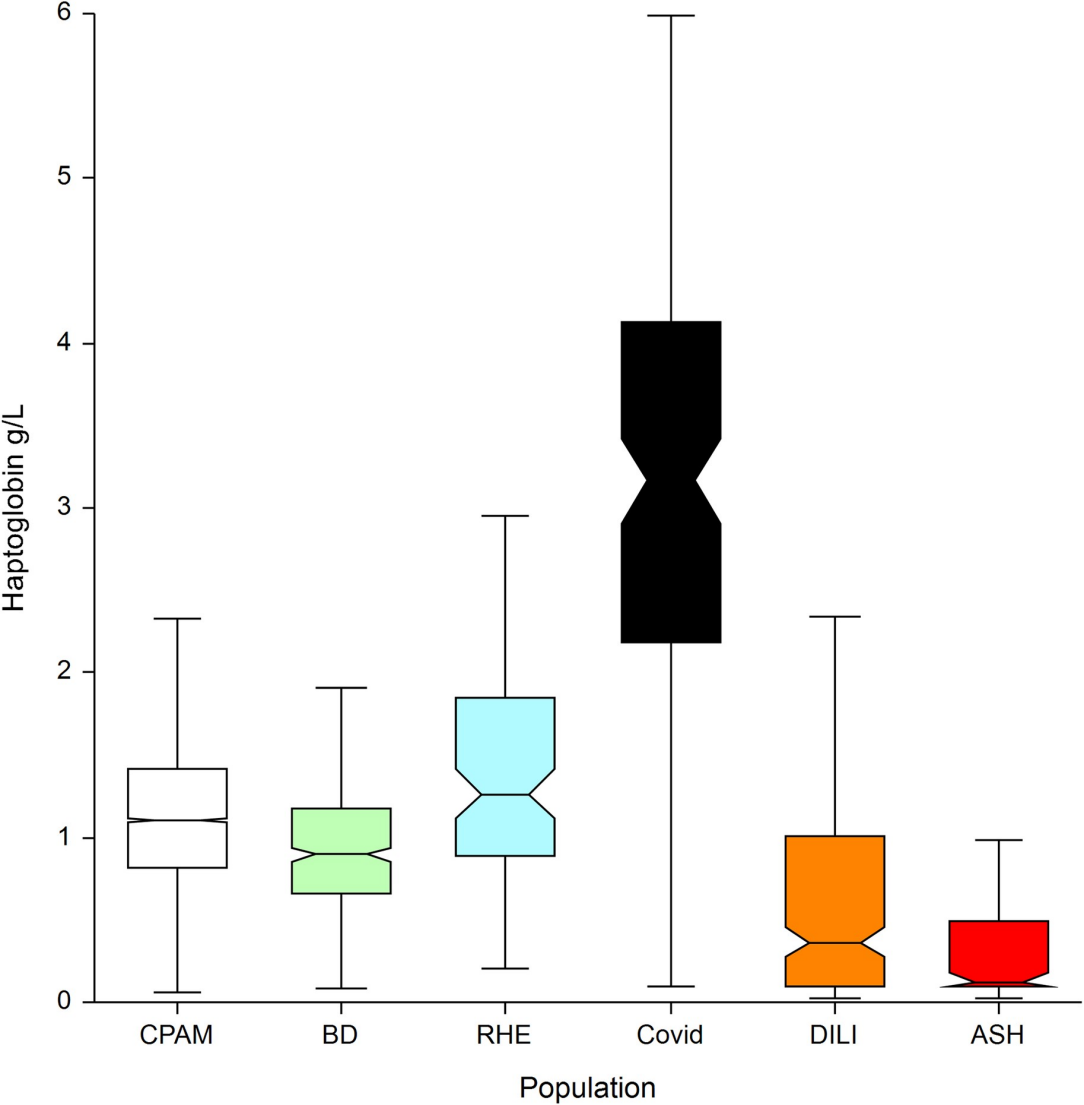
Haptoglobin median with IQR between the 6 populations.

The characteristics of patients included in the five specificity-cohorts are presented in Table 4, the median value of apolipoprotein at inclusion in Fig 6 and Table 5 and for haptoglobin in Fig 7 and Table 6.

**Table 4:** Baseline demographic and clinical characteristics of the 8,335 non-Covid-19 patients included for to test specificity in five “specificity cohorts” control groups.

Characteristics	General population (Core control group)	Blood donors	Rheumatologic patients	Drug-induced liver injury patients	Severe acute alcoholic hepatitis
Acronym	CPAM^1^	FibroFrance	SAFE-T^2^	SAFE-T	FibroFrance
Design	Observational	Observational	Observational	Observational	Observational
Country	France	France	France	European	France
Prospective inclusion	Yes	Yes	Yes	Yes	Yes
Years of inclusion	2006–2008	2005	2008–2009	2006–2015	2005–2011
Fresh Serum	Yes	Yes	Yes	Yes or stored^3^	Yes
Central laboratory	APHP-PSL^4^	APHP-PSL	APHP-PSL	APHP-PSL	APHP-PSL
Date of publication	2010	2019	2019	2017	2015
Reference	7	17	15	14–15	16
Number	7481	393	100	238	123
Male (%)	4128 (55.2)	222 (56.5)	37 (37.0)	138 (58.0)	92 (74.8)
Age year median (IQR)	58 (50–60)	33 (23–44)	49 (41–58)	54 (44–63)	56 (49–61)

^1^CPAM: Caisse Primaire Assurance Maladie.

^2^SAFET: Safer and Faster Evidence-Based Translation Consortium.

^3^Among 238 patients, tests were analyzed on fresh serum for 177 samples, and were assessed, were analyzed on frozen samples stored at minus 80 degree Celsius for less than 2 years for 61 samples.

^4^APHP-PSL: Assistance Publique Hopitaux de Paris Pitié-Salpêtrière.

**Table 5:** Apolipoprotein-A1 median with IQR between the 6 populations.

Population	Count	Median ApoA1 g/L	Dunnets’ test differences (P<0.05)
**CPAM: social security general population**^1^	**7,481**	**1.67**	**ASH, DILI, Covid, RHE, BD**
BD: blood donors^2^	393	1.59	ASH, DILI, Covid, CPAM
RHE: rheumatology patients^2^	100	1.58	ASH, DILI, Covid, CPAM
**Covid-19**^3^	**136**	**0.84**	**ASH, DILI, RHE, CPAM, BD**
DILI: drug induced liver disease^2^	238	0.49	ASH, Covid, RHE, CPAM, BD
ASH: severe acute alcoholic hepatitis^2^	123	0.31	DILI, Covid, RHE, CPAM, BD
Total	8,471	1.64	Not applicable

^**1**^Core population for assessing the specificity (1-% false positive) of apolipoprotein-A1 (ApoA1).

^**2**^Other population for assessing the specificity (1-% false positive) of apolipoprotein-A1 (ApoA1).

^**3**^Core population for assessing the sensitivity of apolipoprotein-A1 (ApoA1).

**Table 6:** Haptoglobin median with IQR between the 6 populations.

Population	Count	Median Hapto g/L	Dunnets’ test differences (P<0.05)
**CPAM: social security general population**^1^	**7,481**	**1.10**	**ASH, DILI, Covid, RHE, BD**
BD: blood donors^2^	393	0.90	ASH, DILI, Covid, RHE, CPAM
RHE: rheumatology patients^2^	100	1.27	ASH, DILI, Covid, BD, CPAM
**Covid-19**^3^	**136**	**3.17**	**ASH, DILI, RHE, CPAM, BD**
DILI: drug induced liver disease^2^	238	0.37	ASH, Covid, RHE, CPAM, BD
ASH: severe acute alcoholic hepatitis^2^	123	0.12	DILI, Covid, RHE, CPAM, BD
Total	8,471	1.08	Not applicable

^**1**^Core population for assessing the specificity (1-% false positive) of apolipoprotein-A1 (ApoA1).

^**2**^Other population for assessing the specificity (1-% false positive) of apolipoprotein-A1 (ApoA1).

^**3**^Core population for assessing the sensitivity of apolipoprotein-A1 (ApoA1).

**Fig 8:**
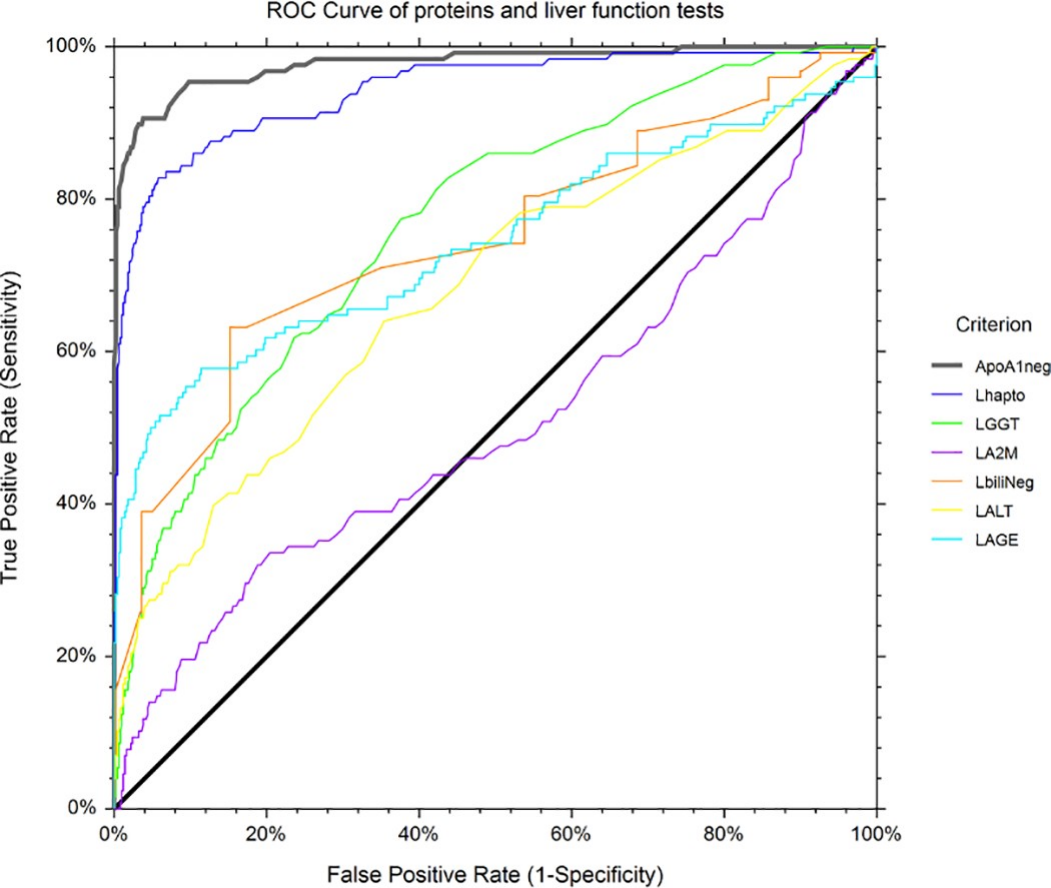
Area under the ROC curves (AUROC) of each FibroTest components.

The area under the characteristics curve (AUROC;95%CI) in 136 Covid-19 cases and 7,481 core controls was 0.979 (0.959–0.989), which outperformed haptoglobin and liver function tests (Fig 8 and Table 7). Apolipoprotein-A1 at a cutoff of 1.25 g/L, had the best Youden index (86.7%) with a sensitivity of 90.6% (84.2–95.1) and a specificity of 96.1% (95.7–96.6) for the diagnosis of Covid-19.

**Table 7:** Area under the ROC curves (AUROC) of each FibroTest components.

Criterion	Count	AUROC	Standard Error	P-Value vs ApoA1	Lower 95%CI	Upper 95%CI
ApoA1 negative	7617	0.979	0.007	NA	0.959	0.989
Haptoglobin	7617	0.938	0.014	0.007	0.903	0.961
GGT	7617	0.540	0.029	<0.001	0.481	0.595
A2M	7617	0.759	0.021	<0.001	0.715	0.796
Bilirubin negative	7617	0.712	0.028	<0.001	0.654	0.762
ALT	7617	0.637	0.028	<0.001	0.579	0.688
AGE	7617	0.760	0.028	<0.001	0.700	0.810

#### Primary diagnostic endpoint

136 Covid-19 cases and 7,481 healthy volunteers’ controls. Apolipoprotein-A1 at the 1.25 g/L cutoff, had a sensitivity of 90.6% (95%CI 84.2–95.1) and a specificity of 96.1% (95.7–96.6) for the diagnostic of Covid-19.

For a prevalence of 1.8% (136/7617;1.5–2.1) of Covid-19 cases, the positive predictive value was 30.0% (25.6–34.7) and the negative predictive value was 99.8% (99.7–99.9).

The adjusted predictive values according to prevalence predicted in the French population [16], were detailed in S2 File. The specificity-sensitivity including blood donors were detailed in S9A Fig, including patients with rheumatological disease in S9B Fig, and including all the integrated six databases in S9C Fig.

During the study period, in the same department 43 patients without suspected Covid-19 were excluded (S3 Table). These patients were admitted for mixed severe diseases during the pandemic, and therefore could not be used to assess the specificity of apolipoprotein-A1 in the context of use of an early detection test in the general population.

### Patients with diarrhea

The prevalence of diarrhea on initial presentation was 29 out of 131 cases (22.1%;95%CI 15.4–30.2). In this subset, the only significant difference was a lower median number of polynuclear leucocytes (S4 Table, S2 File).

### Prognostic values of apolipoprotein-A1 in Covid-19 patients

**Fig 9:**
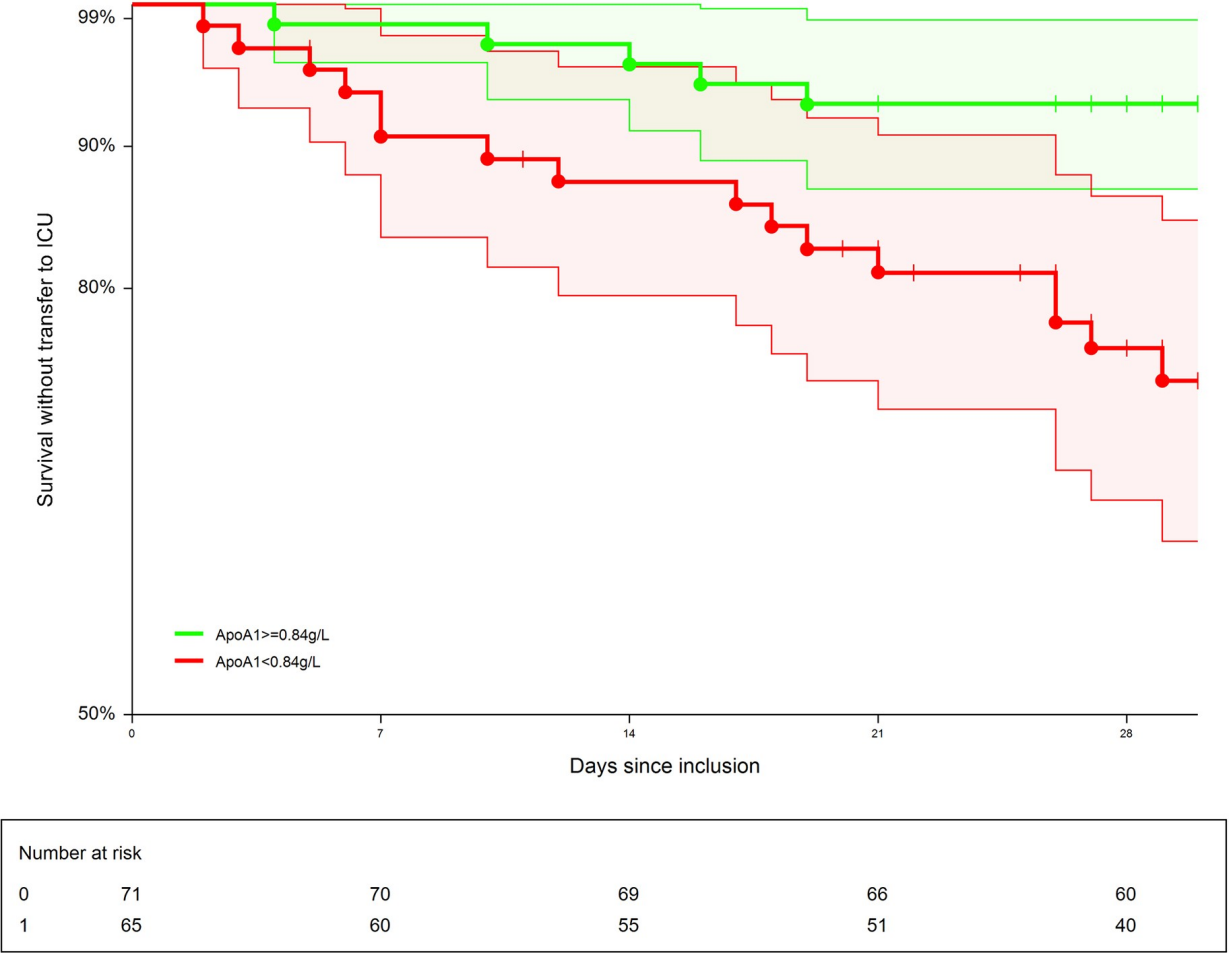
Survival without transfer to intensive care unit (ICU). The 71 patients with apolipoprotein-A1 value > = 0.84 g/L, the median value at inclusion, had a significant higher survival without ICU (93.0%;87.0–98.9) than the 65 patients with lower value (75.8%;65.1–86.5; P = 0.02).

The prognostic value of apolipoprotein-A1 at inclusion for predicting the primary outcome was significant, risk-ratio (RR;95%CI) = 5.61 (1.02–31.0; P = 0.04), adjusted on age (1.04;1.01–1.07; P = 0.04), GGT (2.88;1.01–8.19; P = 0.04). The 71 patients with apolipoprotein-A1 value> = 0.84 g/L, the median value at inclusion, had a significant higher survival without ICU (93.0%;87.0–98.9) than the 65 patients with lower value (75.8%; 65.1–86.5; P = 0.02) (Fig 9).

### Kinetics of apolipoprotein-A1 and haptoglobin in Covid-19 patients

**Fig 10:**
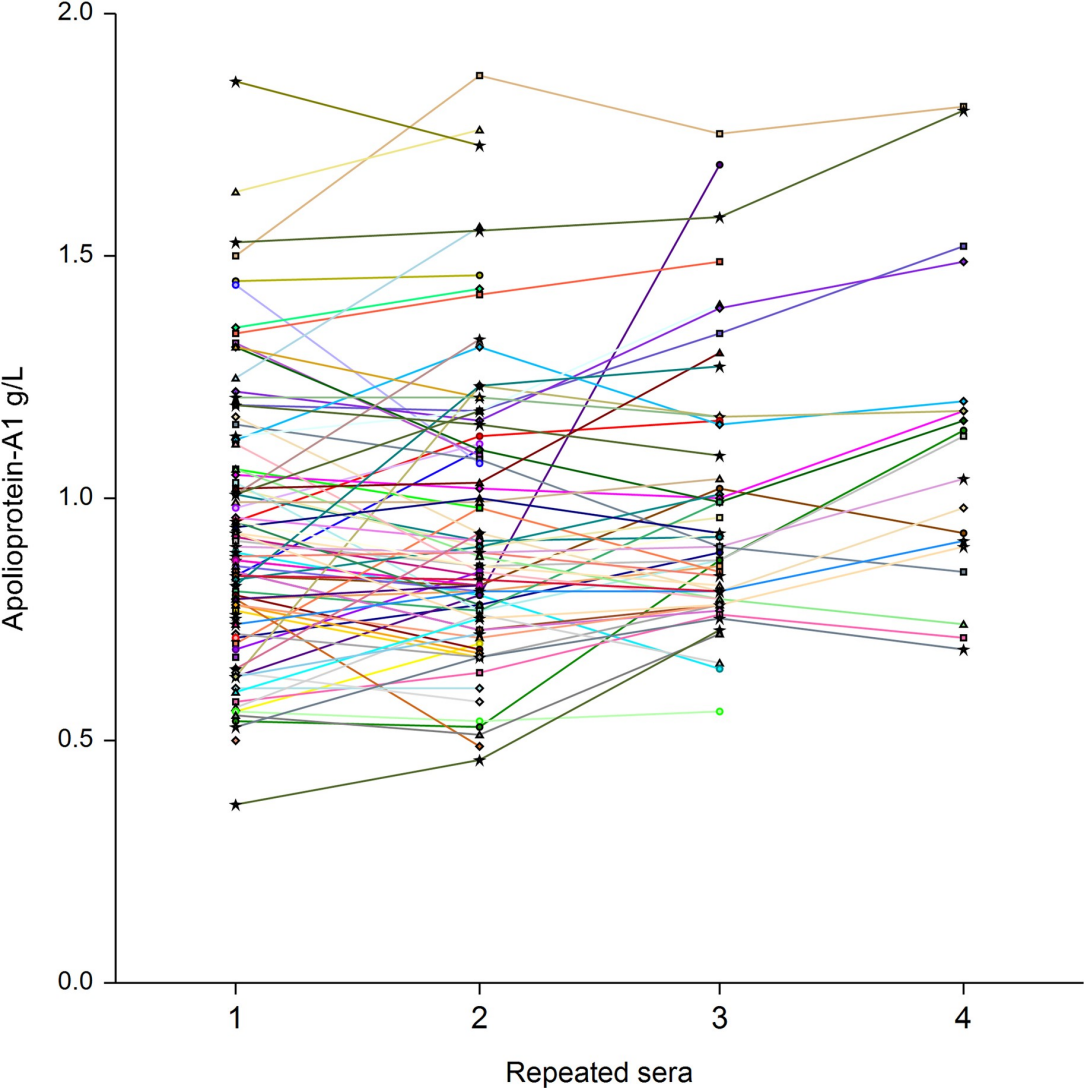
Serum apolipoprotein-A1 prospective 256 repeated measurements in 104 patients with Covid-19, who survived without transfer to intensive care unit. The median range interval between the first and the last serum was 11 days (IQR 8–16). Significant differences between ranks P<0.01 by Tukey-Kramer multiple-comparison test between first serum (0.92 g/L) vs third (0.98 g/L) and fourth sera (1.05).

**Fig 11:**
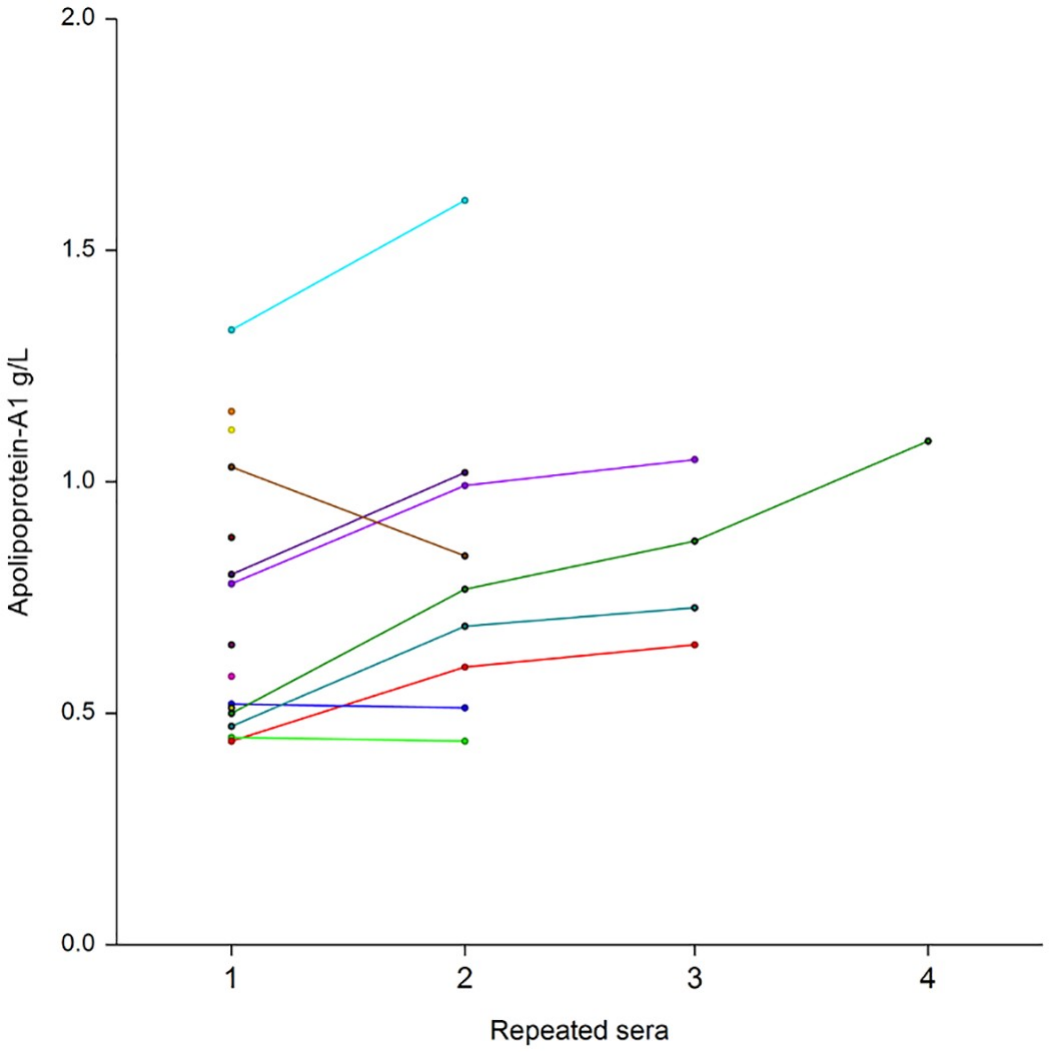
Serum apolipoprotein-A1 prospective 30 repeated measurements in 16 patients with Covid-19, who were transferred to intensive care unit and survived. The median interval between the four serum was 11 days (IQR 8–16). Significant differences between ranks P<0.01 by Tukey-Kramer multiple-comparison test between first sera (0.75 g/L) vs third (0.98 g/L) and fourth sera (1.25 g/L).

**Fig 12:**
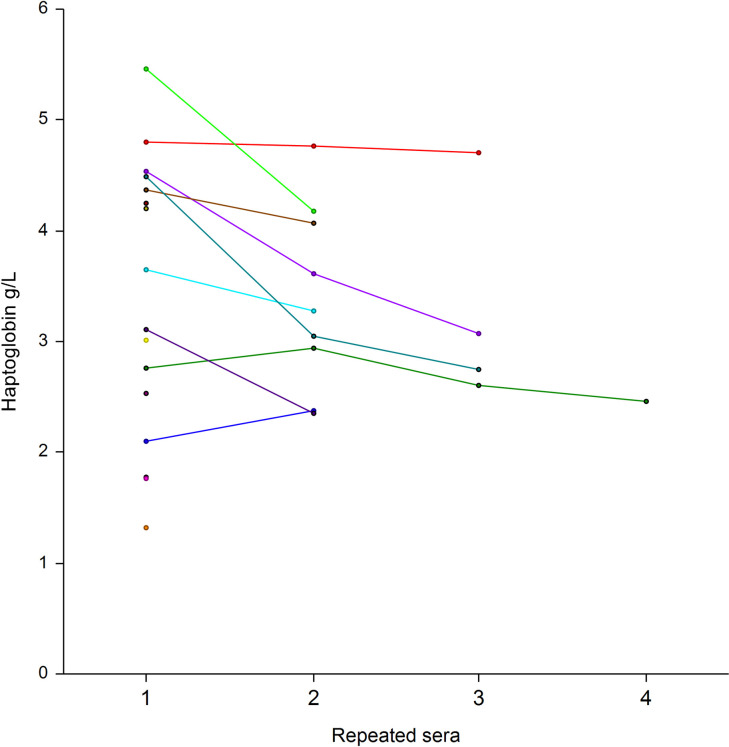
Serum haptoglobin prospective 256 repeated measurements in 104 patients with Covid-19, who survived without transfer to intensive care unit. The median range interval between the first and the last serum was 11 days (IQR 8–16). Significant difference between ranks P<0.01 by Tukey-Kramer multiple-comparison test between first sera (3.13 g/L) vs fourth sera (2.50 g/L.

**Fig 13:**
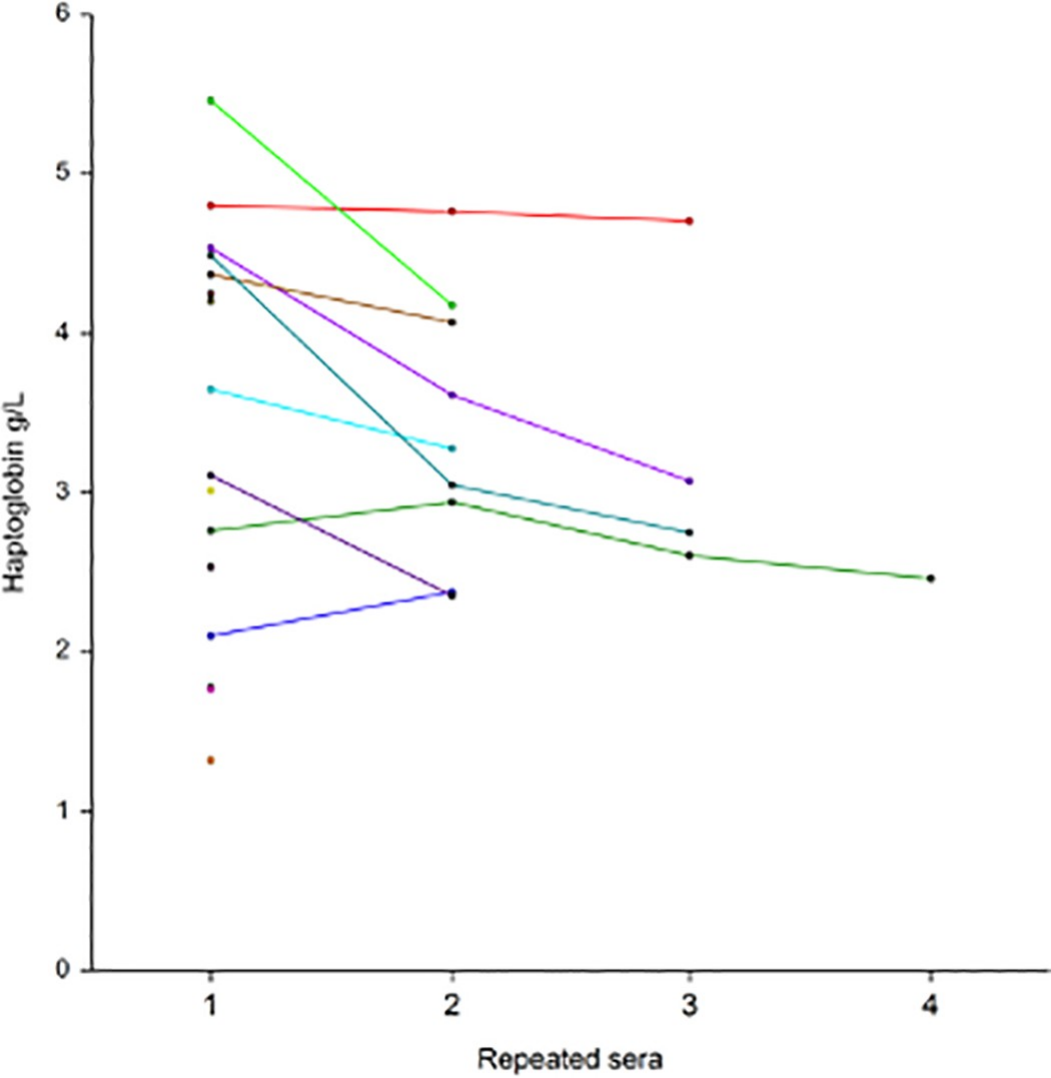
Serum haptoglobin 30 repeated measurements in 16 patients with Covid-19, who survived among those transferred to intensive care unit. The median range interval between the first and the last serum was 11 days (IQR 8–16). Significant differences between ranks P<0.01 by Tukey-Kramer multiple-comparison test between first sera (3.13 g/L) vs third sera (2.53 g/L).

Repeated assessments of 256 sera among 136 patients showed the low level of apolipoprotein-A1 at baseline, and thereafter the significant increase already at 10 days in patients who survived without ICU (Fig 10), as well as in the 16 patients who survived after their transfer to ICU (Fig 11). Similarly, the mean haptoglobin decreased also significantly in these survivors (Figs 12 and 13).

## Discussion

Our study shows that apolipoprotein-A1 displayed a highly significant decrease in 2020 vs previous years, and a highly significant negative daily time-related association with the number of Covid-19 cases. Apolipoprotein-A1 decrease had a high sensitivity in prospective hospitalized patients, with a high specificity in retrospective controls, and an independent prognostic value for the survival without transfer to ICU. These results have certain strengths and limitations (Table 8).

**Table 8:** Summary of the characteristics, interest and limitations of core populations.

Characteristics	2020 US possibly exposed to SARS-Cov2	Controls not exposed to SARS-Cov2	Covid-19 patients
Acronym	US serum-cohort	**CPAM**	ProCop EIT health
**Number**	**N = 212,297**	**N = 7,481**	**N = 136**
Context of use	Liver fibrosis surveillance	General French population 45 years of age and older	
Design	Serum consecutive database	Prospective study published in 2010	Prospective contemporaneous study
Test assessment	Fresh serum US laboratories	Fresh serum Centralized reference APHP-PSL laboratory	Fresh serum Centralized reference APHP-PSL laboratory
Statistical interest	Large ongoing surveillance	Specificity of test, lowest risk of bias than severe controls	Sensitivity and prognostic values of test, before ICU
Limitations	Not representative of US population, no SARS-Cov2 direct marker	Not contemporaneous	Small sample size but no asymptomatic carrier
Median age (IQR)	54.1 (40.5–63.4)	58 (50–60))	72 (59–83)
Age category n (%)			
< 50 year	87,473 (41.2)	1,864 (24.9)	19 (14.0)
50 to <70 year	102,797 (48.4)	5,159 (69.0)	44 (32.3)
> = 70 year	22,027 (10.4)	458 (6.1)	73 (53.7)
Male sex (%)	119,227 (56.2)	4,128 (55.2)	78 (57.4)
**Apolipoprotein-A1<1.25g/L (%)**	**70,212 (33.1;32.9–33.3)**	**263 (3.5;3.1–4.0)**	**123 (90.4;84.2–94.8)**
**Median laboratory (IQR)**			
Apolipoprotein-A1 g/L	1.36 (1.18–1.57)	1.67 (1.49–1.87)	0.84 (0.70–1.03)
Haptoglobin g/L	1.23 (0.82–1.68)	1.10 (0.81–1.42)	3.16 (2.22–4.08)
Alpha-2-macroglobulin g/L	2.15 (1.64–2.92)	1.46 (1.24–1.74)	1.49 (1.22–2.05)
GGT IU/L	37 (21–78)	24 (17–38)	49 (30–114)
ALT IU/L	38 (22–69)	23 (18–32)	31 (20–51)
Total bilirubin micromol/L	6.84 (5.13–10.26)	10 (9–14)	8 (5–12)
Fibrosis stage by FibroTest (%)			
F0 < = 0.27	108,925(51.3;51.1–51.5)	6521 (87.2;86.4–87.9)	66 (48.5;39.9–57.2)
F1 < = 0.48	42,667 (20.1;19.9–20.3)	810 (10.8;10.1–11.6)	36 (26.5;19.0-.34.7)
F2 < = 0.58	15,467 (7.3;7.2–7.4)	78 (1.0;0.8–1.3)	15 (11.0;6.3–17.5)
F3 < = 0.68	20,847 (9.8;9.7–9.9)	48 (0.6;0.5–0.8)	13 (9.6;5.2–15.8)
F4 < = 0.74	22,886 (10.8;10.6–10.9)	24 (0.3;0.2–0.5)	6 (4.4;1.6–9.4)
Non interpretable	1,505 (0.7;0.7–0.7)	0 (0.0)	0 (0.0)
Missing	0 (0.0)	0 (0.0)	0 (0.0)

### Decrease of apolipoprotein-A1 in serum-cohorts

The decrease in apolipoprotein-A1 during the peak of the pandemic in April was not so surprising, as very low levels of HDL-cholesterol were already known in severe pneumonia since 1920 (S1 Table) [2]. More intriguing was the very early decrease observed since January 2020 in the USA when the number of Covid-19 cases was unknown. The first known Covid-19 patient was detected on 27/12/2019 and 19/01/2020 in France and the USA, respectively (S3 File). The larger sample size of the US surveillance population, compared to the French, allowed detection of a significant 1% increase in the proportion of subjects possibly infected using the 1.25 g/L cutoff in January (Fig 2), without any inflammatory signal as assessed by haptoglobin. We hypothesized that the SARS-CoV2 virus influenced the liver or intestinal synthesis of apolipoprotein-A1, in asymptomatic patients or in those with unusual mild symptoms.

Indeed, there was a highly significant decrease in ApoA1 parallel (2 weeks before) to the confirmed Covid-19 cases. This decrease started few weeks before the incidence of confirmed cases, suggesting that apolipoprotein-A1 detected infected cases. Furthermore, the liver function biomarkers as well as the haptoglobin did not change so early.

### Confounding factors in serum-cohorts

The decrease in apolipoprotein-A1 in 2020 *vs*. previous years, as well as the time-related association of apolipoprotein-A1 in 2020 and Covid-19 might be due to numerous confounding factors. We acknowledge that consecutive sera were analyzed anonymously and therefore there was an unknown percentage of duplicated subjects with repeated sample over time. Since the database is anonymous, we don’t know how frequently individuals were measured, as well as the average sample per person in a year. However, the routine of surveillance is FibroTest between 12 months to 4 years according to the initial stages. This reduced the risk of repeated samples from the same person. In the context of the pandemic we used cohorts of subjects requiring surveillance of liver fibrosis biomarkers which represent at least 30% of the general adult population in the USA and in France. In these cohorts 70% of the subjects had no or minimal fibrosis. The decrease in apolipoprotein-A1 in 2020 compared to 2019, and 2018 cannot be explained by bias due to gender, age, the cause of liver disease (Table 1, S3 File), or the severity of liver diseases (S4 File). Despite the significant differences in several characteristics, none was associated with the decrease of apoplipoprotein-A1 in 2020 *vs*. 2019. The changes were observed both in HCV and NAFLD, after stratification on age and gender (S3 File). The prevalence of severe cases cannot explain the significant decrease in apolipoprotein-A1 already observed in January 2020. GGT a very sensitive liver biomarkers did not change during the first 3 months (S6 Fig). Finally, in these severe liver diseases, haptoglobin should also be significantly decreased, which was not the case (Fig 4).

In the US-cohort the proportion of serum with NAFLD was increased by 2% in 2020 (20.0%) vs 2019 (18.1% Table 1). However, after stratification for age and gender, no significant changes were observed for all other biomarkers (S8 Fig). ALT was the only biomarker of the liver tests which increased significantly at the 13^th^ week of 2020, above the usual mean value observed in 2019 (S7 Fig). This increase in ALT was not associated with any other changes (S8 Fig). We have no clear explanation. We hypothesize that another confounding factor could be a non-severe DILI, including oral acetaminophen or hydroxychloroquine misuse during the pandemic. Such factor could explain an increase in ALT, without increase in haptoglobin, in subjects with mild symptoms.

The absence of haptoglobin change (Fig 4) associated with the linear apolipoprotein-A1 decrease (Fig 1), has never been described before. It was known that in patients with severe fibrosis these two proteins decrease [10]. It was also known that in severe pneumonia the haptoglobin increase was associated to the apolipoprotein-A1 decrease (S1 Table), as we observed in the cohorts with high prevalence of severe Covid-19. The 34 weeks followup permitted to see the return to normal values of haptoglobin in these cohorts, associated with the decrease of severe Covid-19 cases admissions (Fig 4). Furthermore, the recovering patients followed by repeated serum in the prospective study, had both a significant increase of apolipoprotein-A1 (Figs 10 and 11) and a significant decrease in haptoglobin 10 days after inclusion (Figs 12 and 13).

### Temporal associations between apolipoprotein-A1 and spread of Covid-19 in serum-cohorts

Our results (Table 2, standardized proportion of low apolipoprotein-A1) suggest that the spread of the pandemic in the US-cohort would be around 5.8%, and 4.0% in the French-cohort. In France, this estimate does not differ from the recent French model that predicted a rate of infection between 2.8% to 7.2% in the general population [18].

This temporal association of low apolipoprotein-A1 with the number of confirmed cases, persisted in the two different pandemic changes. In France, after the national lockdown the proportion of low apolipoprotein-A1 returned to the cohort usual 2018–2019 values in June together with the dramatic regression of confirmed cases, still maintained in July. In the US, the apolipoprotein A1 and confirmed cases had the same kinetics with the dramatic increase in April and a plateau in June, still ongoing in August 2020 (Fig 5).

### Sensitivity of apolipoprotein-A1 in Covid-19 patients

There is a high risk of overestimating both the sensitivity and specificity of a test in Covid-19 when the participants enrolled in the studies might not be representative of targeted populations [1]. It was difficult to confirm the sensitivity for asymptomatic infection, due to the absence of validations of SARS-CoV2-antibodies. To validate the sensitivity of apolipoprotein-A1, a large number of asymptomatic “apparently healthy” subjects who are positive for SARS-CoV-2 viral nucleic acid testing is needed. Our patients included with Covid-19 symptoms had a median of 70 years of age and were severe enough to justify admission to hospital, but none of them required mechanical ventilation at admission, 86% survived, only 14.7% were transferred to the intensive care unit, and 11.8% died (Table 3). The sensitivity in the prospective part of our study was similar in the 19 patients who were negative for viral nucleic acid testing (94.7%) to that in positive patients (89.9%). Thus, a simple measurement of apolipoprotein-A1 could be useful for clinicians due to the high percentage of false negatives in available viral nucleic acid testing [19]. Apolipoprotein-A1 or HDL-cholesterol are already being assessed in many large ongoing studies in patients with chronic diseases, which could rapidly validate our results.

### Specificity in general population: Specificity-cohorts

Our previous cohorts of patients with severe liver diseases allowed us to identify the major risks of a significant decrease in apolipoprotein-A1 (false positives), mainly due to severe hepatic insufficiency and severe fibrosis. An impact of malnutrition on apolipoprotein-A1 values, was excluded as no significant changes in weight were observed. The controls not-exposed to SARS-Cov2 in the general population (CPAM) was prospective, but with possible limitations as not being contemporaneous (Table 8).

### Prognostic values of apolipoprotein-A1 in Covid-19 patients

The univariate prognostic value of apolipoprotein-A1 was confirmed (Fig 9) but to our knowledge, it was the first time that its prognostic value persisted after adjustment by age, a marker of acute inflammation (haptoglobin), a sensitive marker of liver injury (GGT), and a marker of liver fibrosis (A2M).

### Temporal association in Covid-19 patients

Among patients with recovery, there was a significant increase of apolipoprotein-A1 10 days after admission. Therefore, apolipoprotein-A1 measurement could help for the decision to transfer to ICU independently of other prognostic factors such as age and haptoglobin.

Furthermore, this kinetics of apolipoprotein-A1, a decrease during the hospitalization and an increase to usual reference values during recovery of Covid-19 cases is a strong argument for a causal interpretation of the temporal association observed also prospectively in the French cohort. The ongoing followup in the US cohort could permit to validate this normalization of apolipoprotein-A1.

### Similarly, the mean haptoglobin decreased also significantly in these survivors

#### Mechanisms of the early decrease in apolipoprotein-A1 before recognition of the pandemic

We never observed such profile of biomarkers in our experience since 2001 with more than three million of FibroTest assessed in liver diseases (S1 File) [10, 12–17].

Although several mechanisms explaining the decrease in apolipoprotein-A1 in late severe Covid-19 pneumonia are known (S1 Table), the reason for the early decrease before the acute phase, when haptoglobin remained normal is unclear. In patients with severe pneumonia, apolipoprotein-A1 decrease was associated with acute inflammation and the “cytokine storm” with an increase in IL6 and acute phase proteins such as CRP and haptoglobin. This dissociation suggests that different mechanisms play a role in the early influence of the SARS-CoV2 virus on the synthesis of apolipoprotein-A1, and the intestine could be more involved than the liver.

The SARS-CoV2 virus could impact several pathways leading to a decrease in the intestinal synthesis and absorption of apolipoprotein-A1 in the small intestine resulting in the decrease in serum [3–9]. These mechanisms are discussed in S1 File. The first could be the inhibition of lysophosphatidylcholine-acyltransferase-3 activity. There is evidence of direct SARS-CoV2 infection of the endothelial cell and diffuse endothelial inflammation in the intestine [7–9]. SARS-CoV2 uses angiotensin-converting enzyme-2 receptor (ACE-2) expressed on endothelial cells, to infect the host, widely expressed in the lung, intestine and liver [9, 20]. The second mechanism could be an impact of the virus through the intestinal mucus [21]. Apolipoprotein-A1 is released as a free apolipoprotein from the apical side of enterocytes into the lumen in the fasting state. Apolipoprotein-A1 had faster turnover in mucus, which could be a target for SARS-CoV2 [7, 8, 21].

## Conclusion

Despite the limitations of this study, these results suggest that apolipoprotein-A1 could be a component of multi-analyte Covid-19 population markers, but also diagnostic tests in individual patients at risk, and prognostic tests in individual Covid-19 patients. This ubiquitary protein seems useful in less symptomatic carriers of SARS-CoV2 as very early biomarker but also in patients with severe Covid-19 patients as an independent prognostic marker. It could also help to manage patients with a clinical suspicion of Covid-19 and a negative virological test. These results must be validated in independent cohorts, ideally with virologic and efficiency endpoints. The role of SARS-CoV2 in the possible “asymptomatic” decrease in apoliprotein-A1 could be related to intestinal infection without or before overt pulmonary disease. Finally, one hundred years after surrogate sentinel HDL-cholesterol for pneumonia [2], apolipoprotein-A1 for the second time in a century, could be “one of the early warning systems that alert the world to potential outbreaks” [22].

## Supporting information

S1 File Mechanisms of the early decrease in apolipoprotein-A1 before recognition of the pandemic, liver or intestine. (DOCX)

S2 File Methods. (DOCX)

S3 File Results. (DOCX)

S4 File Discussion. (DOCX)

S1 Table Review of the articles validating the prognostic value of apolipoprotein-A1, haptoglobin, and HDL-cholesterol for overall mortality, in severe sepsis and liver diseases. (DOCX)

S2 Table Comorbidity with liver disease and liver dysfunction in patients with Covid-19. (DOCX)

S3 Table Baseline demographic and clinical characteristics of the 43 patients pre-included during the same period, who were not considered to have Covid-19 by consensus, and not included in the prospective observational study. (DOCX)

S4 Table Baseline demographic and clinical characteristics of the patients Covid-19 included in the prospective observational study, according to initial diarrhea. (DOCX)

S1 Fig Prognostic value of HDL cholesterol, a surrogate of apolipoprotein-A1. A. Prognostic value of HDL cholesterol, a surrogate of apolipoprotein-A1 (meta-analysis from Liu 2020). B. Prognostic value of HDL cholesterol, a surrogate of apolipoprotein-A1 (from Cirstea 2017).
(DOCX)

S2 Fig Daily number of confirmed Covid-19 cases between January 1^st^ to August 20^th^, 2020. A. Number of confirmed covid-19 cases in France. B. Number of confirmed covid-19 cases in USA. C. Variability in the daily number of sera analyzed from January-August 20th 2020 (red bars and line with 95% confidence interval) compared to from January-August 20th 2019 (blue) in the APHP-PSL hospital, (upper panel), in the French cohort (upper panel) and in the US cohort (lower panel). The variability is expressed as the processing of tests (ratio between daily numbers) in 2020 vs 2019.
(DOCX)

S3 Fig Serum apolipoprotein ApoA1 variability. A. Serum apolipoprotein ApoA1 variability during covid-19 spread versus the same days in 2019–2018 in the APHP-PSL hospital, French and US cohorts, by gender. B. Serum apolipoprotein ApoA1 variability during covid-19 spread versus the same days in 2019–2018 in the APHP-PSL hospital, French and US cohorts, by age. C. Serum apolipoprotein ApoA1 variability during covid-19 spread versus the same days in 2019–18 in the US cohort, by age and gender, in patients with chronic hepatitis C. D. Serum apolipoprotein ApoA1 variability during covid-19 spread versus the same days in 2019–18 in the US cohort, by age and gender, in patients with non-alcoholic fatty liver disease (NAFLD).
(DOCX)

S4 Fig Serum haptoglobin variability. A. Serum haptoglobin variability during covid-19 spread versus the same days in 2019–2018 in the US cohort, by age<55 years versus > = 55 years and gender. B. Serum haptoglobin variability during covid-19 spread versus the same days in 2019–2018 in the US cohort, in NAFLD, by gender and by age <55 years versus > = 55 years. C. Serum haptoglobin variability during covid-19 spread versus the same days in 2019–2018 in the US cohort, in HCV, by gender and by age <55 years versus > = 55 years.
(DOCX)

S5 Fig Serum alpha-2 macroglobulin variability. A. Serum alpha-2 macroglobulin variability during covid-19 spread versus 2019 same days in APHP-PSL hospital, French cohort and US cohort. B. Serum alpha-2 macroglobulin variability during covid-19 spread versus the same days in 2019 in the US cohort, by gender and by age <55 years versus > = 55 years. C. Serum alpha-2 macroglobulin variability during covid-19 spread versus the same days in 2019 in the US cohort, by gender and by age <55 years versus > = 55 years. In NAFLD serum only. D. Serum alpha-2 macroglobulin variability during covid-19 spread versus the same days in 2019 in the US cohort, by gender and by age <55 years versus > = 55 years. In HCV serum only.
(DOCX)

S6 Fig Serum GGT variability. A. Serum GGT variability during covid-19 spread versus the same days in 2019 in the APHP-PSL hospital, French and US cohorts. B. Serum GGT variability during covid-19 spread versus the same days in 2019 in the US cohort, by gender, and age <55 years versus > = 55 years.
(DOCX)

S7 Fig Serum ALT variability. A. Serum ALT variability during covid-19 spread versus the same days in 2019 in the APHP-PSL hospital, French and US cohorts. B. Serum ALT variability during covid-19 spread versus the same days in 2019 in the US cohort, by gender and age.
(DOCX)

S8 Fig Components without significant changes. A. Serum total bilirubin variability during covid-19 spread versus the same days in 2019 in the APHP-PSL hospital, French and US cohorts. B. Serum total cholesterol variability during covid-19 spread versus the same days in 2019 in the APHP-PSL hospital, French and US cohorts. C. Serum total triglycerides variability during covid-19 spread versus the same days in 2019 in the APHP-PSL hospital, French and US cohorts. D. Serum fasting glucose variability during covid-19 spread versus the same days in 2019 in the APHP-PSL hospital, French and US cohorts. E. Height variability during covid-19 spread versus the same days in 2019 in the APHP-PSL hospital, French and US cohorts. F. Weight variability during covid-19 spread versus the same days in 2019 in the APHP-PSL hospital, French and US cohorts.
(DOCX)

S9 Fig Sensitivity analysis of ROC curves. A. Diagnostic performance of apolipoprotein-A1 for the diagnosis of covid-19 in 136 covid-19 cases and 393 healthy blood donors, prevalence = 26%. B. Diagnostic performance of apolipoprotein-A1 for the diagnosis of covid-19 in 136 covid-19 cases and 100 patients with rheumatological diseases, prevalence = 58%. C. Diagnostic performance of apolipoprotein-A1 for the diagnosis of covid-19 in 136 covid-19 cases and 8,335controls (Integrated database, prevalence 1.6%).
(DOCX)
