# Supplementary material for: Performance of serum apolipoprotein-A1 as a sentinel of Covid-19
Source: PLoS One. 2020 Nov 20;15(11):e0242306. doi: 10.1371/journal.pone.0242306 (PMC7679025; doi:10.1371/journal.pone.0242306)
Supplement: S1 File — (DOCX) [file pone.0242306.s001.docx]

**S1 File. Mechanisms of the early decrease in apolipoprotein-A1 before recognition of the pandemic, liver or intestine**

We did not identify any major confounding factors to explain the very-early time-related association between the decrease in apolipoprotein-A1 and the spread of the pandemic in the US cohort of patients at risk of liver fibrosis. Although the mechanisms explaining the decrease in apolipoprotein-A1 in late severe Covid-19 pneumonia are known, the reason for the early decrease before the acute phase, when haptoglobin remained normal is unclear. In patients with severe pneumonia, published evidence-based results showed that the apolipoprotein-A1 decrease was associated with acute inflammation and the “cytokine storm” with an increase in IL6 and acute phase proteins such as CRP and haptoglobin (S2 Table). In our study in the US cohort, the decrease in apolipoprotein-A1 was not associated with an increase in haptoglobin (Fig 1C, S4 Fig). This dissociation suggests that different mechanisms play a role in the early influence of the SARS-CoV2 virus on the synthesis of apolipoprotein-A1.

To our knowledge, there are no validated biomarkers for assessing the intestinal synthesis of apolipoprotein-A1 or the transintestinal cholesterol efflux in normal subjects or in the presence of intestinal disease, despite a better understanding of the possible mechanisms.**^22^** For the liver, the mechanisms are better understood for the synthesis of apolipoprotein-A1 and its decrease in liver diseases. Serum apolipoprotein-A1 can decrease to below 1.25 g/L, only in patients with endstage liver disease, severe alcoholic hepatitis, and severe DILI, and this decrease is associated with a contemporaneous haptoglobin decrease.**^1,2^** Therefore, the profile of a significant and early decrease in apolipoprotein-A1 without an associated increase in haptoglobin, GGT, A2M, and ALT strongly supports a non-liver-related cause. We never observed such profile of biomarkers in our experience since 2001 with more than three million of FibroTest assessed in liver diseases.**^3,4,5^**

Among the possible intestinal mechanisms, the SARS-CoV2 virus could impact several pathways leading to a decrease in the intestinal synthesis and absorption of apolipoprotein-A1 in the small intestine resulting in the decrease in serum.**^6,7,8^**

The first could the inhibition of lysophosphatidylcholine acyltransferase 3 (Lpcat) activity, which is involved in phosphatidylcholine remodeling in the small intestine and liver.**^9,10^** Indeed, in experimental calf pneumonia, Lpcat activity is reduced as early as one day after inoculation of Pasteurella haemolytica of bovine herpes virus-1, inducing a decrease in serum HDL concentrations and thus, in its transporter apolipoprotein-A1, before the haptoglobin increase.**^10^** In knock-out mice, an Lpcat deficiency causes a 90% reduction in total Lpcat activity in the small intestine, but only a 70% reduction in total activity in the liver. **^10,11^** There is evidence of direct SARS-CoV2 infection of the endothelial cell and diffuse endothelial inflammation in the intestine.**^12,13^** SARS-CoV2 could impact several organs as suggested by its ubiquitary effect, on endothelial cells such as enterocyte, pericyte or macrophages.**^13,14^** The virus uses angiotensin-converting enzyme 2 receptor (ACE-2) expressed by pneumocytes to infect the host, causing lung injury. The ACE-2 receptor is also widely expressed on endothelial cells, including in the intestine and liver.**^12,13^**^,^**^14,15,16^** In the liver, SARS-CoV2 is also present,**^14,15^** and the ACE-2 receptor seems to be more highly expressed in cholangiocytes than in hepatocytes.**^17^** Thus far there are no data on the prevalence of the virus in the liver and in the intestine in cases of SARS-CoV2 without severe symptoms.**^18^**

The second mechanism could also be an impact of the virus through the intestinal mucus.**^17^** Apolipoprotein-A1 is released as a free apolipoprotein from the apical side of enterocytes into the lumen in the fasting state. In addition to the main secreted goblet cell components, mucus contained cellular proteins that have longer half-life in mucus than in epithelial cells. Apolipoprotein-A1 had faster turnover in mucus, which could be a target for SARS-CoV2.**^18,19^**

We observed no significant difference, in our limited number of patients with diarrhea, for clinical other symptoms or in the biomarkers of inflammation or liver disease, and no prognostic significance versus the patients without diarrhea. The only significant difference was a decrease in neutrophil count, which has not been yet reported to be associated with diarrhea.**^20^** Only one article observed lower monocyte count and white blood cell count in 103 patients with Covid-19 and digestive symptoms.**^21^**

**References for the S1 File. Apolipoprotein-A1 in liver and intestine**

1. Peta V, Tse C, Perazzo H, et al. Serum apolipoprotein A1 and haptoglobin, in patients with suspected drug-induced liver injury (DILI) as biomarkers of recovery. PloS One 2017; 12:e0189436.

2. Rudler M, Mouri S, Charlotte F, et al. Validation of AshTest as a non-invasive alternative to transjugular liver biopsy in patients with suspected severe acute alcoholic hepatitis. PLoS One 2015; 10:e0134302.

3. Poynard T, Deckmyn O, Munteanu M, et al. Awareness of the severity of liver disease re-examined using software-combined biomarkers of liver fibrosis and necroinflammatory activity. BMJ open 2015; 5:e010017.

4. Poynard T, Munteanu M, Charlotte F, et al. Diagnostic performance of a new noninvasive test for nonalcoholic steatohepatitis using a simplified histological reference. Eur J Gastroenterol Hepatol 2018;**30**:569-577.

5. Poynard T, Munteanu M, Deckmyn O, et al. Applicability and precautions of use of liver injury biomarker FT. A reappraisal at7 years of age. BMC Gastroenterol 2011;**11**:39.

6. Glickman RM, Green PH. The intestine as a source of apolipoprotein A1. Proc Natl Acad Sci USA 1977; **74**:2569–25736.

7. Gu J, Han B, Wang J. COVID-19: Gastrointestinal Manifestations and Potential Fecal-Oral Transmission. Gastroenterology 2020; **158**:1518‐1519.

8. Cao X, Yin R, Albrecht H, Fan D, Tan W. Cholesterol: A new game player accelerating endothelial injuries caused by SARS-CoV-2? [published online ahead of print, 2020 Jun 5]. Am J Physiol Endocrinol Metab. 2020; 10.1152/ajpendo.00255.2020.

9. Li Z, Jiang H, Ding T, et al. Deficiency in lysophosphatidylcholine acyltransferase 3 reduces plasma levels of lipids by reducing lipid absorption in mice. Gastroenterology 2015; **149**:1519–1529.

10. Nakagawa H, Katoh N. Reduced serum lecithin:cholesterol acyltransferase activity and cholesteryl ester concentration in calves experimentally inoculated with Pasteurella haemolytica and bovine herpes virus-1. J Vet Med Sci 1999; **6**:1101-1106.

11. Kabir I, Li Z, Bui HH, et al. Small intestine but not liver lysophosphatidylcholine acyltransferase 3 (lpcat3) deficiency has a dominant effect on plasma lipid metabolism. J Biol Chem 2016; **291**:7651‐7660.

12. Lamers MM, Beumer J, van der Vaart J, et al. SARS-CoV-2 productively infects human gut enterocytes [published online ahead of print, 2020 May 1]. Science. 2020; eabc1669.

13. Xu H, Zhong L, Deng J, et al. High expression of ACE2 receptor of 2019-nCoV on the epithelial cells of oral mucosa. Int J Oral Sci 2020;12:8.

14. Zhang H, Kang ZJ, Gong HY, et al. The digestive system is a potential route of 2019-nCov infection: a bioinformatics analysis based on single-cell transcriptomes.BioRxiv 2020; doi:10.1101/2020.01.30.927806.

15. Zhou P, Yang XL, Wang XG, et al. A pneumonia outbreak associated with a new coronavirus of probable bat origin. Nature. 2020; **579**:270‐273.

16. Ding S, Liang TJ. Is SARS-CoV-2 also an enteric pathogen with potential fecal-oral transmission? A COVID-19 virological and clinical review [published online ahead of print, 17. Chai XQ, Hu LF, Zhang Y, et al. Specific ACE2 Expression in cholangiocytes may cause liver damage after 2019-nCoV Infection. BioRxiv 2020; doi:10.1101/2020.02.03.931766.

2020 Apr 27]. Gastroenterology. 2020; S0016-5085(20)30571-0.

18. Zuo T, Liu Q, Zhang F, et al. Depicting SARS-CoV-2 faecal viral activity in association with gut microbiota composition in patients with COVID-19 [published online ahead of print, 2020 Jul 20]. *Gut*. 2020;gutjnl-2020-322294. doi:10.1136/gutjnl-2020-322294

19. Danielsen EM, Hansen GH, Rasmussen K, et al. Apolipoprotein A-1 (apoA-1) deposition in, and release from, the enterocyte brush border: a possible role in transintestinal cholesterol efflux (TICE)?. Biochim Biophys Acta 2012; **1818(3)**:530‐536.

20. Mao R, Qiu Y, He JS, et al. Manifestations and prognosis of gastrointestinal and liver involvement in patients with COVID-19: a systematic review and meta-analysis [published correction appears in Lancet Gastroenterol Hepatol. 2020 Jul;5(7):e6]. Lancet Gastroenterol Hepatol. **2020**; 5:667-678.

21. Pan L, Mu M, Yang P, et al. Clinical Characteristics of COVID-19 Patients with digestive symptoms in Hubei, China: A descriptive, cross-Sectional, multicenter study. Am J Gastroenterol. 2020; **115**:766-773.

22. Glickman RM, Green PH. The intestine as a source of apolipoprotein A1. Proc Natl Acad Sci USA 1977; **74**:2569–2573.
