# Supplementary material for: Performance of serum apolipoprotein-A1 as a sentinel of Covid-19
Source: PLoS One. 2020 Nov 20;15(11):e0242306. doi: 10.1371/journal.pone.0242306 (PMC7679025; doi:10.1371/journal.pone.0242306)
Supplement: S2 File — (DOCX) [file pone.0242306.s002.docx]

**S2 File. Methods.**

***Ethics***

The components of the FibroTest (FibroSure in USA) and Nash-FibroTest (Nash-FibroSure in USA) were measured in a routine follow-up in patients with a risk of liver fibrosis in France and the USA. The results of the serum databases were anonymous.

***Significant decrease of apolipoprotein-A1 in 2020 sera-cohorts***

In the absence for an ideal group to represent the general population, we used a large US cohort of sera from subjects at risk of liver fibrosis (called the US cohort), a disease present in at least 30% of the general adult US population. We also used two other cohorts of sera from subjects at risk of liver fibrosis, but with an increased risk of Covid-19. The cohort with an intermediate risk was a French group of patients (called the French cohort) who were being followed for liver fibrosis in academic and private laboratories, and the high-risk cohort included patients at the APHP-PSL hospital a reference center for Covid-19 patients since January 2020. The core temporal analysis compared the first 34 weeks (January first to August 20^th^) of consecutive anonymous US sera 2020 *vs*. the sera 2019, from these three routinely followed cohorts. The number for USA was 212,297 sera in 2020, 266,976 sera in 2019, then for the French cohort 20,652 sera in 2020, 28,452 sera in 2019 and for the APHP-PSL cohort 3,122 sera in 2020, and 3,928 in 2019.

All the sera from 2018 were also analyzed for assessing the temporal variability of the biomarkers vs. 2019, two years without Covid-19. These all available sera were the same for 2020 at the updated analysis (2020 August 20^th^) and from January first to December 31^st^ for 2019 and 2018. The number for USA was 212,297 sera in 2020, 407,138 sera in 2019, 383,865 sera in 2018, then for the French cohort 20,652 sera in 2020, 43,963 sera in 2019, 45,067 in 2018, and for the APHP-PSL cohort 3,122 sera in 2020, 6,119 in 2019, and 6,568 in 2018.

Due to the lockdown of populations during the pandemic, both in France and USA the number of the daily number of sera analyzed from January-August 20th, 2020 varied significantly compared to from January-August 20th, 2019 in the three cohorts of patients followed for a risk of liver fibrosis (S2C Fig).

***Confounding factors in sera-cohorts***

The cause of a decrease in apolipoprotein-A1 may be due to direct liver toxicity from SARS-CoV-2,**^1-5^** but also to drug-induced liver disease (DILI) caused by medications such as oral acetaminophen, ibuprofen, clavulinate or chloroquine,**^6^** or to a previous liver disease. An overview estimated that one third of patients with severe Covid-19 had impaired hepatic function, based on standard liver function tests (S2 Table).**^7^** To determine whether the changes in apolipoprotein-A1 were associated with liver fibrosis, we analyzed the daily kinetics of alpha2-macroglobulin (A2M) which is not an acute phase protein in humans but a specific marker of liver fibrosis.**^8^** To determine whether the changes in apolipoprotein-A1 were associated with the cause of liver disease, we analyzed in the US-cohort the kinetics of apolipoprotein-A1 and of all the other components, according to non-alcoholic fatty liver disease (NAFLD) sera, or chronic hepatitis C (HCV) sera.

To determine whether the changes in apolipoprotein-A1 were only associated with the acute phase of Covid-19, the kinetics of haptoglobin changes, a sensitive biomarker of severe acute phase, were compared to those of apolipoprotein-A1 in the three cohorts with different risk of Covid-19.

*Daily association between serum apolipoprotein-A1 and the spread of Covid-19*

The number of confirmed Covid-19 cases in France and in the USA was assessed according to published data from the European Centre for Disease Prevention and Control (<https://ourworldindata.org/coronavirus-data>) (S2A Fig and S2B Fig).

***Patients for assessing the sensitivity and the prognostic value***

Sensitivity was assessed in a prospective non-interventional study of Covid-19 patients hospitalized in APHP-PSL. The pre-inclusion criteria were consecutive patients examined in the Internal Medicine and Immunologic Diseases department between 24/01/2020 and 19/05/2020, with suspected Covid-19, based on the presence of cough, fever, myalgia, fatigue, and sputum. A Covid-19 diagnosis was defined as a SARS-CoV2 PCR-positive in respiratory samples, or in blood. All patients with a negative PCR despite symptoms of Covid-19 and chest computed tomography signs of Covid-19, or patients who had a SARS-CoV2 PCR-positive spouse, were included based on the consensus of four physicians (PC, MR, TP, OL). Severe Covid-19 was defined as a patient who could not remain home and required care in the internal-medicine department (i.e. comorbidities, frailty, and nasal oxygen supplementation). Exclusion criteria were no signed informed consent form, missing data for blood markers and not defined as clinical Covid-19 by consensus due to an absence of positive PCR. No prospective controls were included because it was impossible to have a large representative sample of the general population in the context of the pandemic. The primary endpoint for assessing the prognostic value of apolipoprotein-A1 was the survival without transfer in ICU at 28 days, adjusted on age, gender, haptoglobin, and liver tests. The first patient with Covid-19 was admitted to our hospital on the January 30, 2020, the prospective cohort for assessing the sensitivity and the prognostic began on February 19, 2020, and the last patient of the present report was included on April 21, 2020. A total of 136 consecutive patients with severe Covid-19 (PCR- SARS-CoV-2 positive), but who did not require intensive care were hospitalized in the department of internal medicine.

***Patients for assessing the specificity***

Our goal was to estimate the specificity of apolipoprotein-A1 in a group that was representative of the French population. A prospective group was impossible because of the pandemic. However, we have prospective data on apolipoprotein-A1 from and since the first cohort of alcoholic liver disease in 1982,**^9-10^** and from the FibroFrance cohort.**^11-13^** More recently, we collected six databases before the pandemic, which allowed us to retrospectively validate the specificity in a large group of subjects.**^14-17^** The measurements were all performed on fresh prospectively collected serum and analyzed in the biochemistry unit of the APHP-PSL hospital, with the same biochemical methods as the Covid-19 cases (S3 Table). The core control population to determine the specificity and the choice of the best apolipoprotein-A1 cutoff, was a group of 7,481 healthy volunteers aged 40 years and older, that was representative of the French population.**^11^** To prevent the use of an optimal cutpoint which would bias sensitivity and specificity upwards, we added three populations at risk of false positives: a population of 100 patients with rheumatological diseases resulting in chronic inflammation and possible variations in acute phase proteins,**^6^** and two populations with known marked decreases in apolipoprotein-A1, related to the stage of liver fibrosis**^9,10^** and hepatic insufficiency**^14-15^**, 238 patients with DILI,**^6,14^** and 123 patients with severe alcoholic hepatitis.**^15^** We also included 393 blood donors to assess the specificity in younger controls compared to the core control population.**^16^** During the study period, in the same department 43 patients without suspected Covid-19 were excluded (S4 Table**).** These patients were admitted for mixed severe diseases during the pandemic, and therefore could not be used to assess the specificity of apolipoprotein-A1 in the context of use of an early detection test in the general population.

***Association with diarrhea***

The discordance between the very early decrease in apolipoprotein-A1 in comparison with the later increase in liver injury biomarkers, suggested a possible role of the intestine. In order to identify a clinical or biological profile of patients with a possible intestinal route of infection, we compared the subsets of patients with or without diarrhea.

***Biochemistry***

Apolipoprotein-A1 and haptoglobin, two biomarkers of acute phase disease, A2M, which is not an acute phase protein in humans but a marker of liver fibrosis, and three standard liver function tests that are frequently increased in Covid-19 (S2 Table), as well as gammaglutamyl transpeptidase (GGT), alanine aminotransferase (ALT) and total bilirubin were assessed. Apolipoprotein-A1, haptoglobin, A2M, GGT and ALT component assays were performed on an automatic analyzers Cobas-c701 and Cobas-c501 from Roche Diagnostics (Mannheim, Germany). Protein concentrations were measured according to turbidimetric analytical methods using manufacturer’s reagents for haptoglobin and apolipoprotein-A1, and Diagam (Ghislenghien, Belgium), reagents for A2M. ALT was determined according to IFCC methods with pyridoxal phosphate, and GGT using the Szasz method and calibrator value given for the IFCC. Total bilirubin was assessed according to a diazoreaction. Sera from the surveillance cohorts were analyzed for the same period in the first 34 weeks of 2019 and 2020, as well as for 2018, following BioPredictive (Paris, France) analytical recommendations.**^17^**

***Virology***

The fully automated sample-to-result two-target test Cobas 6800 SARS-CoV-2^®^ (Roche molecular systems, Branchburg, NJ, USA) was used to diagnose SARS-CoV2 in upper respiratory samples.**^17^** The presence of SARS-CoV-2 in lower respiratory samples and blood were tested by Altona RealStar^®^RT-PCR Kit 1.0 (Altona Diagnostics, Hamburg, Germany). Plasma samples were subject to total nucleic acid (TNA) extraction using the NucliSENS easyMAG extraction system (BioMerieux, Paris, France) and PCR was performed using LightCycler® 480 Instrument II (Roche, Switzerland)

**References for S2 File. Methods section.**

1. Zhang C, Shi L, Wang FS. Liver injury in COVID-19: management and challenges. Lancet Gastroenterol Hepatol 2020; **5**:428-430.

2. Zhou P, Yang XL, Wang XG, et al. A pneumonia outbreak associated with a new coronavirus of probable bat origin. Nature. 2020; **579**:270‐273.

3. Kelly BJ, Lautenbach E, Nachamkin I, et al. Combined biomarkers predict acute mortality among critically ill patients with suspected sepsis. Crit Care Med 2018; **46**:1106–1113.

4. Chen N, Zhou M, Dong X, et al. Epidemiological and clinical characteristics of 99 cases of 2019 novel coronavirus pneumonia in Wuhan, China: a descriptive study. Lancet 2020; **395**:507–513.

5. Nakagawa H, Katoh N. Reduced serum lecithin:cholesterol acyltransferase activity and cholesteryl ester concentration in calves experimentally inoculated with Pasteurella haemolytica and bovine herpes virus-1. J Vet Med Sci 1999; **6**:1101-1106.

6. Church RJ, Kullak-Ublick GA, Aubrecht J, et al. Candidate biomarkers for the diagnosis and prognosis of drug-induced liver injury: An international collaborative effort. Hepatology 2019; **69**:760-773.

7. Danielsen EM, Hansen GH, Rasmussen K, et al. Apolipoprotein A-1 (apoA-1) deposition in, and release from, the enterocyte brush border: a possible role in transintestinal cholesterol efflux (TICE)?. Biochim Biophys Acta 2012; **1818(3)**:530‐536.

8. Imbert-Bismut F, Ratziu V, Pieroni L, et al. Biochemical markers of liver fibrosis in patients with hepatitis C virus infection: a prospective study. Lancet 2001; **357**:1069-75.

9. Poynard T, Abella A, Pignon JP, et al. Apolipoprotein AI and alcoholic liver disease. Hepatology 1986; **6**:1391–1395.

10. Bedossa P, Poynard T, Abella A, et al. Apolipoprotein AI is a serum and tissue marker of liver fibrosis in alcoholic patients. Alcohol Clin Exp Res 1989; **13**:829‐833.

11. Poynard T, Lebray P, Ingiliz P, et al. Prevalence of liver fibrosis and risk factors in a general population using non-invasive biomarkers (FibroTest). BMC Gastroenterol 2010; **10**:40.

12. Poynard T, Deckmyn O, Munteanu M, et al. Awareness of the severity of liver disease re-examined using software-combined biomarkers of liver fibrosis and necroinflammatory activity. BMJ open 2015; 5:e010017.

13. Poynard T, Munteanu M, Charlotte F, et al. Diagnostic performance of a new noninvasive test for nonalcoholic steatohepatitis using a simplified histological reference. Eur J Gastroenterol Hepatol 2018;**30**:569-577.

14. Peta V, Tse C, Perazzo H, et al. Serum apolipoprotein A1 and haptoglobin, in patients with suspected drug-induced liver injury (DILI) as biomarkers of recovery. PloS One 2017; 12:e0189436.

15. Rudler M, Mouri S, Charlotte F, et al. Validation of AshTest as a non-invasive alternative to transjugular liver biopsy in patients with suspected severe acute alcoholic hepatitis. PLoS One 2015; 10:e0134302.

16. Perazzo H, Pais R, Munteanu M, et al. Variability in definitions of transaminase upper limit of the normal impacts the APRI performance as a biomarker of fibrosis in patients with chronic hepatitis C: "APRI c'est fini ?". Clin Res Hepatol Gastroenterol 2014; **38**:432‐439.

17. Poljak M, Korva M, Knap Gašper N, et al. Clinical evaluation of the Cobas SARS-CoV-2 test and a diagnostic platform switch during 48 hours in the midst of the COVID-19 pandemic. J Clin Microbiol 2020; pii: JCM.00599-20.
