# Supplementary material for: Performance of serum apolipoprotein-A1 as a sentinel of Covid-19
Source: PLoS One. 2020 Nov 20;15(11):e0242306. doi: 10.1371/journal.pone.0242306 (PMC7679025; doi:10.1371/journal.pone.0242306)
Supplement: S3 File — (DOCX) [file pone.0242306.s003.docx]

**S3 File. Results.**

*Diagnostic performance of apolipoprotein-A1*

A total of 136 consecutive patients with severe Covid-19 (PCR- SARS-CoV-2 positive), but who did not require intensive care were hospitalized in the department of internal medicine. The characteristics of PCR Covid-19 positive patients are presented and in Table 2 and were similar to those published in such severity profiles.**^1^**

The characteristics of patients included for the assessment of specificity are presented in S3 Table, the differences between characteristics of the subsets in S3 Table, and in Fig 2A for the median value of apolipoprotein-A1 and in Fig 2B for haptoglobin at inclusion. These two proteins obviously permitted to identify the Covid subset of severe patients, with low apolipoprotein-A1 and very high haptoglobin value, both highly significant (P<0.001) in comparison with controls (normal values) and severe liver diseases (both very low apolipoprotein-A1 and haptoglobin values).

The area under the characteristics curve (AUROC) in 136 Covid-19 cases and 7,481 controls representative of the French population,**^2^** was 0.978 (0.957-0.988), which outperformed haptoglobin and liver function tests (Fig 2C). Apolipoprotein-A1 at a cutoff of 1.25 g/L, had the best Youden index (86.7%) with a sensitivity of 90.6% (95%CI 84.2-95.1) and a specificity of 96.1% (95%CI 95.7-96.6) for the diagnosis of Covid-19.

For a prevalence of 1.8% (136/7617; 95%CI 1.5-2.1) of Covid-19 cases, the positive predictive value (PPV) was 30.0% (95%CI 25.6-34.7) and the negative predictive value (NPV) was 99.8% (95%CI 99.7-99.9). When adjusted on the range of Covid-19 prevalence predicted in the French population,^32^ the PPV was 40.5% and NPV was 99.7% for the 2.8% lower limit, and PPV was 56.0% and NPV was 99.3% for the 7.2% higher limit.

The specificity in 393 blood donors was similar using the same 1.25 g/L cutoff (94.2%; 95%CI 83.5-93.8) and lower (84.0%; 95%CI 75.3-90.6%) in 100 patients with rheumatological disease. Finally, this database integrated six databases with a total of 8,471 subjects: 136 Covid-19 (prevalence 1.6%; 95%CI 1.3-1.9) and 8,335 controls. Despite the higher risk of false positives and using the pre-defined 1.25 g/L cutoff from the core control population, the specificity of apolipoprotein-A1 remained high with 92.3% (95%CI 91.7-92.8), an AUROC of 0.947 (95%CI 0.930-0.960) and a Youden index=0.834, as well as high VPP and VPN (S9C Fig).

The sensitivity with a cutoff <1.25 g/L was 90.6% (98/116). It is interesting to note that the sensitivity in the 19 patients with negative viral nucleic acid testing was similar 18/19 (94.7%) to those with positive results, 98/109 (89.9%; exact Fisher test P=1).

The prognostic value of apolipoprotein-A1 at inclusion was significant, risk-ratio (RR) =5.61 (95%CI 1.02-31.0; P=0.04), adjusted on age (RR=1.04 95%CI 1.01-1.07; P=0.04), GGT (RR=2.88 95%CI 1.01-8.19; P=0.04), the others being not significant, gender, A2M and haptoglobin, by Cox model for predicting the primary outcome (death, n=16, or transfer in ICU, n=14, total =26 as four deaths occurred among the transfer cases) at 4 weeks. The 71 patients with apolipoprotein-A1 value >= 0.84 g/L, the median value at inclusion, had a significant higher survival without ICU (93.0%; 9%CI 87.0-98.9) than the 65 patients with lower value (75.8%; 95%CI 65.1-86.5; P=0.02) (Fig 2D).

Repeated assessments of 305 sera showed that in patients who survived without ICU, the mean apolipoprotein-A1 raised significantly already at the third sera (median 7 days), 0.89 to 0.96 g/L (P<0.01) (Fig 3A). Among the 16 patients who survived after their transfer to ICU, the mean apolipoprotein-A1 also significantly improved at the second sera (median 3 days), from 0.75 to 0.88 g/L (P=0.04) (Fig 3B).

*Comparison of patients with or without diarrhea*

The prevalence of diarrhea on initial presentation was 29 out of 131 cases (22.1%;95%CI 15.4-30.2). In this subset, the only significant difference was a lower median number of polynuclear leucocytes, 3,235 (IQR 2510-4490) vs 4850 (IQR 3118-6708), P=0.003. No significant difference was observed for the other clinical symptoms and the panel of biomarkers, as well as for the prognostic significance for transfer to ICU or death (S5 Table).

*Center effect on biomarkers prescription*

Using APHP-PSL hospital data, we assessed the risk of other confounding factors in more detail, both in patients with Covid-19 and in controls. After admission of the first patient with Covid-19 on January 30, 2020, several units of the hospital were transformed into a Covid-19 reference center in March 2020. Because of the country-wide lockdown, there was a significant decrease in processed FibroTest prescriptions, with a decrease of 12.5 % in April 2020 in France and in the USA. In APHP-PSL, the processing of prescriptions in April 2020 was still between 35% and 50% compared to a mean 12.5% in France (S2C Fig upper panel). This center-effect can be explained by the conversion of the APHP-PSL hospital into a Covid-19 center with a marked increase in the proportion of severe cases. Interestingly the second peak of GGT was contemporaneous of the re-opening of the Hepatology unit in May, and the associated input of sera of severe fibrosis patients (S6 Fig).

**References for S3 File. Results section**

1. Chen N, Zhou M, Dong X, et al. Epidemiological and clinical characteristics of 99 cases of 2019 novel coronavirus pneumonia in Wuhan, China: a descriptive study. Lancet 2020; **395**:507–513

2. Poynard T, Lebray P, Ingiliz P, et al. Prevalence of liver fibrosis and risk factors in a general population using non-invasive biomarkers (FibroTest). BMC Gastroenterol 2010; **10**:40.
