# Supplementary material for: Performance of serum apolipoprotein-A1 as a sentinel of Covid-19
Source: PLoS One. 2020 Nov 20;15(11):e0242306. doi: 10.1371/journal.pone.0242306 (PMC7679025; doi:10.1371/journal.pone.0242306)
Supplement: S4 File — (DOCX) [file pone.0242306.s004.docx]

**S4 File. Discussion.**

*Confounding factors*

The decrease of serum apolipoprotein-A1 in 2020 *vs.* previous years, as well as the time-related association of serum apolipoprotein-A1 in 2020 and Covid-19 might be due to numerous confounding factors. We acknowledge that consecutive sera were analyzed anonymously and therefore there was an unknown percentage of duplicated subjects with repeated sample over time. In the context of the pandemic it was impossible to perform an ideal prospective study with a population that was representative of the general population, and we used cohorts of subjects requiring surveillance of liver fibrosis biomarkers. Despite these risks of bias, adults at risk of liver fibrosis represent at least 30% of the general adult population in the USA and in France. In these cohorts the median age was over 50, and 70% of the subjects had no (F0) or minimal fibrosis.

The decrease in apolipoprotein-A1 in 2020 compared to 2019, and 2018 cannot be explained by bias due to gender, age, or the cause of liver disease. A decrease in apolipoprotein-A1 could be also explained by a severe liver disease. Our results do not support this hypothesis for two reasons. Firstly, the prevalence of severe cases cannot explain the significant decrease in apolipoprotein-A1 already observed in January 2020. In the 2020 population the prevalence of severe fibrosis (stages F3 and F4) was 20.5% in 2020 *vs* 22.0% in 2019 which corresponds to the decrease in A2M (Table 1 and S5 Fig), probably due to the improvement of fibrosis in patients cured by active antiviral treatment (DAA) of viral hepatitis C (S3 File).^15^ In case of more severe fibrosis in 2020, A2M would have increased.**^13-16^** Secondly, GGT a very sensitive liver biomarkers did not change during the first 3 months (S6 Fig**).**  Thirdly, in these severe liver diseases, haptoglobin should be also significantly decreased (Fig 2A), which was not observed in 2020.

In the US cohort the proportion of sera with NAFLD was increased by 2.0% in 2020 vs 2019 (Table 1). However, no significant changes were observed for total cholesterol**,** triglycerides**,** fasting glucose**,** weight or height (S8 Fig**),** after stratification by age and gender (data not shown). A significant change in such metabolic factor would suggest a confounding cause for apolipoprotein change.

The same significant kinetics in apolipoprotein-A1 levels were observed after stratification of the regression curves for gender and age in the three cohorts. The US-cohort was the only sample that had the necessary power to compare these two factors together between 2020 and 2019 and 2018 (Fig 1C), and in the subsets of patients with HCV or NAFLD (S3 Fig) The higher drops were observed in August 2020 for subjects younger than 55 years both for male and female (Fig 1C). Apolipoprotein-A1 decrease was similar during Covid-19 spread versus 2019 and 2018 in the US-cohort (S2 File, S3 Fig**)**. The kinetics of apolipoprotein-A1 were not associated with those of haptoglobin in the US-cohort. As expected, there was a significant haptoglobin increase at the peak of the pandemic in the French cohorts and particularly in the APHP-PSL cohort (S4 Fig).

For A2M, in the US-cohort, there was a significant lower mean serum value, when compared to 2019 and 2018, and detailed in S5 Fig. This significant decrease of A2M was continuous since January 2018, persisted after stratification by age and gender, but was no more significant in NAFLD sera, for the comparison between 2020 and 2019 years. In HCV sera, the significant decrease of A2M persisted only between the years 2019 vs 2018 after stratification by age and gender.

The other significant differences were, in the French-cohorts, GGT increased during the pandemic peak and returned to previous years’ value thereafter (S6 Fig**)**, in the US-cohort and a transient increase in ALT in April 2020, (S7 Fig)**.**

No changes were observed for total bilirubin S8A Fig, total cholesterol S8B Fig**,** triglycerides S8C Fig**,** fasting glucose S8D Fig**,** height S8E Fig or weight S8F Fig.

No active drugs were marketed for NAFLD or NASH between 2019 and 2020. We found the same kinetics for apolipoprotein A1 or all the other biochemical tests after exclusion of NAFLD sera.

Using APHP-PSL hospital data, we assessed the risk of other confounding factors in more details, both in patients with Covid-19 and in controls. After admission of the first patient with Covid-19 on January 30, 2020, several units of the hospital were transformed into a Covid-19 reference center in March 2020. Because of the country-wide lockdown, there was a significant decrease in processed FibroTest prescriptions, with a decrease of 12.5 % in April 2020 in France and in the USA. In APHP-PSL, the processing of prescriptions in April 2020 was still between 35% and 50% compared to a mean 12.5% in France (S2C Fig upper panel**).** This center-effect can be explained by the conversion of the APHP-PSL hospital into a Covid-19 center with a marked increase in the proportion of severe cases. Interestingly the second peak of GGT was contemporaneous of the re-opening of the Hepatology unit in May, and the associated input of sera of severe fibrosis patients (S6A Fig).

In the US-cohort, ALT was the only biomarker of the standard liver function tests which increased significantly at the 13^th^ week of 2020, above the usual mean value observed in 2019 (S7 Fig). This increase in ALT in April and May was not associated with changes of fasting glucose (S8D Fig**)**, height (S8E Fig**)** or weight (S8F Fig**)**. We have no clear explanation. We hypothesize that another confounding factor could be moderate DILI, including oral acetaminophen or hydroxychloroquine misuse during the pandemic. Such factor could explain an increase in ALT only, without increase in haptoglobin, in subjects with mild symptoms.

Also, very intriguing was the absence of haptoglobin increase, in the early period of apolipoprotein-A1 decrease. It was known that in patients with a progression of liver fibrosis these two proteins decrease. The absence of haptoglobin change associated with apolipoprotein-A1 decrease, has never been described before. In the US cohort, the decrease in apolipoprotein-A1 was almost linear (Fig 1B), and haptoglobin remained stable (S4 Fig). The 34 weeks followup, permitted to see the return to normal values of haptoglobin in the APHP-PSL cohort, associated to the decrease of severe Covid-19 cases admissions (S4A Fig**)**. Furthermore, the patients followed in the prospective study who had a recovery, had both a significant increase apolipoprotein-A1 (S9A Fig) and a significant decrease in haptoglobin (S9B Fig) repeated values, 10 days after inclusion.

This negative association between apolipoprotein-A1 and haptoglobin well known in severe pneumonia (S1 Table**)** was only observed in the two French cohorts with high prevalence of severe Covid-19 and not in the larger US cohort (S2B Fig**,** S4A Fig).

*Variability of A2M*

There was a rational explanation for the lower value of A2M observed in the US cohort already observed in January 2020 in comparison with January 2019. Indeed, A2M decreased regularly since January 2018. These results could be explained by the improvement of fibrosis in patients cured by active antiviral treatment (DAA) of viral hepatitis C, probably the main cause of liver fibrosis surveillance. This difference was mostly observed in sera of males with HCV and 55 years of age or older, which is the usual profile of treated chronic hepatitis C.**^15^**

**References for S4 File.**

1. Chien JY, Jerng JS, Yu CJ, et al. Low serum level of high-density lipoprotein cholesterol is a poor prognostic factor for severe sepsis. Critical care Medicine 2005; 33:1688-93.

2. Lekkou A, Mouzaki A, Siagris D, et al. Serum lipid profile, cytokine production, and clinical outcome in patients with severe sepsis. J Crit Care 2014; 29:723-7.

3. Cirstea M, Walley KR, Russell JA, eta la. Decreased high-density lipoprotein cholesterol level is an early prognostic marker for organ dysfunction and death in patients with suspected sepsis. J Crit Care 2017; 38:289-294.

4. Lee SH, Park MS, Park BH, et al. Prognostic implications of serum lipid metabolism over time during sepsis. BioMed research international 2015; 2015:789298.

5. Genga KR, Russell JA. Update of Sepsis in the Intensive Care Unit. J Innate Immun 2017; 9:441-455.

6. Trinder M, Walley KR, Boyd JH, et al. Causal Inference for Genetically Determined Levels of High-Density Lipoprotein Cholesterol and Risk of Infectious Disease. Arterioscler Thromb Vasc Biol 2020; 40:267‐278.

7. Janz DR, Bastarache JA, Peterson JF, et al. Association between cell-free hemoglobin, acetaminophen, and mortality in patients with sepsis: an observational study. Crit Care Med 2013; 41:784-90.

8. Delanghe J, Langlois M, Ouyang J, et al. Effect of haptoglobin phenotypes on growth of Streptococcus pyogenes. Clin Chem Lab Med 1998; 36:691-6.

9. Kelly BJ, Lautenbach E, Nachamkin I, et al. Combined biomarkers predict acute mortality among critically ill patients with suspected sepsis. Crit Care Med 2018; 46:1106–1113.

10. Cholette JM, Pietropaoli AP, Henrichs KF, et al. Elevated free hemoglobin and decreased haptoglobin levels are associated with adverse clinical outcomes, unfavorable physiologic measures, and altered inflammatory markers in pediatric cardiac surgery patients. Transfusion 2018; 58:1631-1639.

11. Ward MD, Brueggemann EE, Kenny T, et al. Characterization of the plasma proteome of nonhuman primates during Ebola virus disease or melioidosis: a host response comparison. Clinical proteomics 2019; 16:7.

12. Imbert-Bismut F, Ratziu V, Pieroni L, et al. Biochemical markers of liver fibrosis in patients with hepatitis C virus infection: a prospective study. Lancet 2001; 357:1069-75.

13. Poynard T, Vergniol J, Ngo Y, et al. Staging chronic hepatitis C in seven categories using fibrosis biomarker (FibroTest™) and transient elastography (FibroScan®). J Hepatol 2014; 60:706-14.

14. Poynard T, Vergniol J, Ngo Y, et al. Staging chronic hepatitis B into seven categories, defining inactive carriers and assessing treatment impact using a fibrosis biomarker (FibroTest®) and elastography (FibroScan®). J Hepatol 2014; 61:994-1003.

15. Xu XY, Kong H, Song RX, et al. The effectiveness of noninvasive biomarkers to predict hepatitis B-related significant fibrosis and cirrhosis: a systematic review and meta-analysis of diagnostic test accuracy. PLoS One 2014; 9:e100182.

16. Naveau S, Gaudé G, Asnacios A, et al. Diagnostic and prognostic values of noninvasive biomarkers of fibrosis in patients with alcoholic liver disease. Hepatology 2009; 49:97-105.

17. Thiele M, Madsen BS, Hansen JF, et al. Accuracy of the Enhanced Liver Fibrosis Test vs FibroTest, Elastography, and Indirect Markers in Detection of Advanced Fibrosis in Patients With Alcoholic Liver Disease. Gastroenterology 2018; 154:1369-1379.

18. Munteanu M, Tiniakos D, Anstee Q, et al. Diagnostic performance of FibroTest,SteatoTest and ActiTest in patients with NAFLD using the SAF score as histological reference. Aliment Pharmacol Ther 2016; 44:877-89.
