# Supplementary material for: Performance of serum apolipoprotein-A1 as a sentinel of Covid-19
Source: PLoS One. 2020 Nov 20;15(11):e0242306. doi: 10.1371/journal.pone.0242306 (PMC7679025; doi:10.1371/journal.pone.0242306)
Supplement: S1 Table — (DOCX) [file pone.0242306.s005.docx]

**S1 Table.** Review of the articles validating the prognostic value of apolipoprotein-A1, haptoglobin, and HDL-cholesterol for overall mortality, in severe sepsis and liver diseases

|  | **Tests** | **Reference** |
| --- | --- | --- |
| **Sepsis^1^** |  |  |
| Pneumonia | HDL-C | Chien 2005 ^1^ |
| Mixed | HDL-C | Lekkou 2014 ^2^ |
| Mixed | HDL-C | Cirstea 2017 ^3^ |
| Mixed | HDL-C | Lee 2015 ^4^ |
| Mixed | HDL-C | Genga 2017 ^5^ |
| Mixed | HDL-C | Trinder 2020 ^6^ |
| Mixed | Haptoglobin | Janz 2013 ^7^ |
| HIV not treated | Haptoglobin | Delanghe 1998 ^8^ |
| Mixed | Haptoglobin | Kelly 2018 ^9^ |
| Mixed children | Haptoglobin | Cholette 2018 ^10^ |
| Primates Ebola infected | Apolipoprotein A1, haptoglobin | Ward 2019 ^11^ |
| **Liver Diseases** |  |  |
| Hepatitis C | Apolipoprotein A1, haptoglobin | Imbert Bismut 2001^12^, Poynard 2014a^13^ |
| Hepatitis B | Apolipoprotein A1, haptoglobin | Poynard 2014b ^14^, Xu 2014 ^15^ |
| Alcoholic liver disease | Apolipoprotein A1, haptoglobin | Naveau 2009 ^16^, Thiele 2018 ^17^ |
| Non-alcoholic liver disease/ NASH | Apolipoprotein A1, haptoglobin | Munteanu 2016^18^, Munteanu 2018^19^ |

^1^HDL-C (high density cholesterol) is strongly associated with apolipoprotein-A1, its transporter (r=0.924, Welsh 2019) and can be used as a surrogate prognostic marker
