# Supplementary material for: Performance of serum apolipoprotein-A1 as a sentinel of Covid-19
Source: PLoS One. 2020 Nov 20;15(11):e0242306. doi: 10.1371/journal.pone.0242306 (PMC7679025; doi:10.1371/journal.pone.0242306)
Supplement: S2 Table — (DOCX) [file pone.0242306.s006.docx]

**S2 Table**. Comorbidity with liver disease and liver dysfunction in patients with covid-19

| **Patients covid-19** | | | **Liver disease (%)** | **Abnormal liver test (%)** |
| --- | --- | --- | --- | --- |
| Guan et al ^1^ | 1099 | 23 (2.3%) hepatitis B | | AST (22.2%), ALT (21.3%) |
| Huang et al ^2^ | 41 | 1 (2.0%) | | 15 (31.0%) |
| Chen et al ^3^ | 99 | Unknown | | 43 (43.0%) |
| Wang et al ^4^ | 138 | 4 (2.9%) | | Unknown |
| Shi et al ^5^ | 81 | 7 (8.6%) | | 43 (53.1%) |
| Xu et al ^6^ | 62 | 7 (11.0%) | | 10 (16.1%) |
| Yang et al ^7^ | 52 | Unknown | | 15 (29.0%) |
| Zhang et al ^8^ | 56 | 2 (3.6%) | | 16 (28.6%) |

Two cases of acute liver failure were observed ^2, 8^, including one related death ^8^

**References:**

1. Guan WJ, NI ZY, Hu Y, et al. Clinical Characteristics of Coronavirus Disease 2019 in China. N Engl J Med 2020; 382:1708-1720.
2. Huang C, Wang Y, Li X, et al. Clinical features of patients infected with 2019 novel coronavirus in Wuhan, China. Lancet 2020; 395:497-506.
3. Chen N, Zhou M, Dong X, et al. Epidemiological and clinical characteristics of 99 cases of 2019 novel coronavirus pneumonia in Wuhan, China: a descriptive study. Lancet 2020; 395:507-513.
4. Wang D, Hu B, Hu C, et al. Clinical characteristics of 138 hospitalised patients with 2019 novel coronavirus-infected pneumonia in Wuhan, China. JAMA 2020; doi: 10.1001/jama.2020.1585.
5. Shi H, Han X, Jiang N, et al. Radiological findings from 81 patients with COVID-19 pneumonia in Wuhan, China: a descriptive study. Lancet Infect Dis 2020; 20:425-434.
6. Xu XW, Wu XX, Jiang XG, et al. Clinical findings in a group of patients infected with the 2019 novel coronavirus (SARS-Cov-2) outside of Wuhan, China: retrospective case series. BMJ 2020; 368:m606.
7. Yang X, Yu Y, Xu J, et al. Clinical course and outcomes of critically ill patients with SARS-CoV-2 pneumonia in Wuhan, China: a single-centered, retrospective, observational study. Lancet Respir Med 2020; 8:475-481.
8. Zhang C, Shi L, Wang FS. Liver injury in COVID-19: management and challenges. Lancet Gastroenterol Hepatol 2020; 5:428-430.
