# Supplementary material for: Performance of serum apolipoprotein-A1 as a sentinel of Covid-19
Source: PLoS One. 2020 Nov 20;15(11):e0242306. doi: 10.1371/journal.pone.0242306 (PMC7679025; doi:10.1371/journal.pone.0242306)
Supplement: S3 Table — (DOCX) [file pone.0242306.s007.docx]

**S3 Table.** Baseline demographic and clinical characteristics of the 43 patients pre-included during the same period, who were not considered to have covid-19 by consensus, and not included in the prospective observational study.

| **Characteristics** | | **Total** |
| --- | --- | --- |
| Number n (%) | | 43 |
| Median age (IQR) year | | 57.5 (44.4-71.6) |
| Age category | |  |
| <50 year | | 16 (37.2) |
| 50 to <70 year | | 10 (23.3) |
| >=70 year | | 17 (39.5) |
| Male sex | | 21 (48.8) |
| Geographic origin | |  |
| Caucasian | | 29 (67.5) |
| Subsaharan | | 9 (20.9) |
| North African, Middle East | | 5 (11.6) |
| Oxygen-support category | |  |
| Invasive oxygen support | | 0 (0-0) |
| Noninvasive oxygen support | | 3 (7.1) |
| None | | 37 (92.9) |
| Coexisting conditions | |  |
| Hypertension | | 21 (48.8) |
| Diabetes | | 14 (32.6) |
| Hyperlipidemia | | 15 (34.9) |
| Liver disease | | 9 (42.9) |
| Pulmonary disease | | 6 (14.0) |
| Severe disease mixed | | 31 (88.6) |
| Tobacco (ongoing or stopped) | | 15 (34.9) |
| Alcohol consumption | | 12 (30.3) |
| Median laboratory (IQR) | |  |
| Apolipoprotein-A1 g/liter | | 0.95 (0.73-1.23) |
| Haptoglobin g/liter | | 1.91 (1.19-2.98) |
| Alpha-2 macroglobulin g/liter | | 1.54 (1.29-1.99) |
| GGT IU per liter | | 53 (26-114) |
| ALT IU per liter | | 34 (23-55) |
| Total bilirubin micromol/L | | 8 (4-18) |
| Platelets 10^6^ per ml | | 228 (165-314) |
| Creatinine µmol per liter | | 74 (54-97) |
| Treatment at risk of DILI | |  |
| Paracetamol oral (2-4 g/day) | | 7 (16.7) |
| Antibiotics | |  |
| None | | 34 (79.1) |
| Without clavulinate | | 5 (11.6) |
| With clavulinate | | 5 (9.3) |
| Hydroxychloroquine (for sarcoidosis) | | 1 (97.6) |
| Fibrosis stage by FibroTest (%) | |  |
| F0 <=0.27 | | 17 (42.5) |
| F1 <=0.48 | | 8 (20.0) |
| F2 <=0.58 | | 3 (7.5) |
| F3 <=0.68 | | 1 (2.5) |
| F4 <=0.74 | | 11 (27.5) |
| Non interpretable | | 0 (0.0) |
| Missing | | 3 (7.0) |
|  | | |
|  |  |  |
|  |  |  |
|  |  |  |
|  |  |  |
|  |  |  |
