# Supplementary material for: Performance of serum apolipoprotein-A1 as a sentinel of Covid-19
Source: PLoS One. 2020 Nov 20;15(11):e0242306. doi: 10.1371/journal.pone.0242306 (PMC7679025; doi:10.1371/journal.pone.0242306)
Supplement: S4 Table — (DOCX) [file pone.0242306.s008.docx]

**S4 Table.** Baseline demographic and clinical characteristics of the patients Covid-19 included in the prospective observational study, according to initial diarrhea.

| **Characteristics** |  | **COVID-19 initial symptom** | | | | |  | |  |
| --- | --- | --- | --- | --- | --- | --- | --- | --- | --- |
|  | | | **Diarrhea** |  |  | **No Diarrhea** | | **P value** | |
| Number n (%) | | | 29 (100%) |  |  | 102 (100%) | |  | |
| Median age (IQR) year | | | 65.1 (56.2-79.7) |  |  | 71.7 (58.8-82.8) | | 0.34 | |
| Age category | | |  |  |  |  | | 0.76 | |
| <50 year | | | 4 (13.8) |  |  | 13 (12.8) | |  | |
| 50 to <70 year | | | 11 (33.9) |  |  | 32 (31.4) | |  | |
| >=70 year | | | 14 (48.3) |  |  | 57 (55.9) | |  | |
| Male sex | | | 18 (61.5) |  |  | 57(55.9) | | 0.55 | |
| Time clinic-inclusion (days) | | | 9 (6-14) |  |  | 9 (5-14) | | 0.74 | |
| Geographic origin | | |  |  |  |  | | 0.78 | |
| Caucasian | | | 62 (56.9) |  |  | 63 (71.7) | |  | |
| Subsaharan | | | 4 (17.8) |  |  | 13 (12.8) | |  | |
| North African, Middle East | | | 6 (20.7) |  |  | 15 (14.7) | |  | |
| Asian | | | 4 (10.1) |  |  | 11 (10.8) | |  | |
| Oxygen-support category | | |  |  |  |  | | 0.19 | |
| Invasive oxygen support | | | 0 |  |  | 0 | |  | |
| Noninvasive oxygen support | | | 18 (62.1) |  |  | 76 (74.5) | |  | |
| None | | | 11 (37.9) |  |  | 26 (25.5) | |  | |
| Coexisting conditions | | |  |  |  |  | |  | |
| Hypertension | | | 13 (44.8) |  |  | 58 (56.9) | | 0.25 | |
| Diabetes | | | 5 (17.2) |  |  | 27 (26.7) | | 0.40 | |
| Hyperlipidemia | | | 6 (20.7) |  |  | 30 (29.4) | | 0.35 | |
| Liver disease | | | 3 (10.3) |  |  | 11 (10.7) | | 1.00 | |
| Pulmonary disease | | | 6 (20.7) |  |  | 8 (7.9) | | 0.08 | |
| Severe disease associated | | | 15 (51.7) |  |  | 58 (57.4) | | 0.59 | |
| Tobacco (ongoing or stopped) | | | 12 (41.4) |  |  | 29 (28.4) | | 0.19 | |
| Alcohol consumption | | | 4 (13.8) |  |  | 15 (14.7) | | 0.90 | |
| Initial presentation | | |  |  |  |  | |  | |
| Anosmia | | | 4 (14.3) |  |  | 11 (11.6) | | 0.74 | |
| Ageusia | | | 3 (10.7) |  |  | 16 (16.8) | | 0.56 | |
| Headache | | | 5 (17.9) |  |  | 9 (9.5) | | 0.31 | |
| Rhinorrhea | | | 0 (0) |  |  | 6 (6.3) | | 0.34 | |
| Dyspnea | | | 19 (67.9) |  |  | 48 (50) | | 0.10 | |
| Wheezing | | | 0 (0) |  |  | 3 (3.1) | | 1.00 | |
| Cough without spitting | | | 9 (32.1) |  |  | 44 (45.8) | | 0.20 | |
| Cough with spitting | | | 5 (17.9) |  |  | 15 (15.6) | | 0.77 | |
| Fatigue | | | 18 (64.3 |  |  | 44 (46.3) | | 0.32 | |
| Median laboratory (IQR) | | |  |  |  |  | |  | |
| Apolipoprotein-A1 g/L | | | 0.87 (0.68-0.98) |  |  | 0.83 (0.70-1.05) | | 0.89 | |
| Apolipoprotein <= 1.25g/L (%) | | | 1 (3.5) |  |  | 11 (10.8) | | 0.30 | |
| Haptoglobin g/L | | | 2.98 (2.01-3.91) |  |  | 3.17 (2.23-4.19) | | 0.31 | |
| Alpha-2 macroglobulin g/L | | | 1.47 (1.12-1.88) |  |  | 1.50 (1.24-2.07) | | 0.38 | |
| GGT IU /L | | | 49 (32-105) |  |  | 50 (30-119) | | 0.92 | |
| ALT IU /L | | | 34 (28-46) |  |  | 29 (19-52) | | 0.41 | |
| Total bilirubin micromol/L | | | 8 (6-9) |  |  | 8 (7-9) | | 0.92 | |
| Platelets 106 per ml (6 missing) | | | 220 (132-285) |  |  | 221 (169-290) | | 0.53 | |
| Creatinine µmol /L (6 missing) | | | 78 (63-122) |  |  | 79 (63-103) | | 0.76 | |
| Fasting Glucose (24 missing) | | | 6 (5.2-7.95) |  |  | 6.1 (5.4-7.93) | | 0.79 | |

| Albumin g/L (13 missing) | 30 (27-32) | 31 (27-34) | 0.15 |
| --- | --- | --- | --- |
| Procalcitonin (12 missing) | 0.135 (0.078-0.230) | 0.130 (0.080-0.280) | 0.98 |
| C-reactive protein (8 missing) | 56 (29-78) | 60 (17-106) | 0.61 |
| InterLeukin-6 (33 missing) | 31.4 (9.55-45.58) | 30 (14.83-61.78) | 0.56 |
| CPK IU /L (13 missing) | 69 (42-144) | 109 (46-241) | 0.21 |
| AST IU /L (6 missing) | 49 (31-62) | 41 (29-57) | 0.27 |
| LDH (11 missing) | 343 (262-429) | 347 (282-420) | 0.98 |
| D-dimer (19 missing) | 1245 (645-1925) | 1115 (548-2230) | 0.69 |
| Troponin (9 missing) | 12.95 (8.06-31.08) | 17.45 (10.1-39.33) | 0.19 |
| Prothrombin time % (11 missing) | 92 (89-97) | 88 (78-100) | 0.27 |
| White cells |  |  |  |
| Neutrophil (9 missing) | 3235 (2510-4490) | 4850 (3118-6708) | 0.003 |
| Eosinophil (7 missing) | 0 (0-48) | 0.1 (0-30) | 0.98 |
| Lymphocyte (9 missing) | 975 (655-1358) | 975 (685-1265) | 0.97 |
| Hemoglobin g/L (7 missing) | 12.2 (11.2-14.5) | 12.3 (11.1-13.5) | 0.67 |

| Treatment at risk of DILI |  |  |  |  |  |
| --- | --- | --- | --- | --- | --- |
| Paracetamol oral (2-4 g/day) | 13 (44.8) |  |  | 33 (32.7) | 0.21 |
| Antibiotics |  |  |  |  | 0.92 |
| None | 6 (20.7) |  |  | 20 (19.6) |  |
| Without clavulinate | 41 (37.6) |  |  | 9 (47.4) |  |
| With clavulinate | 16 (55.2) |  |  | 38 (37.3) |  |
| Steroids | 2 (6.9) |  |  | 11 (9.9) | 0.54 |
| Hydroxy chloroquine | 15 (51.7) |  |  | 38 (37.3) | 0.16 |
| No Intensive care no death | 25 (86.2) |  |  | 74 (73.3) | 0.15 |
| Transfer intensive care unit | 2 (6.9) |  |  | 18 (16.8) | 0.24 |
| Death | 3 (10.3) |  |  | 13 (12.9) | 0.73 |
