# Supplementary material for: Performance of serum apolipoprotein-A1 as a sentinel of Covid-19
Source: PLoS One. 2020 Nov 20;15(11):e0242306. doi: 10.1371/journal.pone.0242306 (PMC7679025; doi:10.1371/journal.pone.0242306)

**S1 Fig.** Prognostic value of HDL cholesterol, a surrogate of apolipoprotein-A1

**S1A Fig.** Prognostic value of HDL cholesterol, a surrogate of apolipoprotein-A1 (meta-analysis from Liu 2020).


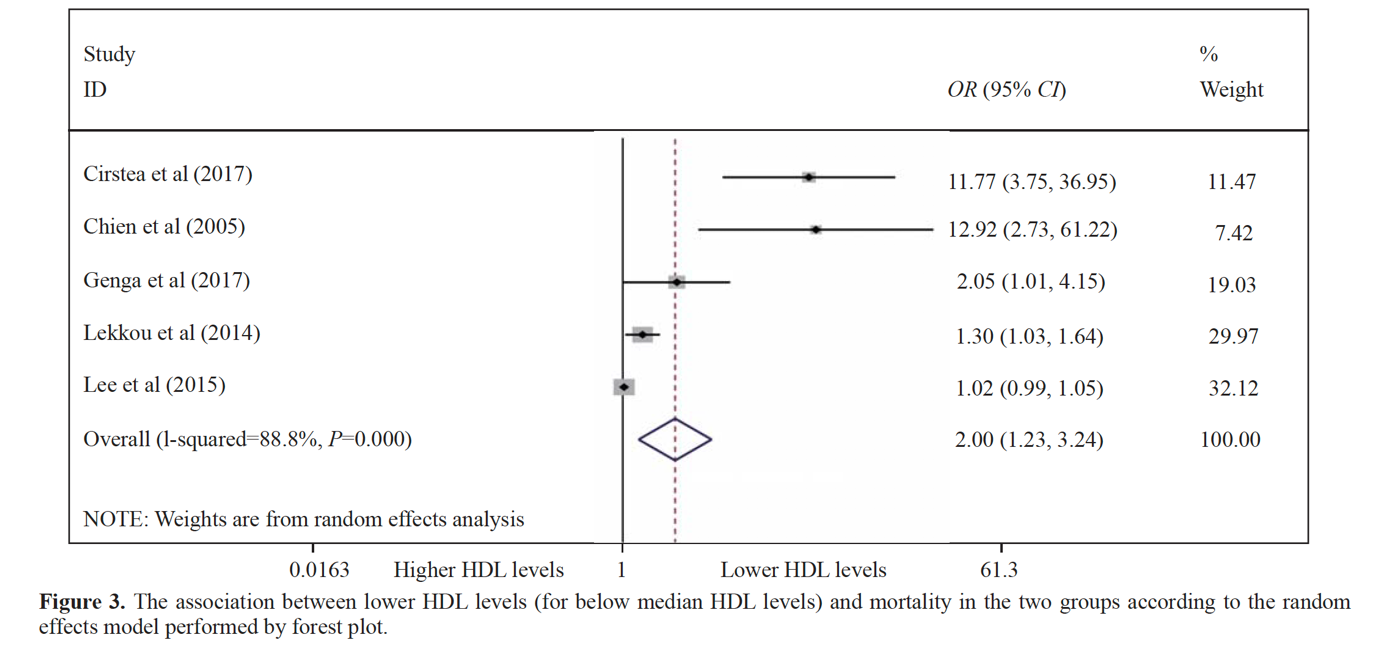


**S1B Fig.** Prognostic value of HDL cholesterol, a surrogate of apolipoprotein-A1 (from Cirstea 2017)


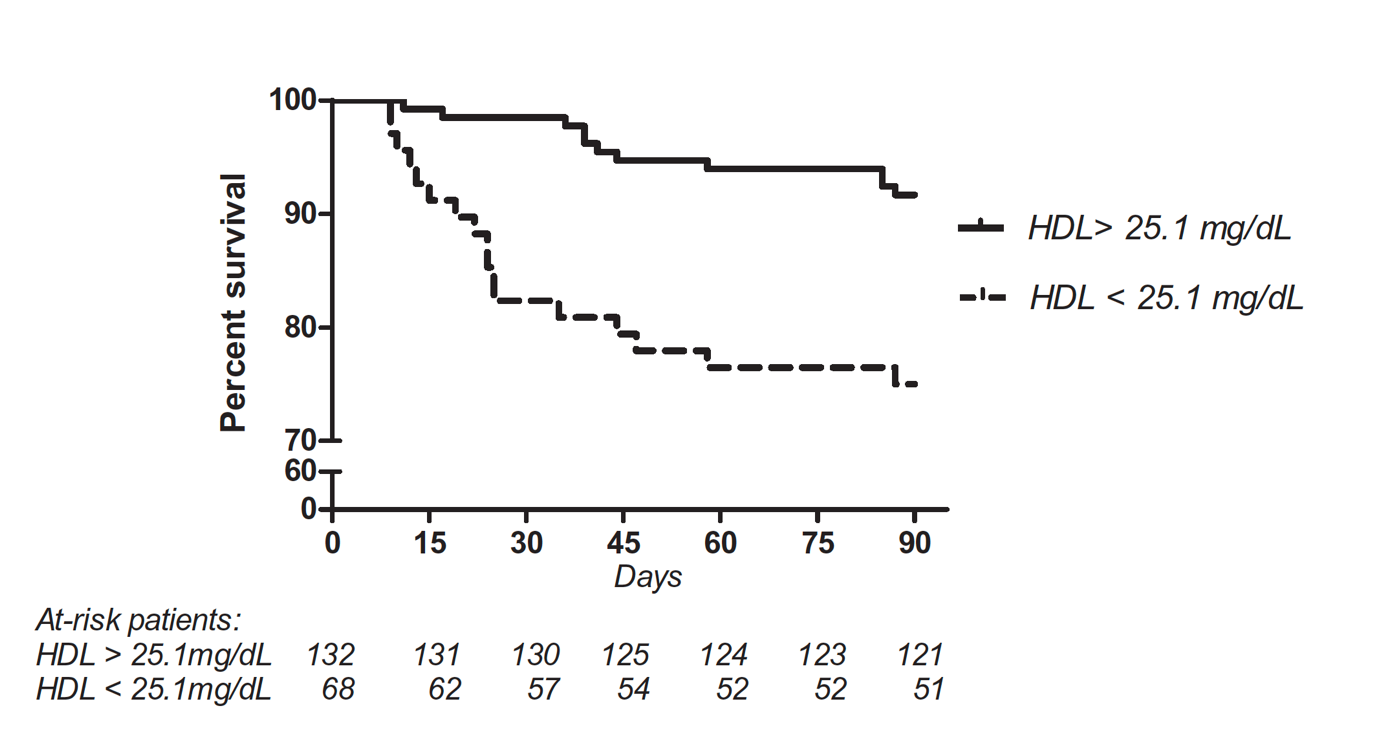

Supplement: S1 Fig — A. Prognostic value of HDL cholesterol, a surrogate of apolipoprotein-A1 (meta-analysis from Liu 2020). B. Prognostic value of HDL cholesterol, a surrogate of apolipoprotein-A1 (from Cirstea 2017). (DOCX) [file pone.0242306.s009.docx]
