# Supplementary material for: Performance of serum apolipoprotein-A1 as a sentinel of Covid-19
Source: PLoS One. 2020 Nov 20;15(11):e0242306. doi: 10.1371/journal.pone.0242306 (PMC7679025; doi:10.1371/journal.pone.0242306)
Supplement: S2 Fig — A. Number of confirmed covid-19 cases in France. B. Number of confirmed covid-19 cases in USA. C. Variability in the daily number of sera analyzed from January-August 20th 2020 (red bars and line with 95% confidence interval) compared to from January-August 20th 2019 (blue) in the APHP-PSL hospital, (upper panel), in the French cohort (upper panel) and in the US cohort (lower panel). The variability is expressed as the processing of tests (ratio between daily numbers) in 2020 vs 2019. (DOCX) [file pone.0242306.s010.docx]

**S2 Fig**. Daily number of confirmed covid-19 cases between January 1^st^ to August 20^th^, 2020.

**S2A Fig.** Number of confirmed covid-19 cases in France

**
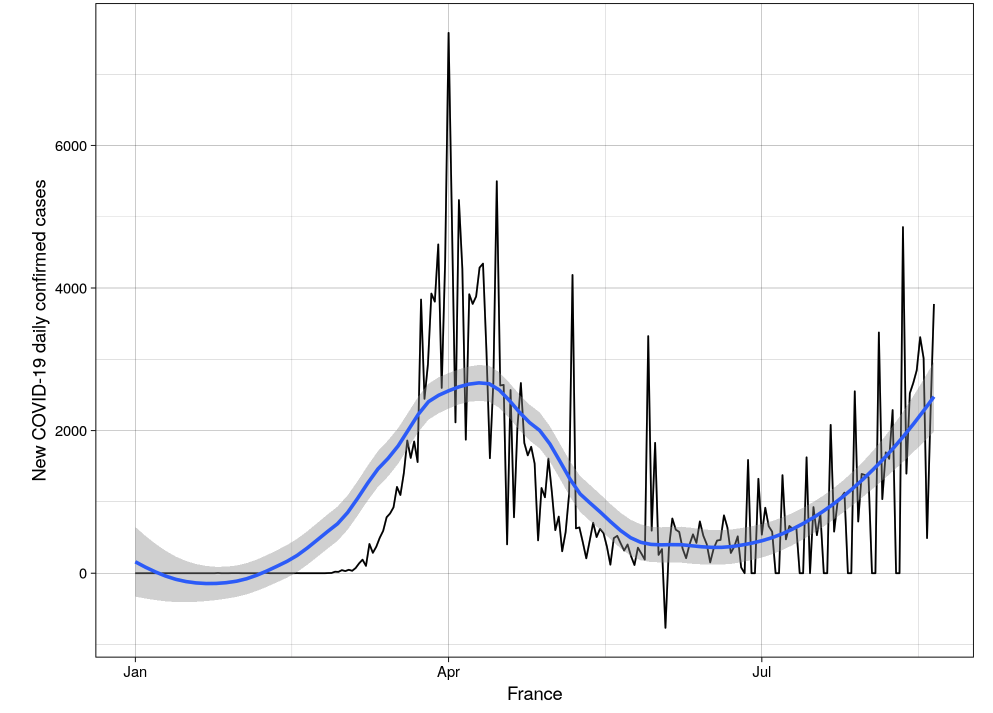
**

**S2B Fig.** Number of confirmed covid-19 cases in USA

**
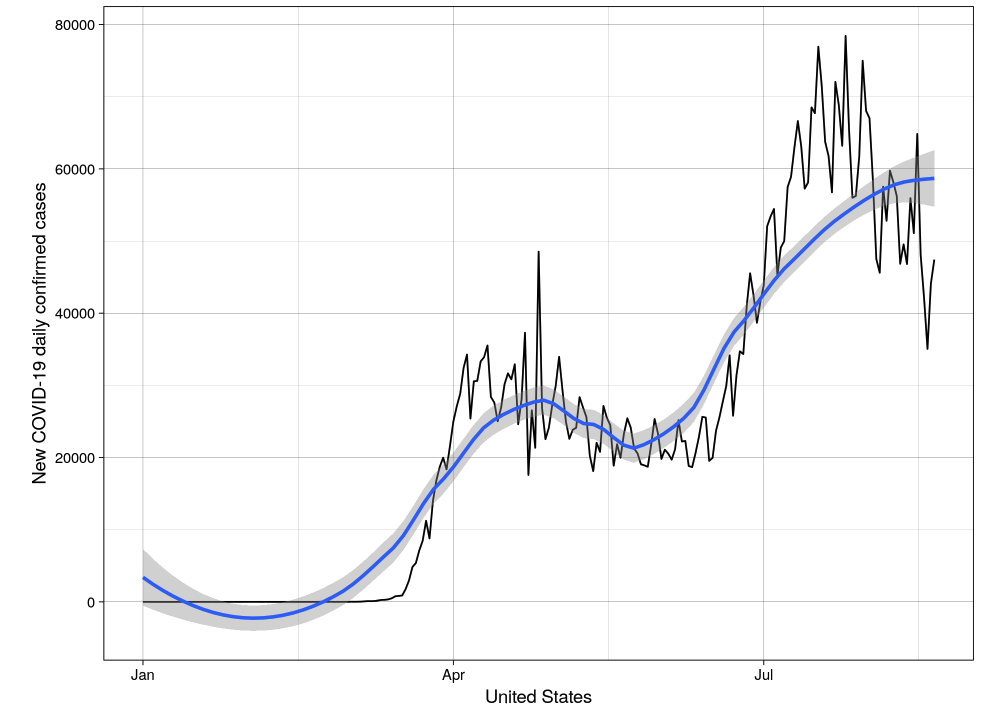
**

**S2C Fig.** Variability in the daily number of sera analyzed from January-August 20th 2020 (red bars and line with 95% confidence interval) compared to from January-August 20th 2019 (blue) in the APHP-PSL hospital, (upper panel), in the French cohort (upper panel) and in the US cohort (lower panel). The variability is expressed as the processing of tests (ratio between daily numbers) in 2020 vs 2019.

**
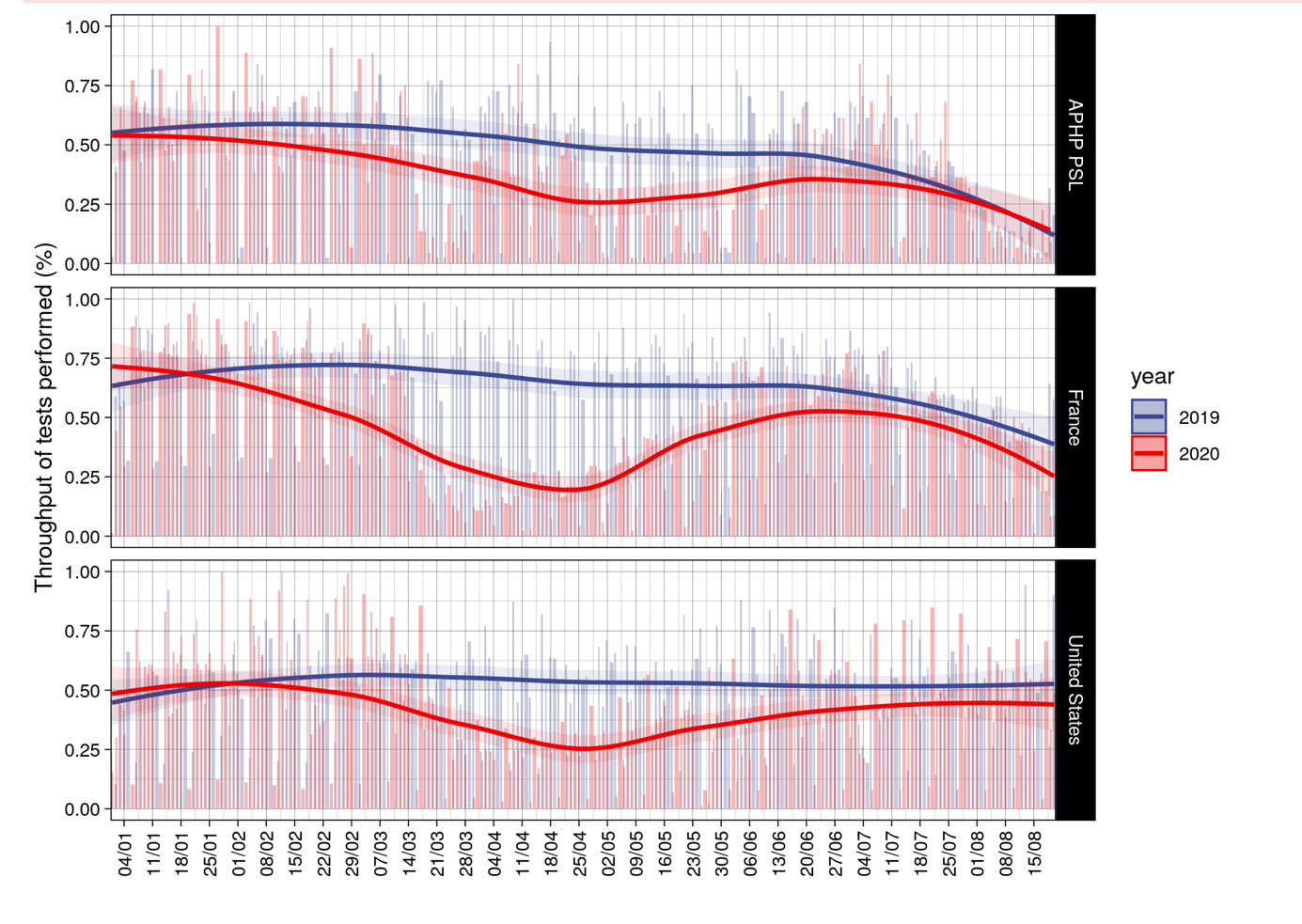
**
