# Supplementary material for: Performance of serum apolipoprotein-A1 as a sentinel of Covid-19
Source: PLoS One. 2020 Nov 20;15(11):e0242306. doi: 10.1371/journal.pone.0242306 (PMC7679025; doi:10.1371/journal.pone.0242306)

**S3 Fig.** Serum apolipoprotein ApoA1 variability

**S3A Fig.** Serum apolipoprotein ApoA1 variability during covid-19 spread versus the same days in 2019-2018 in the APHP-PSL hospital, French and US cohorts, by gender.


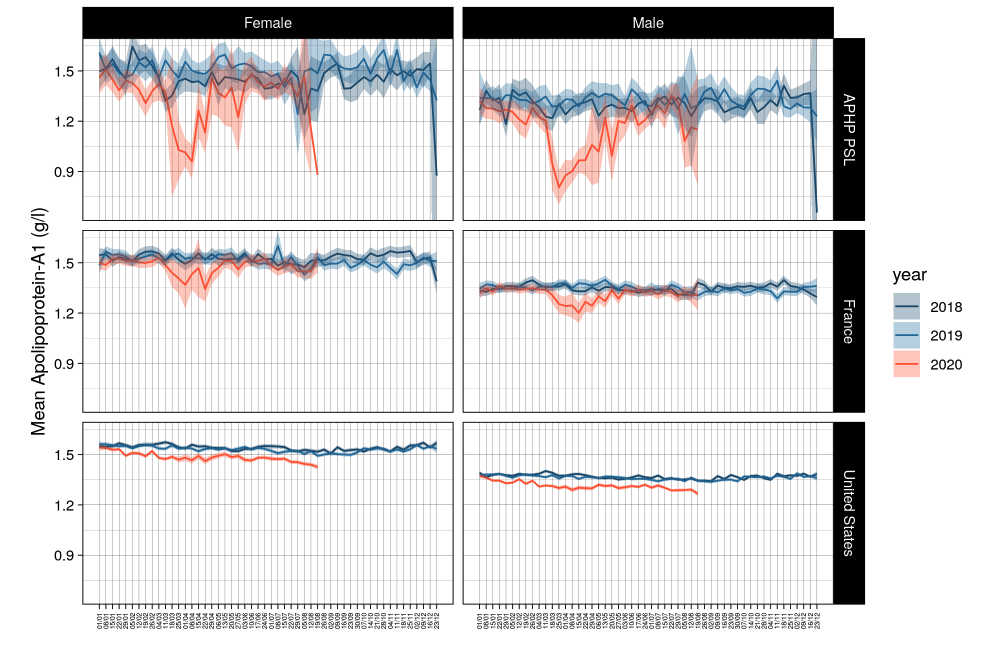


**S3B Fig.** Serum apolipoprotein ApoA1 variability during covid-19 spread versus the same days in 2019-2018 in the APHP-PSL hospital, French and US cohorts, by age.


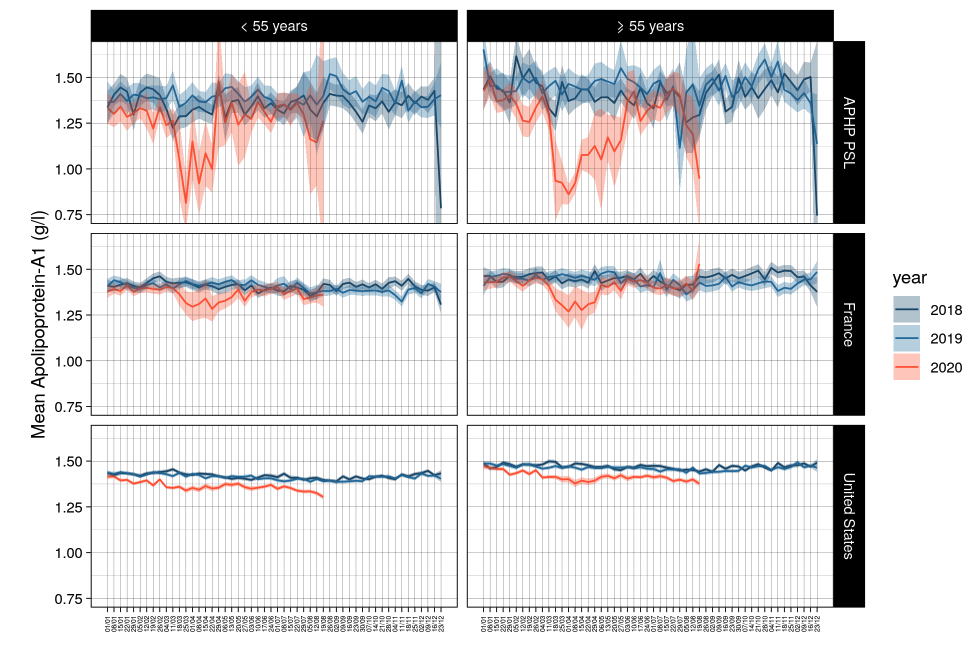


**S3C Fig.** Serum apolipoprotein ApoA1 variability during covid-19 spread versus the same days in 2019-18 in the US cohort, by age and gender, in patients with chronic hepatitis C.


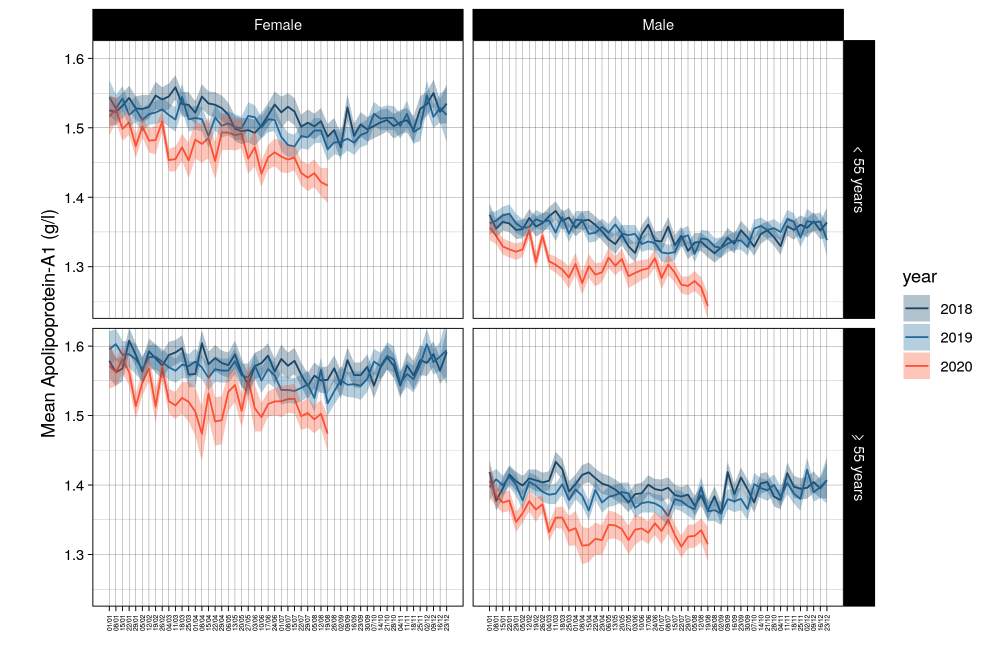


**S3D Fig.** Serum apolipoprotein ApoA1 variability during covid-19 spread versus the same days in 2019-18 in the US cohort, by age and gender, in patients with non-alcoholic fatty liver disease (NAFLD)


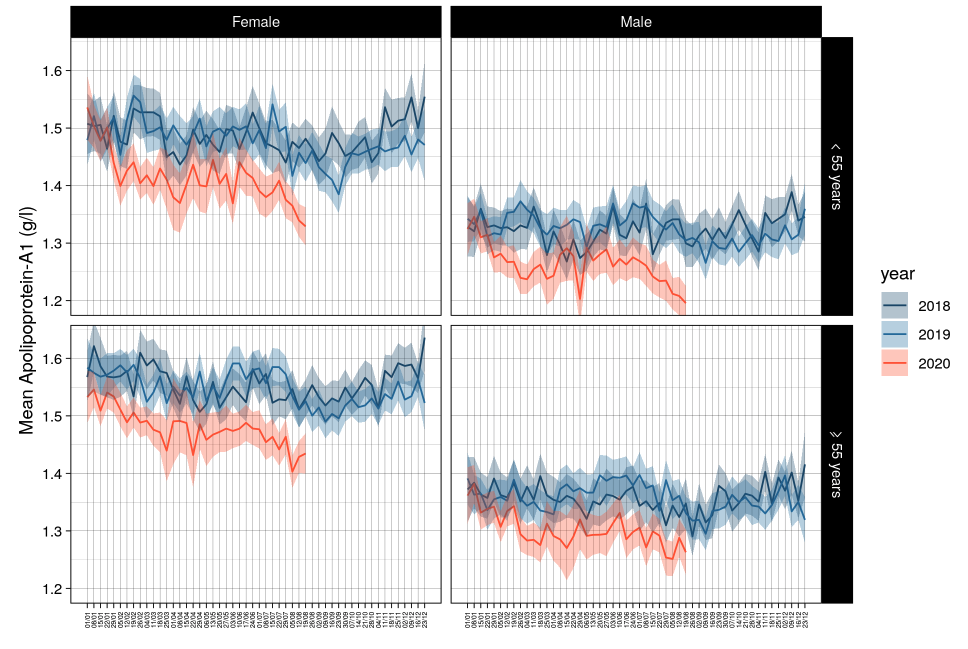

Supplement: S3 Fig — A. Serum apolipoprotein ApoA1 variability during covid-19 spread versus the same days in 2019–2018 in the APHP-PSL hospital, French and US cohorts, by gender. B. Serum apolipoprotein ApoA1 variability during covid-19 spread versus the same days in 2019–2018 in the APHP-PSL hospital, French and US cohorts, by age. C. Serum apolipoprotein ApoA1 variability during covid-19 spread versus the same days in 2019–18 in the US cohort, by age and gender, in patients with chronic hepatitis C. D. Serum apolipoprotein ApoA1 variability during covid-19 spread versus the same days in 2019–18 in the US cohort, by age and gender, in patients with non-alcoholic fatty liver disease (NAFLD). (DOCX) [file pone.0242306.s011.docx]
