# Supplementary material for: Performance of serum apolipoprotein-A1 as a sentinel of Covid-19
Source: PLoS One. 2020 Nov 20;15(11):e0242306. doi: 10.1371/journal.pone.0242306 (PMC7679025; doi:10.1371/journal.pone.0242306)

**S4 Fig.** Serum haptoglobin variability.

**S4A Fig.** Serum haptoglobin variability during covid-19 spread versus the same days in 2019-2018 in the US cohort, by age<55 years versus >= 55 years and gender

**
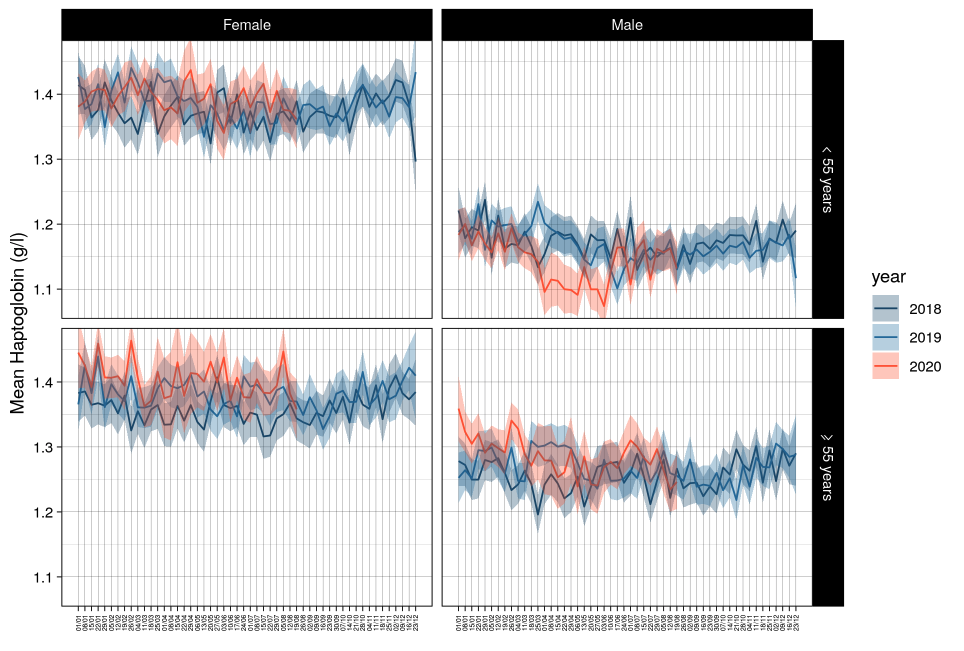
**

**S4B Fig.** Serum haptoglobin variability during covid-19 spread versus the same days in 2019-2018 in the US cohort, in NAFLD, by gender and by age <55 years versus >= 55 years.

**
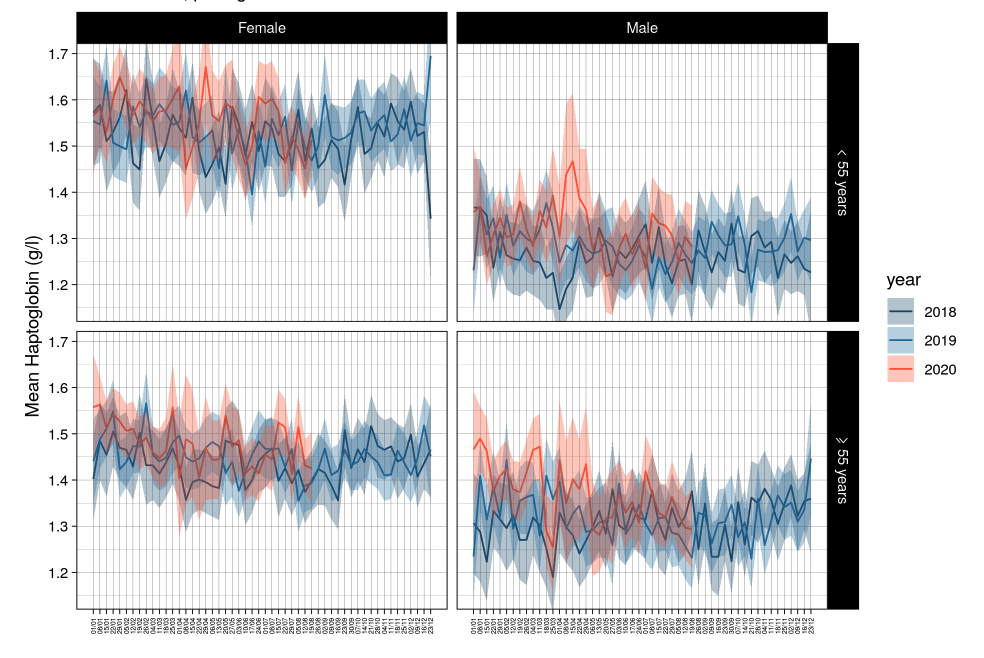
**

**S4C Fig.** Serum haptoglobin variability during covid-19 spread versus the same days in 2019-2018 in the US cohort, in HCV, by gender and by age <55 years versus >= 55 years.


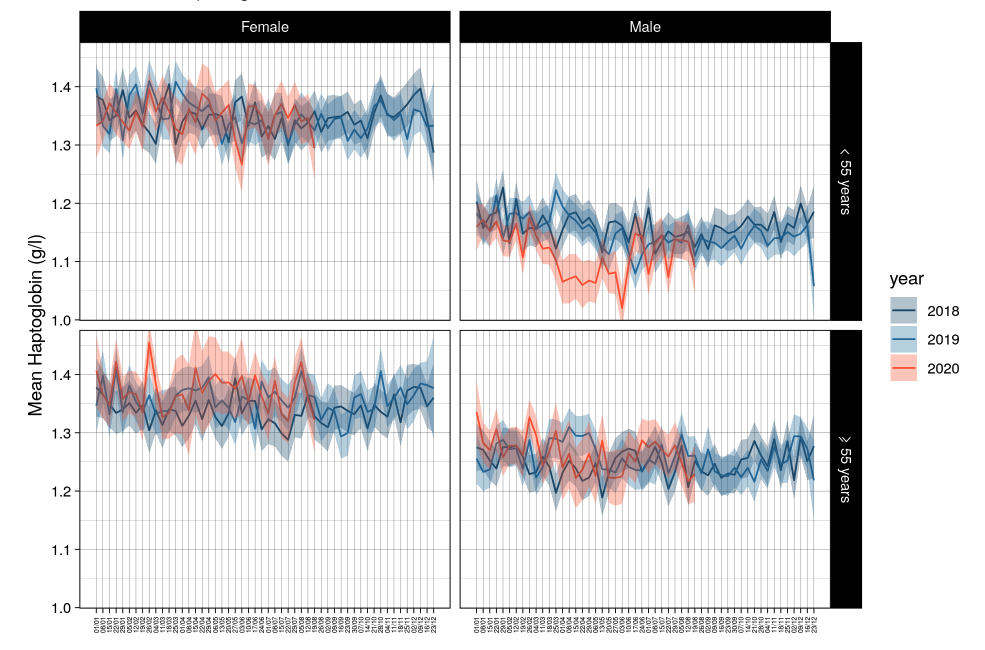

Supplement: S4 Fig — A. Serum haptoglobin variability during covid-19 spread versus the same days in 2019–2018 in the US cohort, by age<55 years versus > = 55 years and gender. B. Serum haptoglobin variability during covid-19 spread versus the same days in 2019–2018 in the US cohort, in NAFLD, by gender and by age <55 years versus > = 55 years. C. Serum haptoglobin variability during covid-19 spread versus the same days in 2019–2018 in the US cohort, in HCV, by gender and by age <55 years versus > = 55 years. (DOCX) [file pone.0242306.s012.docx]
