# Supplementary material for: Performance of serum apolipoprotein-A1 as a sentinel of Covid-19
Source: PLoS One. 2020 Nov 20;15(11):e0242306. doi: 10.1371/journal.pone.0242306 (PMC7679025; doi:10.1371/journal.pone.0242306)
Supplement: S5 Fig — A. Serum alpha-2 macroglobulin variability during covid-19 spread versus 2019 same days in APHP-PSL hospital, French cohort and US cohort. B. Serum alpha-2 macroglobulin variability during covid-19 spread versus the same days in 2019 in the US cohort, by gender and by age <55 years versus > = 55 years. C. Serum alpha-2 macroglobulin variability during covid-19 spread versus the same days in 2019 in the US cohort, by gender and by age <55 years versus > = 55 years. In NAFLD serum only. D. Serum alpha-2 macroglobulin variability during covid-19 spread versus the same days in 2019 in the US cohort, by gender and by age <55 years versus > = 55 years. In HCV serum only. (DOCX) [file pone.0242306.s013.docx]

**S5 Fig.** Serum alpha-2 macroglobulin variability

**S5A Fig.** Serum alpha-2 macroglobulin variability during covid-19 spread versus 2019 same days in APHP-PSL hospital, French cohort and US cohort


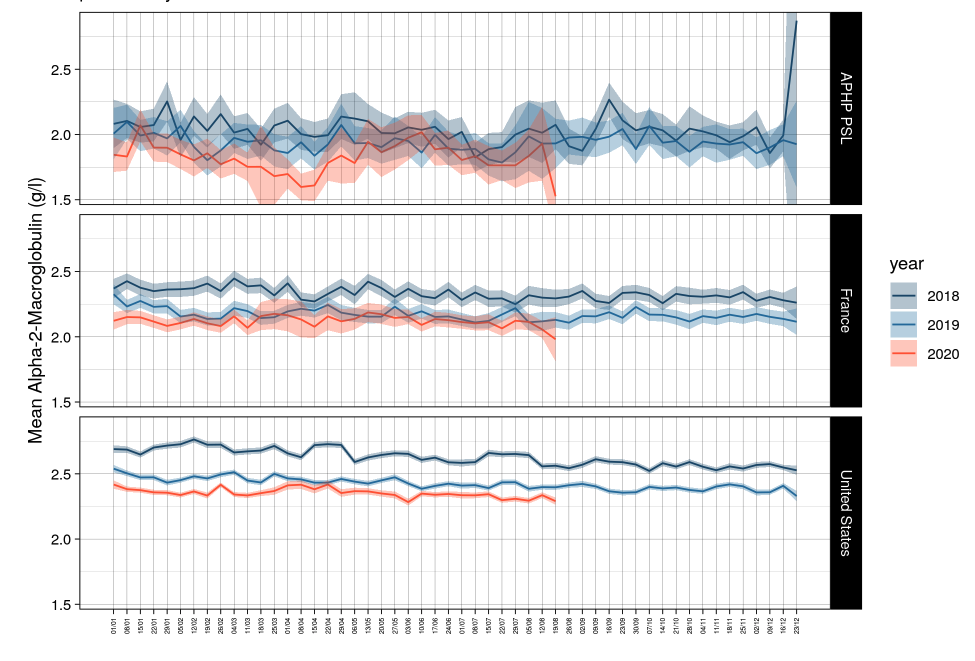


**S5B Fig.** Serum alpha-2 macroglobulin variability during covid-19 spread versus the same days in 2019 in the US cohort, by gender and by age <55 years versus >= 55 years.

**
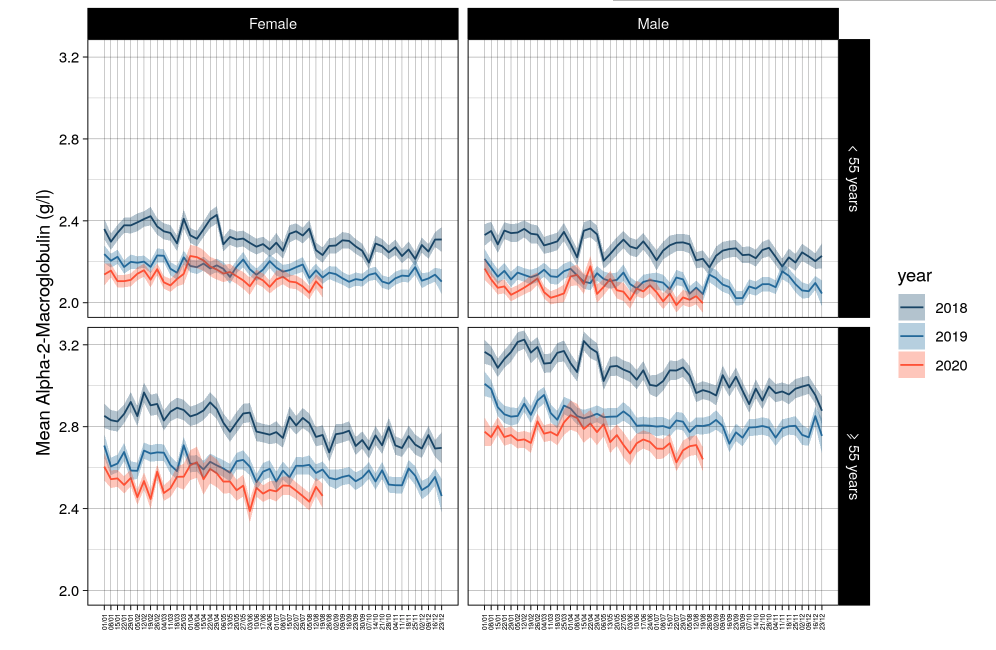
**

**S5C Fig.** Serum alpha-2 macroglobulin variability during covid-19 spread versus the same days in 2019 in the US cohort, by gender and by age <55 years versus >= 55 years. In NAFLD sera only.

**
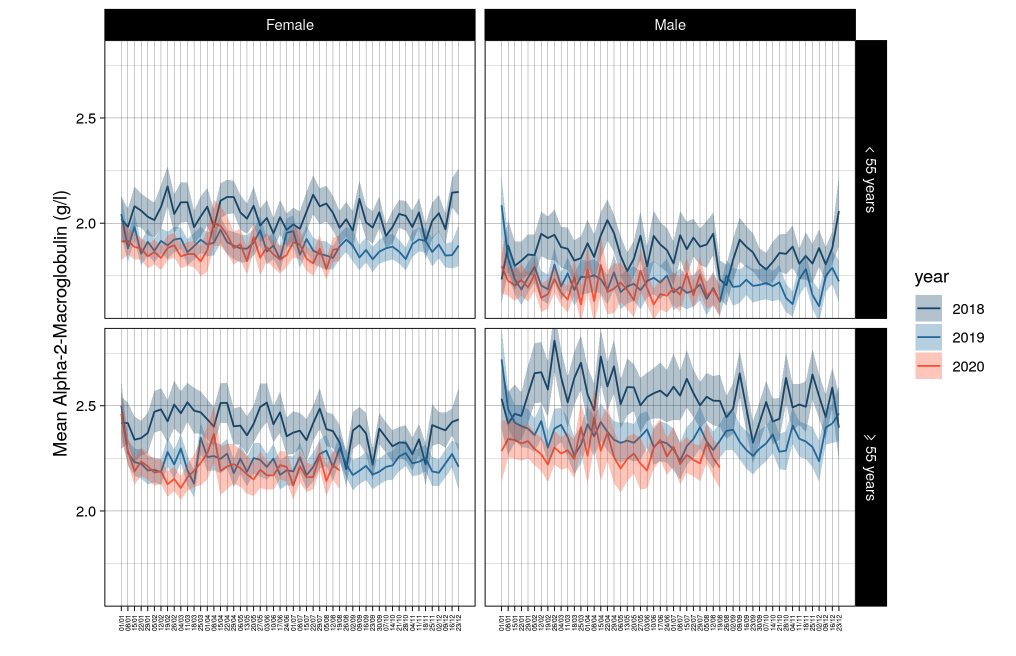
**

**S5D Fig.** Serum alpha-2 macroglobulin variability during covid-19 spread versus the same days in 2019 in the US cohort, by gender and by age <55 years versus >= 55 years. In HCV sera only.

**
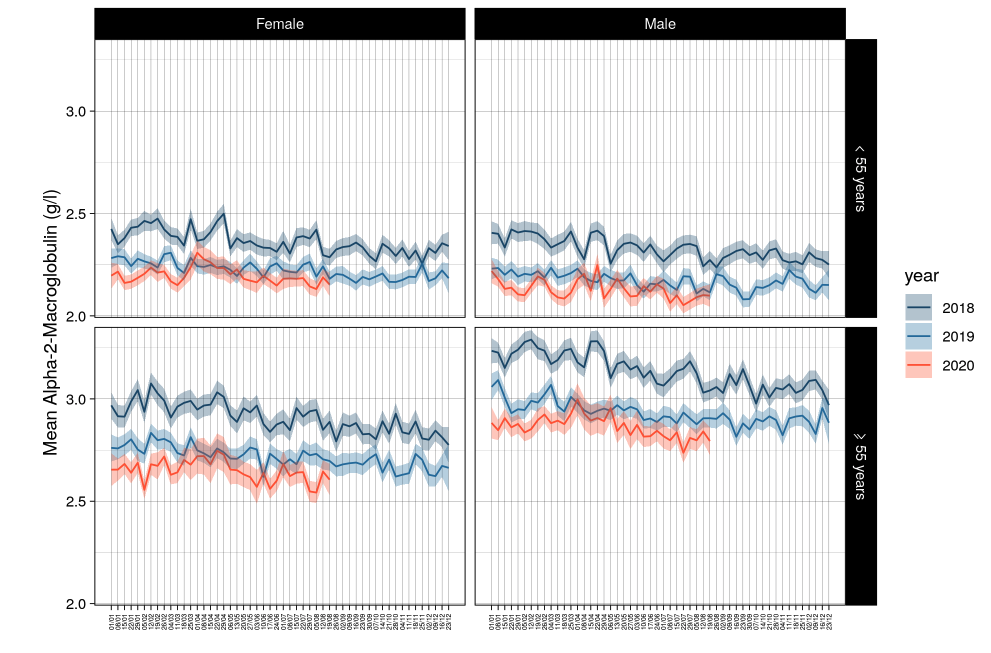
**
