# Supplementary material for: Performance of serum apolipoprotein-A1 as a sentinel of Covid-19
Source: PLoS One. 2020 Nov 20;15(11):e0242306. doi: 10.1371/journal.pone.0242306 (PMC7679025; doi:10.1371/journal.pone.0242306)
Supplement: S6 Fig — A. Serum GGT variability during covid-19 spread versus the same days in 2019 in the APHP-PSL hospital, French and US cohorts. B. Serum GGT variability during covid-19 spread versus the same days in 2019 in the US cohort, by gender, and age <55 years versus > = 55 years. (DOCX) [file pone.0242306.s014.docx]

**S6 Fig.** Serum GGT variability

**S6A Fig.** Serum GGT variability during covid-19 spread versus the same days in 2019 in the APHP-PSL hospital, French and US cohorts.

**
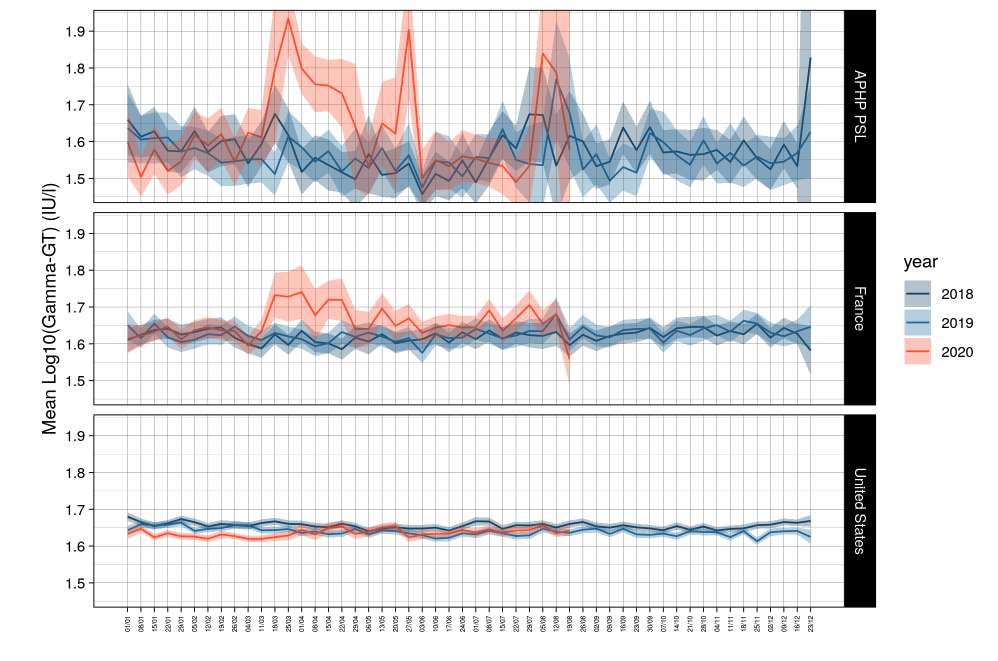
**

**S6B Fig.** Serum GGT variability during covid-19 spread versus the same days in 2019 in the US cohort, by gender, and age <55 years versus >= 55 years.

**
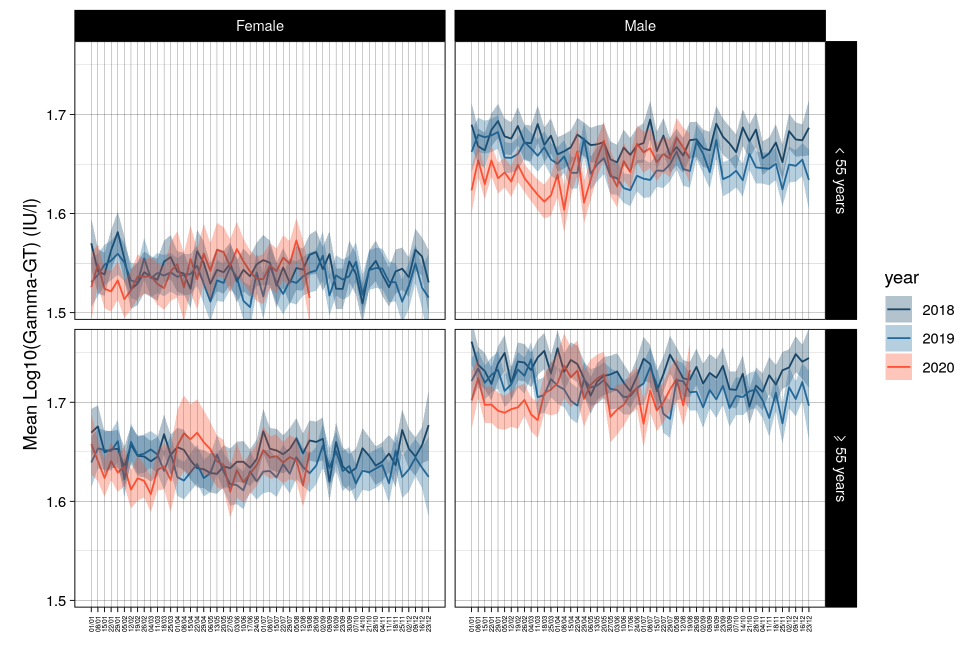
**
