# Supplementary material for: Performance of serum apolipoprotein-A1 as a sentinel of Covid-19
Source: PLoS One. 2020 Nov 20;15(11):e0242306. doi: 10.1371/journal.pone.0242306 (PMC7679025; doi:10.1371/journal.pone.0242306)
Supplement: S7 Fig — A. Serum ALT variability during covid-19 spread versus the same days in 2019 in the APHP-PSL hospital, French and US cohorts. B. Serum ALT variability during covid-19 spread versus the same days in 2019 in the US cohort, by gender and age. (DOCX) [file pone.0242306.s015.docx]

**S7 Fig.** Serum ALT variability

**S7A Fig.** Serum ALT variability during covid-19 spread versus the same days in 2019 in the APHP-PSL hospital, French and US cohorts.

**
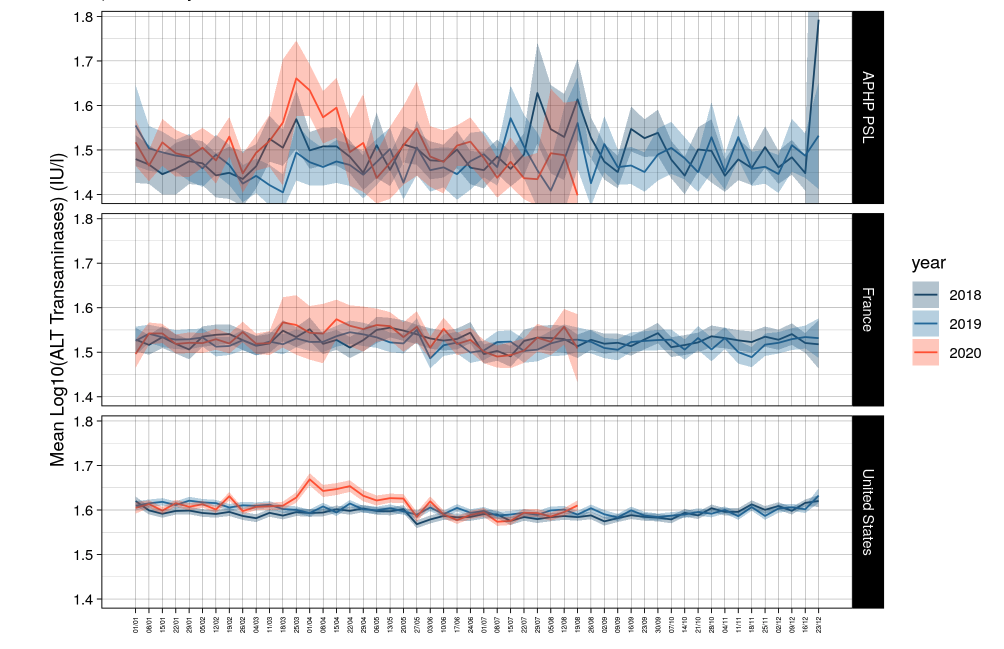
**

**S7B Fig.** Serum ALT variability during covid-19 spread versus the same days in 2019 in the US cohort, by gender and age.

**
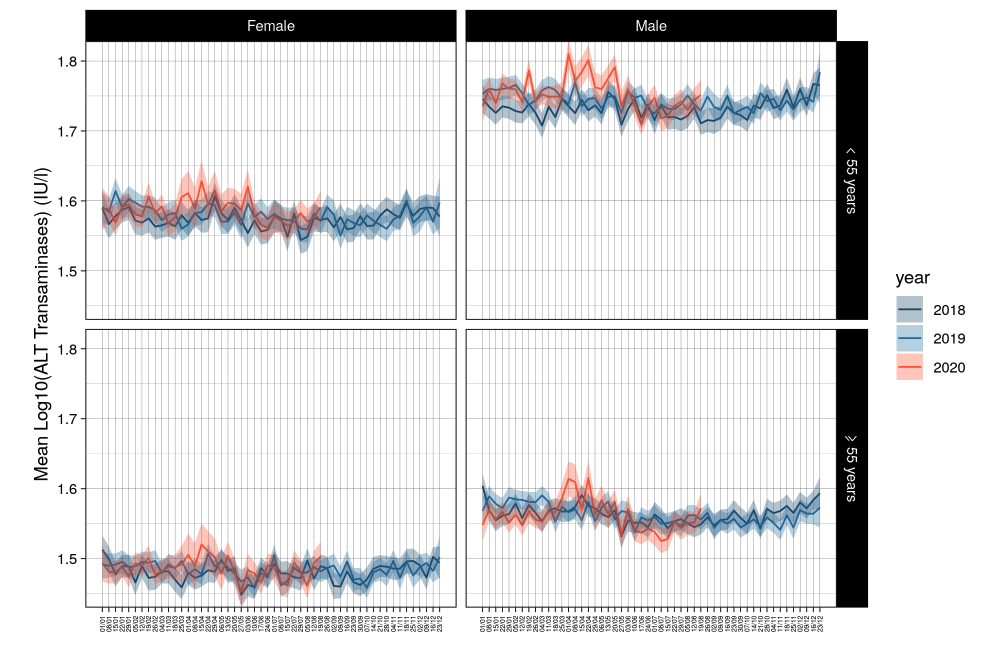
**
