# Supplementary material for: Performance of serum apolipoprotein-A1 as a sentinel of Covid-19
Source: PLoS One. 2020 Nov 20;15(11):e0242306. doi: 10.1371/journal.pone.0242306 (PMC7679025; doi:10.1371/journal.pone.0242306)

**S8 Fig.** Components without significant changes.

**S8A Fig.** Serum total bilirubin variability during covid-19 spread versus the same days in 2019 in the APHP-PSL hospital, French and US cohorts.

**
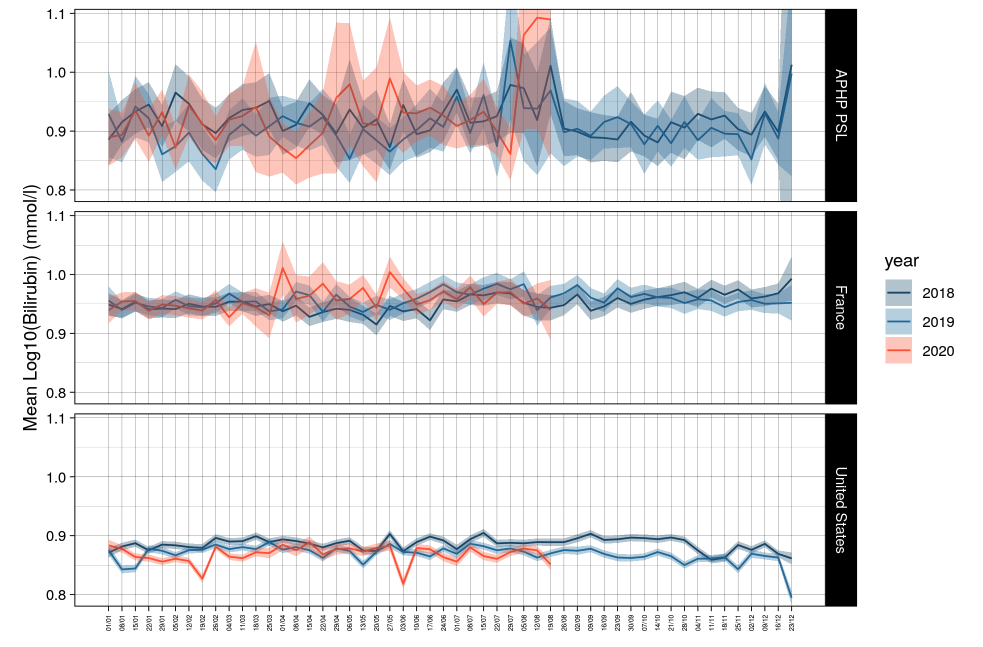
**

**S8B Fig.** Serum total cholesterol variability during covid-19 spread versus the same days in 2019 in the APHP-PSL hospital, French and US cohorts.

**
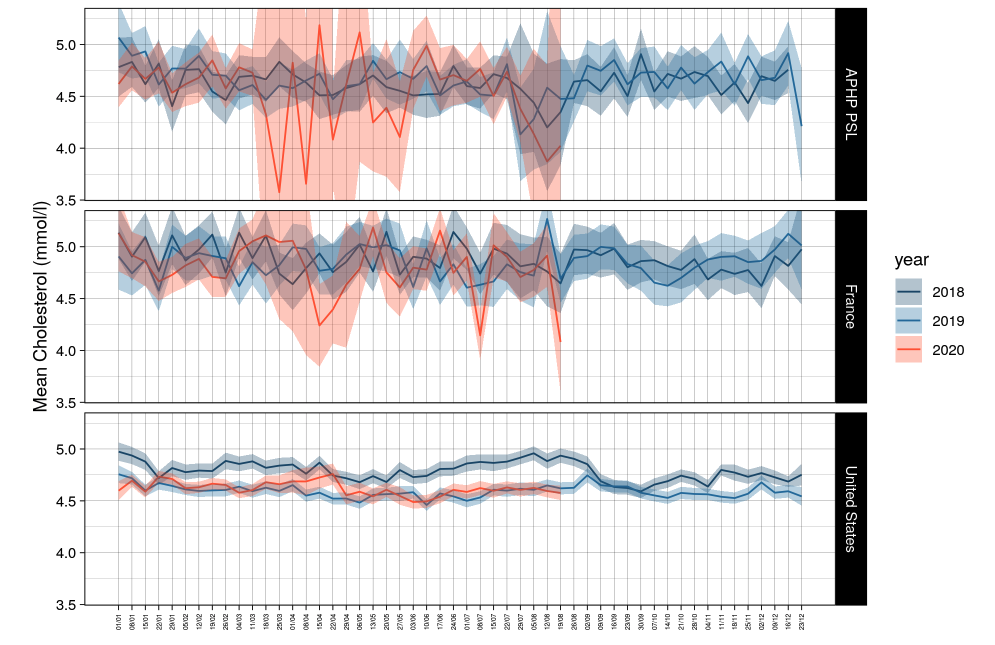
**

**S8C Fig.** Serum total triglycerides variability during covid-19 spread versus the same days in 2019 in the APHP-PSL hospital, French and US cohorts.

**
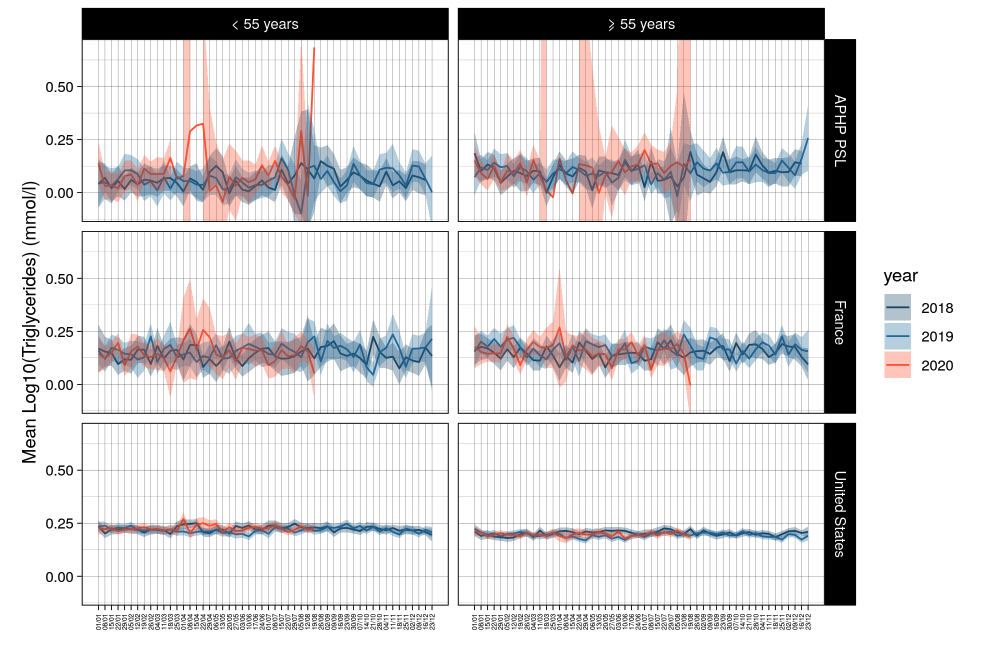
**

**S8D Fig.** Serum fasting glucose variability during covid-19 spread versus the same days in 2019 in the APHP-PSL hospital, French and US cohorts.

**
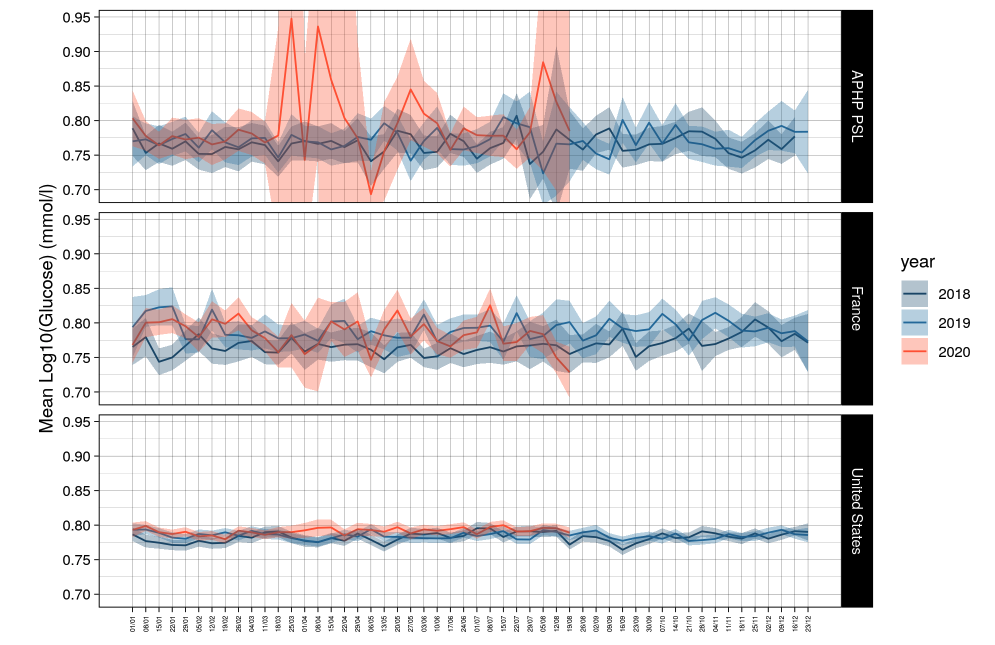
**

**S8E Fig.** Height variability during covid-19 spread versus the same days in 2019 in the APHP-PSL hospital, French and US cohorts.


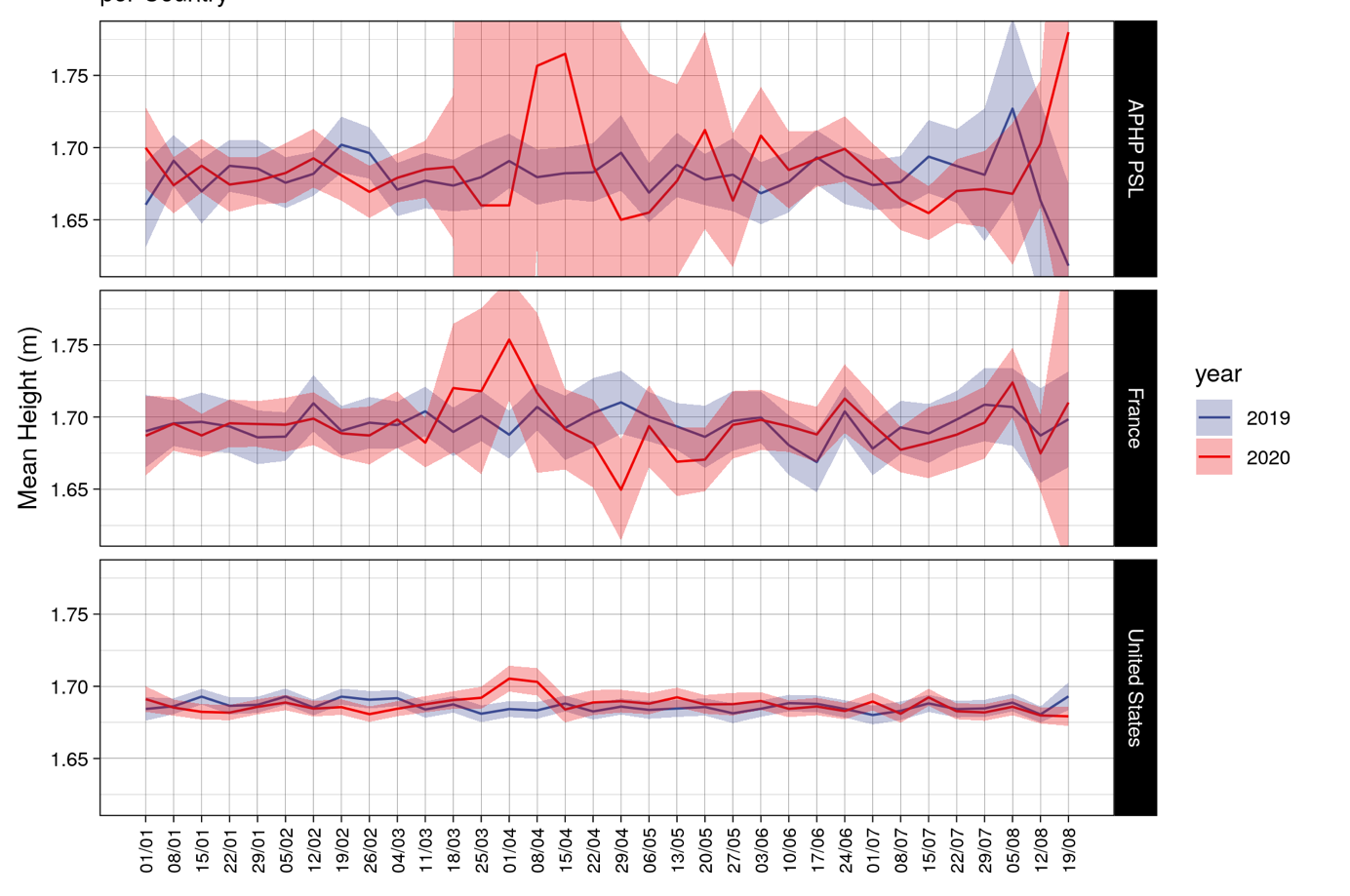


**S8F Fig.** Weight variability during covid-19 spread versus the same days in 2019 in the APHP-PSL hospital, French and US cohorts.


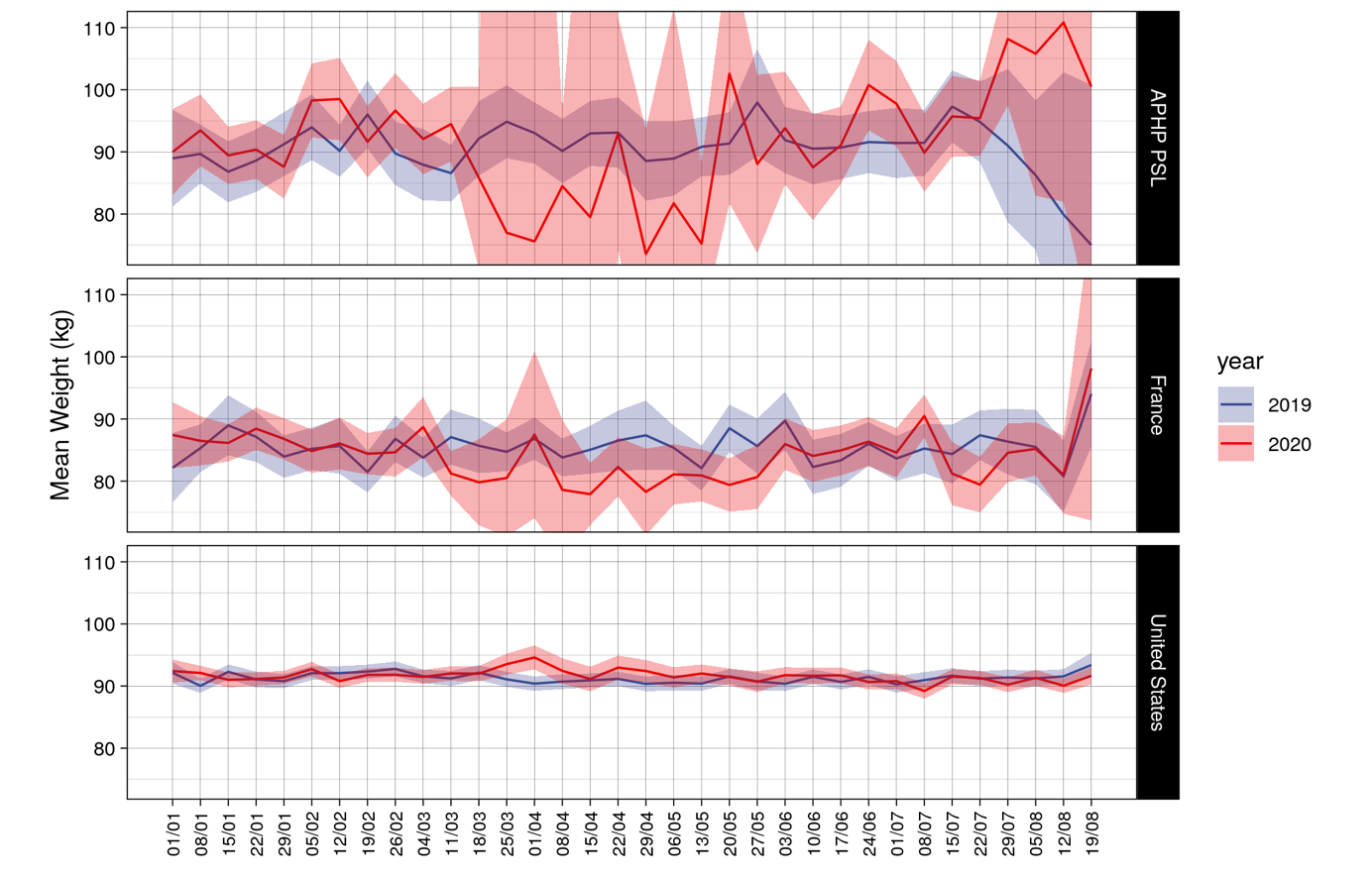

Supplement: S8 Fig — A. Serum total bilirubin variability during covid-19 spread versus the same days in 2019 in the APHP-PSL hospital, French and US cohorts. B. Serum total cholesterol variability during covid-19 spread versus the same days in 2019 in the APHP-PSL hospital, French and US cohorts. C. Serum total triglycerides variability during covid-19 spread versus the same days in 2019 in the APHP-PSL hospital, French and US cohorts. D. Serum fasting glucose variability during covid-19 spread versus the same days in 2019 in the APHP-PSL hospital, French and US cohorts. E. Height variability during covid-19 spread versus the same days in 2019 in the APHP-PSL hospital, French and US cohorts. F. Weight variability during covid-19 spread versus the same days in 2019 in the APHP-PSL hospital, French and US cohorts. (DOCX) [file pone.0242306.s016.docx]
