# Supplementary material for: Performance of serum apolipoprotein-A1 as a sentinel of Covid-19
Source: PLoS One. 2020 Nov 20;15(11):e0242306. doi: 10.1371/journal.pone.0242306 (PMC7679025; doi:10.1371/journal.pone.0242306)
Supplement: S9 Fig — A. Diagnostic performance of apolipoprotein-A1 for the diagnosis of covid-19 in 136 covid-19 cases and 393 healthy blood donors, prevalence = 26%. B. Diagnostic performance of apolipoprotein-A1 for the diagnosis of covid-19 in 136 covid-19 cases and 100 patients with rheumatological diseases, prevalence = 58%. C. Diagnostic performance of apolipoprotein-A1 for the diagnosis of covid-19 in 136 covid-19 cases and 8,335controls (Integrated database, prevalence 1.6%). (DOCX) [file pone.0242306.s017.docx]

**S9 Fig.** Sensitivity analysis of ROC curves

**S9A Fig**. Diagnostic performance of apolipoprotein-A1 for the diagnosis of covid-19 in 136 covid-19 cases and 393 healthy blood donors, prevalence=26%

**
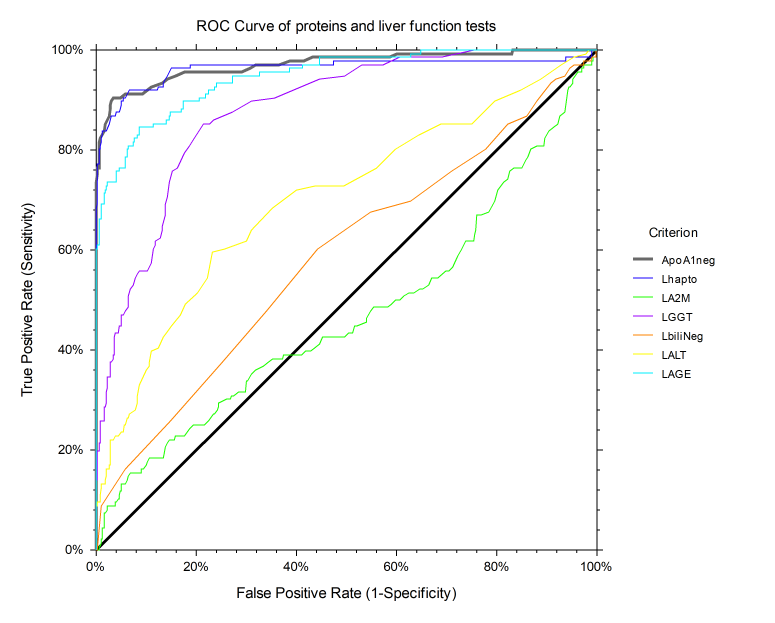
**

| **Components** | **Count** | **AUC** | **Standard Error** | **Z-value** | **P Value vs apoA1** | **Lower CI** | **Upper CI** |
| --- | --- | --- | --- | --- | --- | --- | --- |
| ApoA1neg | 529 | 0.971 | 0.009 | 50.3 | NA | 0.945 | 0.985 |
| Haptoglobin | 529 | 0.963 | 0.013 | 35.5 | <0.001 | 0.926 | 0.981 |
| A2M | 529 | 0.471 | 0.031 | -0.91 | <0.001 | 0.407 | 0.530 |
| GGT | 529 | 0.880 | 0.015 | 23.9 | <0.001 | 0.845 | 0.908 |
| Bilirubin negative | 529 | 0.587 | 0.029 | 2.9 | <0.001 | 0.526 | 0.642 |
| ALT | 529 | 0.703 | 0.027 | 7.3 | <0.001 | 0.644 | 0.753 |
| AGE | 529 | 0.948 | 0.010 | 41.2 | <0.001 | 0.921 | 0.965 |

**Diagnostic performance of apolipoprotein-A1 at the 1.25 g/L cutoff for the diagnostic of severe Covid-19, in the population at low risk of false positive, in 136 covid-19 cases and 393 healthy blood donors, prevalence=26%**

| True positive | False positive | False negative | True negative | Sensitivity | Specificity | Positive predictive value | Negative predictive value | % Covid |
| --- | --- | --- | --- | --- | --- | --- | --- | --- |
| n | n | n | n | % 95%CI | % 95%CI | % 95%CI | % 95%CI | % |
| 124 | 23 | 12 | 370 | 91.2 85.1-95.4 | 94.1 91.3-96.3 | 84.4 77.5-89.8 | 96.9 94.6-98.4 | 25.7 |
|  |  |  |  |  |  | **Adjusted on % Covid-19 estimates** | | |
|  |  |  |  | 91.2 85.1-95.4 | 94.1 91.7-92.8 | 54.7 | 99.3 | 7.2 |
|  |  |  |  | 91.2 85.1-95.4 | 94.1 91.7-92.8 | 31.0 | 99.7 | 2.8 |

**S9B Fig.** Diagnostic performance of apolipoprotein-A1 for the diagnosis of covid-19 in 136 covid-19 cases and 100 patients with rheumatological diseases, prevalence=58%


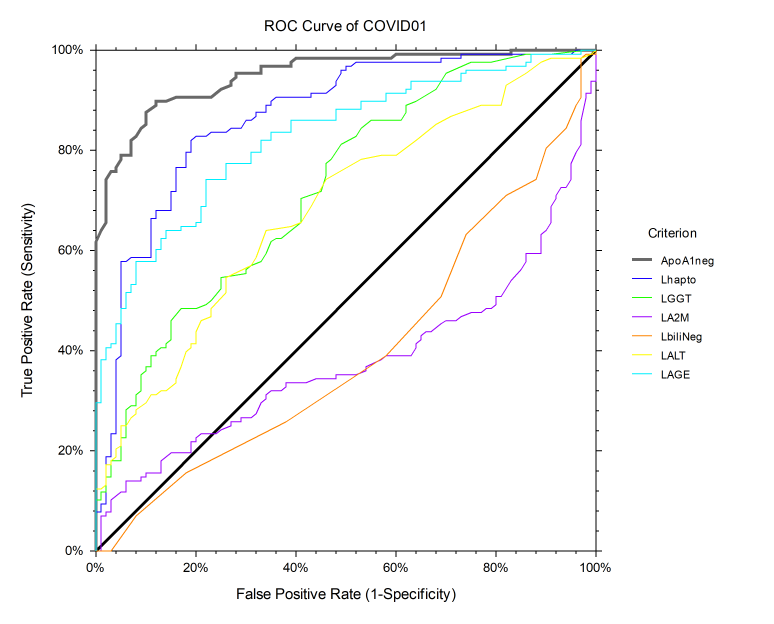


| **Components** | **Count** | **AUC** | **Standard Error** | **Z-value** | **P-Value vs ApoA1** | **Lower CI** | **Upper CI** |
| --- | --- | --- | --- | --- | --- | --- | --- |
| ApoA1 negative | 236 | 0.9528 | 0.0123 | 36.836 | NA | 0.922 | 0.971 |
| Haptoglobin | 236 | 0.8640 | 0.0249 | 14.592 | <0.001 | 0.806 | 0.906 |
| A2M | 236 | 0.4147 | 0.0372 | -2.293 | <0.001 | 0.339 | 0.485 |
| GGT | 236 | 0.7138 | 0.0336 | 6.368 | <0.001 | 0.642 | 0.775 |
| Bilirubin negative | 236 | 0.3865 | 0.0365 | -3.111 | <0.001 | 0.313 | 0.456 |
| ALT | 236 | 0.6419 | 0.0359 | 3.957 | <0.001 | 0.5661 | 0.707 |
| AGE | 236 | 0.8263 | 0.0263 | 12.425 | <0.001 | 0.7675 | 0.871 |

**Diagnostic performance of apolipoprotein-A1 at the 1.25 g/L cutoff for the diagnostic of severe Covid-19, in the population at low risk of false positive, in 136 covid-19 cases and 100 patients with rheumatological diseases, prevalence=58%**

| True positive | False positive | False negative | True negative | Sensitivity | Specificity | Positive predictive value | Negative predictive value | % Covid |
| --- | --- | --- | --- | --- | --- | --- | --- | --- |
| n | n | n | n | % 95%CI | % 95%CI | % 95%CI | % 95%CI | % |
| 124 | 16 | 12 | 84 | 91.2 85.1-95.4 | 84.0 91.3-96.3 | 88.6 82.1-93.3 | 87.5 79.2-93.4 | 57.6 |
|  |  |  |  |  |  | **Adjusted on % Covid-19 estimates** | | |
|  |  |  |  | 91.2 85.1-95.4 | 84.0 91.7-92.8 | 30.7 | 99.2 | 7.2 |
|  |  |  |  | 91.2 85.1-95.4 | 84.0 91.7-92.8 | 14.1 | 99.7 | 2.8 |

**S9C Fig.** Diagnostic performance of apolipoprotein-A1 for the diagnosis of covid-19 in 136 covid-19 cases and 8,335controls (Integrated database, prevalence 1.6%))

**
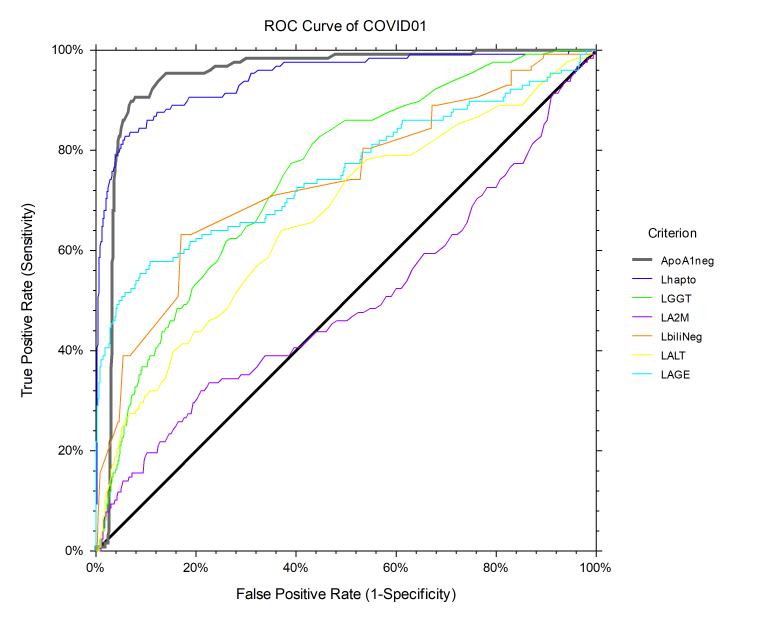
**

| **Criterion** | **Count** | **AUC** | **Standard Error** | **Z-value** | **P-Value vs apoA1** | **95%CI Lower** | **95%CI Upper** |
| --- | --- | --- | --- | --- | --- | --- | --- |
| ApoA1 negative | 8471 | 0.9470 | 0.0074 | 60.264 | <0.001 | 0.9304 | 0.9598 |
| Haptoglobin | 8471 | 0.9386 | 0.0140 | 0.554 | 0.58 | 0.9042 | 0.9609 |
| A2M | 8471 | 0.5287 | 0.0291 | 13.85 | <0.001 | 0.4694 | 0.5833 |
| GGT | 8471 | 0.7409 | 0.0201 | 9.59 | <0.001 | 0.6988 | 0.7778 |
| Bilirubin negative | 8471 | 0.7112 | 0.0265 | 8.26 | <0.001 | 0.6553 | 0.7593 |
| ALT | 8471 | 0.6230 | 0.0272 | 11.31 | <0.001 | 0.5668 | 0.6734 |
| AGE | 8471 | 0.7707 | 0.0269 | 6.47 | <0.001 | 0.7125 | 0.8184 |

**Diagnostic performance of apolipoprotein-A1 at the 1.25 g/L cutoff for the diagnostic of severe Covid-19, in the integrated population at high risk of false positive.**

| True positive | False positive | False negative | True negative | Sensitivity | Specificity | Positive predictive value | Negative predictive value | % Covid |
| --- | --- | --- | --- | --- | --- | --- | --- | --- |
| n | n | n | n | % 95%CI | % 95%CI | % 95%CI | % 95%CI | % |
| 124 | 646 | 12 | 7689 | 91.2 85.1-95.4 | 92.2 91.7-92.8 | 16.1 13.6-18.9 | 99.8 99.7-99.9 | 1.6 % |
|  |  |  |  |  |  | **Adjusted on % Covid-19 estimates** | | |
|  |  |  |  | 91.2 85.1-95.4 | 92.2 91.7-92.8 | 47.7 | 99.3 | 7.2 % |
|  |  |  |  | 91.2 85.1-95.4 | 92.2 91.7-92.8 | 25.3 | 99.7 | 2.8 % |
